# Wavenumber-Explicit *hp*-FEM Analysis for Maxwell’s Equations with Impedance Boundary Conditions

**DOI:** 10.1007/s10208-023-09626-7

**Published:** 2023-11-14

**Authors:** J. M. Melenk, S. A. Sauter

**Affiliations:** 1 Institut für Analysis und Scientific Computing, Technische Universität Wien, Wiedner Hauptstrasse 8-10, 1040 Vienna, Austria; 2 Institut für Mathematik, Universität Zürich, Winterthurerstr. 190, 8057 Zurich, Switzerland

**Keywords:** Maxwell’s equations, Time-harmonic, High-frequency, Wavenumber explicit, *hp*-FEM, Quasi-optimality, 35J05, 65N12, 65N30

## Abstract

The time-harmonic Maxwell equations at high wavenumber *k* in domains with an analytic boundary and impedance boundary conditions are considered. A wavenumber-explicit stability and regularity theory is developed that decomposes the solution into a part with finite Sobolev regularity that is controlled uniformly in *k* and an analytic part. Using this regularity, quasi-optimality of the Galerkin discretization based on Nédélec elements of order *p* on a mesh with mesh size *h* is shown under the *k*-explicit scale resolution condition that (a) *kh*/*p* is sufficient small and (b) $p/\ln k$ is bounded from below.

## Introduction

The time-harmonic Maxwell equations at high wavenumber *k* are a fundamental component of high-frequency computational electromagnetics. Computationally, these equations are challenging for several reasons. The solutions are highly oscillatory so that fine discretizations are necessary and correspondingly large computational resources are required. While conditions to resolve the oscillatory nature of the solution appear unavoidable, even more stringent conditions on the discretizations have to be imposed for stability reasons: In many numerical methods based on the variational formulation of Maxwell’s equations, the gap between the actual error and the best approximation error widens as the wavenumber *k* becomes large. This “pollution effect” is a manifestation of a lack of coercivity of the problem, as is typical in time-harmonic wave propagation problems. Mathematically understanding this “pollution effect” in terms of the wavenumber *k* and the discretization parameters for the model problem (1.1) is the purpose of the present work.

The “pollution effect”, i.e., the fact that discretizations of time-harmonic wave propagation problems are prone to dispersion errors, is probably best studied for the Helmholtz equation at large wavenumbers. The beneficial effect of using high order methods was numerically observed very early and substantiated for translation-invariant meshes [1, 2]; a rigorous mathematical analysis for unstructured meshes was developed in the last decade only in [17, 37, 38]. These works analyze high order FEM (*hp*-FEM) for the Helmholtz equation in a Gårding setting using duality techniques. This technique, often called “Schatz argument”, crucially hinges on the regularity of the dual problem, which is again a Helmholtz problem. The key new insight of the line of work [17, 37, 38] is a refined wavenumber-explicit regularity theory for Helmholtz problems that takes the following form (“regularity by decomposition”): given data, the solution *u* is written as $u_{H^{2}}+u_{{\mathcal {A}}}$ where $u_{H^{2}}$ has the regularity expected of elliptic problems and is controlled in terms of the data with constants *independent* of *k*. The part $u_{{\mathcal {A}}}$ is a (piecewise) analytic function whose regularity is described explicitly in terms of *k*. Employing “regularity by decomposition” for the analysis of discretizations has been successfully applied to other Helmholtz problems and discretizations such DG methods [31], BEM [27], FEM-BEM coupling [29], and heterogeneous Helmholtz problems [4, 9, 25, 26].

In this paper, we consider the following time-harmonic Maxwell equations with impedance boundary conditions as our model problem:

1.1a $$\begin{aligned} {\text {curl}}\,{\text {curl}}\,{{\textbf{E}}}-k^{2}{{\textbf{E}}}&={{\textbf{f}}}\quad \text{ in } \varOmega , \end{aligned}$$

1.1b $$\begin{aligned} ({\text {curl}}\,{{\textbf{E}}})\times {{\textbf{n}}}-{\text {i}}\, k{{\textbf{E}}}_{T}&={{\textbf{g}}}_{T}\quad \text{ on } \partial \varOmega \end{aligned}$$

on a bounded Lipschitz domain $\varOmega \subset \mathbb {R}^{3}$ with simply connected boundary $\partial \varOmega$. We study an ${{\textbf{H}}} ({\text {curl}})$-conforming Galerkin method with elements of degree *p* on a mesh of size *h* and show quasi-optimality of the method under the *scale resolution condition*

1.2 $$\begin{aligned} \frac{\left| k\right| h}{p}\le c_{1}\qquad \text{ and } \qquad p\ge c_{2}\ln \left| k\right| , \end{aligned}$$

where $c_{2}$ $>0$ is arbitrary and $c_{1}$ $>0$ is sufficiently small (Theorem 9.7). The resolution condition $\left| k\right| h/p\le c_{1}$ is a natural condition to resolve the oscillatory behavior of the solution, and the side constraint $p\ge c_{2}\ln \left| k\right|$ is a rather weak condition that suppresses the “pollution effect”.

Compared to the scalar Helmholtz case, where the compact embedding $H^1 \subset L^2$ underlies the success of the duality argument, the convergence analysis of discretizations of Maxwell’s equations is hampered by the fact that the embedding ${\textbf{H}}({\text {curl}}) \subset {\textbf{L}}^2$ is not compact so that a duality argument is not immediately applicable. This issue arises even in the context of convergence analyses that are not explicit in the wavenumber *k*. An analysis can be based on the observation that ${\textbf{H}}({\text {curl}}) \cap {\textbf{H}}({\text {div}})$ endowed with appropriate boundary conditions is compactly embedded in ${\textbf{L}}^2$. This approach, which is structurally described in [39, Sec. 1.2], involves as a first ingredient the ability to decompose discrete functions into gradient parts and (discrete) solenoidal parts in two ways, namely, on the continuous level and the discrete level. The solenoidal part of the decomposition on the continuous level is in ${\textbf{H}}({\text {curl}}) \cap {\textbf{H}}({\text {div}})$ and admits a duality argument. Galerkin orthogonalities are invoked to then reduce the analysis to that of the difference between the solenoidal parts of the continuous and the discrete level. For the analysis of this difference, a second ingredient is vital, namely, special interpolation operators with a commuting diagram property. These two ingredients underlie many duality arguments for Maxwell problems in the literature, see, e.g., [41, Sec. 7.2], [8, 10, 16, 56] and references therein. The present work follows [41, Sec. 7.2] and the path outlined in [39, Sec. 1.1–1.3].

At the heart of the *k*-explicit convergence analysis for (1.1) is a *k*-explicit regularity theory for the above mentioned dual problem. Similarly to the Helmholtz case discussed above, it takes the form of a “regularity by decomposition” (Theorem 7.3). Such a regularity theory was developed for Maxwell’s equations in full space in the recent paper [39], where the decomposition is directly accessible in terms of the Newton potential and layer potentials. For the present bounded domain case, however, an explicit construction of the decomposition is not available, and the iterative construction as in the Helmholtz case of [38] has to be brought to bear. For this, a significant complication in the Maxwell case compared to the Helmholtz case arises from the requirement that the frequency filters used in the construction be such that they produce solenoidal fields if the argument is solenoidal.

While our wavenumber-explicit regularity result Theorem 7.3 underlies our proof of quasioptimal convergence of the high order Galerkin method (cf. Theorem 9.7), it also proves useful for wavenumber-explicit interpolation error estimates as worked out in Corollary 9.8.

The present paper analyzes an ${{\textbf{H}}}({\text {curl}})$-conforming discretization based on high order Nédélec elements. Various other high order methods for Maxwell’s equations that are explicit in the wavenumber can be found in the literature. Closest to our work are [11, 45]. The work [45] studies the same problem (1.1) but uses an ${{\textbf{H}}}^{1}$-based instead of an ${{\textbf{H}}}({\text {curl}} )$-based variational formulation involving both the electric and the magnetic field. The proof of quasi-optimality in [45] is based on a “regularity by decomposition” technique similar to the present one. [44] studies the same ${{\textbf{H}}}^{1}$-based variational formulation and ${{\textbf{H}}}^{1}$-conforming discretizations for (1.1) on certain polyhedral domains and obtains *k*-explicit conditions on the discretization for quasi-optimality. Key to this is a description of the solution regularity in [44] in terms of corner and edge singularities. The work [11] studies fixed (but arbitrary) order ${{\textbf{H}}}({\text {curl}})$-conforming discretizations of heterogeneous Maxwell problems and shows a similar quasi-optimality result by generalizing the corresponding Helmholtz result [9]; the restriction to finite order methods compared to the present work appears to be due to the difference in which the decomposition of solutions of Maxwell problems is obtained. High order Discontinuous Galerkin (DG) and Hybridizable DG (HDG) methods for (1.1) have been presented in [18] and [28] together with a stability analysis that is explicit in *h*, *k*, and *p*. A dispersion analysis of high order methods on tensor-product meshes is given in [2].

The outline of the paper is as follows. Section 2 introduces the notation and tools such as regular decompositions (see Sect. 2.4) that are indispensable for the analysis of Maxwell problems. Section 3 (Theorem 3.7) shows that the solution of (1.1) depends only polynomially on the wavenumber *k*. This stability result is obtained using layer potential techniques in the spirit of earlier work [17, Thm. 2.4] for the analogous Helmholtz equation. While earlier stability estimates for (1.1) in [18, 23, 55], and [44, Thm. 5.2 ] are obtained by judicious choices of test functions and rely on star-shapedness of the geometry, Theorem 3.7 does not require star-shapedness. It is worth mentioning that at least in the analogous case of the Helmholtz equation, alternatives to the use of suitable test functions or layer potential exist, which can lead to better *k*-dependencies; we refer to [51] for results and a discussion. Section 4 analyzes a “sign definite” Maxwell problem and presents *k*-explicit regularity assertions for it (Theorem 4.3). The motivation for studying this particular boundary value problem is that, since the principal parts of our sign-definite Maxwell operator and that of (1.1) coincide, a contraction argument can be brought to bear in the proof of Theorem 7.3. A similar technique has recently been used for heterogeneous Helmholtz problems in [4]. Section 5 collects *k*-explicit regularity assertions for (1.1) (Lemma 5.1 for finite regularity data and Theorem 5.2 for analytic data). The contraction argument in the proof of Theorem 7.3 relies on certain frequency splitting operators (both in the volume and on the boundary), which are provided in Sect. 6. Section 7 presents the main analytical result, Theorem 7.3, where the solution of (1.1) with finite regularity data ${\textbf{f}}$, ${\textbf{g}}$ is decomposed into a part with finite regularity but *k*-uniform bounds, a gradient field, and an analytic part. Section 8 presents the discretization of (1.1) based on high order Nédélec elements and presents *hp*-approximation operators that map into Nédélec spaces. These operators are the same ones as used in [39] but we work out their approximation properties on the skeleton of the mesh since stronger approximation properties on the boundary $\partial \varOmega$ are required in the present case of impedance boundary conditions. Section 9 shows quasi-optimality (Theorem 9.7) under the scale resolution condition (1.2). Section 10 concludes the paper with numerical results.

## Setting

### Geometric Setting and Sobolev Spaces on Lipschitz Domains

Let $\varOmega \subset {\mathbb {R}}^{3}$ be a bounded Lipschitz domain which we assume throughout the paper to have a simply connected and sufficiently smooth boundary $\varGamma :=\partial \varOmega$; if less regularity is required, we will specify this. We flag already at this point that the main quasi-optimal convergence result, Theorem 9.7 will require analyticity of $\varGamma$. The outward unit normal vector field is denoted by ${\textbf{n}}:\varGamma \rightarrow {\mathbb {S}}_{2}$.

The Maxwell problem in the frequency domain involves the wavenumber (denoted by *k*) and we assume that^1^

2.1 $$\begin{aligned} k\in {\mathbb {R}}\backslash \left( -k_{0},k_{0}\right) \quad \text {for }k_{0}=1. \end{aligned}$$

Let $L^{2}(\varOmega )$ denote the usual Lebesgue space on $\varOmega$ with scalar product $\left( \cdot ,\cdot \right) _{L^{2}(\varOmega )}$ and norm $\left\| \cdot \right\| _{L^{2}(\varOmega )}:=\left( \cdot ,\cdot \right) _{L^{2} (\varOmega )}^{1/2}$. Recall that the complex conjugation is applied to the second argument in $\left( \cdot ,\cdot \right) _{L^{2}(\varOmega )}$. If the domain $\varOmega$ is clear from the context we write short $\left( \cdot ,\cdot \right)$, $\left\| \cdot \right\|$ for $\left( \cdot ,\cdot \right) _{L^{2}\left( \varOmega \right) }$, $\left\| \cdot \right\| _{L^{2}(\varOmega )}$. If necessary, $(\cdot ,\cdot )_{L^2(\varOmega )}$ (or $(\cdot ,\cdot )$) is understood as the extended $L^2(\varOmega )$ inner product, i.e., a duality pairing with pivot space $L^2(\varOmega )$. For Sobolev spaces, we follow the notation of [30]. For $s \ge 0$ we denote by $H^{s}(\varOmega )$ the usual Sobolev spaces of index *s* with norm $\left\| \cdot \right\| _{H^{s}\left( \varOmega \right) }$ and by ${\widetilde{H}}^s(\varOmega ) = H^s_{\overline{\varOmega }}({{\mathbb {R}}}^3)$ the space of Sobolev functions on ${{\mathbb {R}}}^3$ with support in $\overline{\varOmega }$. For $s \ge 0$, $H^{-s}(\varOmega )$ denotes the dual of ${\widetilde{H}}^s(\varOmega )$. The space ${\textbf{H}}^s(\varOmega )$ of vector-valued functions is characterized by componentwise membership in $H^s(\varOmega )$. We write $(\cdot ,\cdot )$ also for the vectorial ${\textbf{L}}^{2}(\varOmega )$ inner product given by $({\textbf{f}},{\textbf{g}})=\int _{\varOmega }\langle {\textbf{f}},\overline{{\textbf{g}}}\rangle$. Here, we introduce for vectors $\textbf{a,b}\in {\mathbb {C}}^{3}$ with ${\textbf{a}}=(a_{j})_{j=1}^{3}$, $\mathbf {b=}(b_{j})_{j=1}^{3}$ the bilinear form $\langle \cdot ,\cdot \rangle$ by $\langle {\textbf{a}},{\textbf{b}}\rangle :=\sum _{j=1}^{3}a_{j}b_{j}$. For $m\in {\mathbb {N}}_{0}$, we introduce the seminorms

2.2 $$\begin{aligned} \left| {\textbf{f}}\right| _{{\textbf{H}}^{m}(\varOmega )}:=\left( \sum _{\alpha \in {\mathbb {N}}_{0}^{3}:|\alpha |=m}\frac{|\alpha |!}{\alpha !}\left( \partial ^{\alpha }{\textbf{f}},\partial ^{\alpha }{\textbf{f}}\right) \right) ^{1/2} \end{aligned}$$

and the full norms $\Vert {\textbf{f}}\Vert _{{\textbf{H}}^{m}(\varOmega )}^{2}:=\sum _{n=0}^{m}|{\textbf{f}}|_{{\textbf{H}}^{n}(\varOmega )}^{2}$. For the Maxwell problem the space ${\textbf{H}}({\text {curl}})$ is the key to describe the energy of the electric field. For $m\in {\mathbb {N}}_{0}$ we set

2.3a $$\begin{aligned} {\textbf{H}}^{m}\left( {\text {curl}},\varOmega \right)&:=\left\{ {\textbf{u}}\in {\textbf{H}}^{m}( \varOmega ) \mid {\text {curl}}\, {\textbf{u}}\in {\textbf{H}}^{m}( \varOmega ) \right\} \quad \text {and} \end{aligned}$$

2.3b $$\begin{aligned} {\textbf{X}}&:={\textbf{H}}( {\text {curl}},\varOmega ) :={\textbf{H}} ^{0}( {\text {curl}},\varOmega ) . \end{aligned}$$

The space ${\textbf{H}}^{m}({\text {div}},\varOmega )$ is given for $m\in {\mathbb {N}}_{0}$ by

2.4 $$\begin{aligned} {\textbf{H}}^{m}( {\text {div}},\varOmega ):=\left\{ {\textbf{u}}\in {\textbf{H}}^{m}( \varOmega ) \mid {\text {div}}\,{\textbf{u}}\in H^{m}( \varOmega ) \right\} \end{aligned}$$

with ${\textbf{H}}({\text {div}},\varOmega ):={\textbf{H}}^{0}({\text {div}},\varOmega )$. We introduce

2.5 $$\begin{aligned} {\textbf{H}}(\mathop {{\text {div}}}\nolimits _{0},\varOmega ):=\left\{ {\textbf{u}} \in {\textbf{H}}( {\text {div}},\varOmega ) \mid {\text {div}}\,{\textbf{u}}=0\right\} . \end{aligned}$$

For $\rho \in {\mathbb {R}}\setminus \{ 0\}$ and *m*, $\ell \in {\mathbb {N}}_{0}$ we define the indexed norms and seminorms by

2.6 $$\begin{aligned} \left| {\textbf{v}}\right| _{{\textbf{H}}^{\ell }(\varOmega ),\rho }:=\left| \rho \right| ^{-\ell }\left| {\textbf{v}}\right| _{{\textbf{H}}^{\ell }(\varOmega )}\quad \text {and}\quad \left\| {\textbf{v}}\right\| _{{\textbf{H}} ^{m}(\varOmega ),\rho }:=\left( \sum _{\ell =0}^{m}\left| {\textbf{v}}\right| _{{\textbf{H}}^{\ell }(\varOmega ),\rho }^{2}\right) ^{1/2} \end{aligned}$$

and corresponding dual norms

2.7 $$\begin{aligned} \left\| {\textbf{v}}\right\| _{{\textbf{H}}^{-m}(\varOmega ),\rho }:=\left| \rho \right| ^{m}\left\| {\textbf{v}}\right\| _{{\textbf{H}}^{-m} (\varOmega )}. \end{aligned}$$

We define for ${\text {D}}\in \left\{ {\text {curl}},{\text {div}}\right\}$

$$\begin{aligned} \left\| {\textbf{f}}\right\| _{{\textbf{H}}^{m}({\text {D}} ,\varOmega ),\rho }&:=\left( \rho ^{-2m}\left| {\text {D}}\, {\textbf{f}}\right| _{{\textbf{H}}^{m}(\varOmega )}^{2}+\rho ^{2}\left\| {\textbf{f}}\right\| _{{\textbf{H}}^{m}(\varOmega ),\rho }^{2}\right) ^{1/2}\\&=\left( \rho ^{-2m}\left| {\text {D}}\,{\textbf{f}}\right| _{{\textbf{H}}^{m}\left( \varOmega \right) }^{2}+\sum _{\ell =0}^{m}\rho ^{2-2\ell }\left| {\textbf{f}}\right| _{{\textbf{H}}^{\ell }(\varOmega )}^{2}\right) ^{1/2} \end{aligned}$$

and introduce the shorthands:

2.8 $$\begin{aligned} \left\| {\textbf{f}}\right\| _{{\textbf{H}}^{m}\left( {\text {D}} ,\varOmega \right) }&:=\left\| {\textbf{f}}\right\| _{{\textbf{H}} ^{m}\left( {\text {D}},\varOmega \right) ,1},\nonumber \\ \left\| {\textbf{f}}\right\| _{{\textbf{H}}\left( {\text {D}} ,\varOmega \right) ,\rho }&:=\left\| {\textbf{f}}\right\| _{{\textbf{H}} ^{0}\left( {\text {D}},\varOmega \right) ,\rho }=\left( \left\| {\text {D}}\,{\textbf{f}}\right\| ^{2}+\rho ^{2}\left\| {\textbf{f}} \right\| ^{2}\right) ^{1/2}, \end{aligned}$$

2.9 $$\begin{aligned} \left\| {\textbf{f}}\right\| _{{\textbf{H}}\left( {\text {D}} ,\varOmega \right) }&:=\left\| {\textbf{f}}\right\| _{{\textbf{H}} ^{0}\left( {\text {D}},\varOmega \right) }. \end{aligned}$$

We close this section with the introduction of the spaces of analytic functions:

#### Definition 2.1

For an open set $\omega \subset {\mathbb {R}}^{3}$, constants $C_{1}$, $\gamma _{1}>0$, and wavenumber $\left| k\right| \ge 1$, we set

$$\begin{aligned} {\mathcal {A}}(C_{1},\gamma _{1},\omega ):=\left\{ {{\textbf{u}}}\in (C^{\infty }(\omega ))^{3}\mid \left| {{\textbf{u}}}\right| _{{\textbf{H}}^{n}(\omega )}\le C_{1}\gamma _{1}^{n}\max \left\{ n+1,\left| k\right| \right\} ^{n}\;\forall n\in {\mathbb {N}}_{0}\right\} . \end{aligned}$$

### Sobolev Spaces on a Sufficiently Smooth Surface $\varGamma$

The Sobolev spaces on the boundary $\varGamma$ are denoted by $H^{s}( \varGamma )$ for scalar-valued functions and by ${\textbf{H}}^{s}( \varGamma )$ for vector-valued functions with norms $\left\| \cdot \right\| _{H^{s}( \varGamma ) }$, $\left\| \cdot \right\| _{{\textbf{H}}^{s}( \varGamma ) }$ (see, e.g., [30, p. 98]). Note that the range of *s* for which $H^{s}( \varGamma )$ is defined may be limited, depending on the global smoothness of the surface $\varGamma$; for Lipschitz surfaces, *s* can be chosen in the range $\left[ 0,1\right]$. For $s<0$, the space $H^{s}( \varGamma )$ is the dual of $H^{-s}( \varGamma )$.

Differential operators on $\varGamma$ are defined as described in [4, Sec. 2.5.6] using extensions to a three-dimensional neighborhood ${{\mathcal {U}}}$ of $\varGamma$: For a sufficiently smooth scalar field *u* on $\varGamma$, the constant extension along the normal direction is denoted $u^{*}$. For a sufficiently smooth tangential field $\textbf{v}$ the extension to ${{\mathcal {U}}}$ is formally given in [4, 2.5.188] and denoted ${{\textbf {v}}}^{*}$. One key feature of the extension ${{\textbf {v}}}^{*}$ is that it is tangential to surfaces parallel to $\varGamma$. The *surface gradient* $\nabla _{\varGamma }$, the *tangential curl* $\overrightarrow{\mathop {{\text {curl}}}\nolimits _{\varGamma }}$, and the *surface divergence* ${\text {div}}_{\varGamma }$ are defined by (cf., e.g., [6, 43])

$$\begin{aligned} \nabla _{\varGamma }u:=\left. \left( \nabla u^{\star }\right) \right| _{\varGamma }\text {,}\quad \overrightarrow{\mathop {{\text {curl}}}\nolimits _{\varGamma } }\,u:=\nabla _{\varGamma }u\times {\textbf{n}},\quad \text { and}\quad \mathop {{\text {div}}}\nolimits _{\varGamma }\,{\textbf{v}}=\left. \left( {\text {div}}\,{\textbf{v}}^{*}\right) \right| _{\varGamma }\qquad \text {on }\varGamma \text {.} \end{aligned}$$

The scalar counterpart of the tangential curl is the *surface curl*

$$\begin{aligned} \mathop {{\text {curl}}}\nolimits _{\varGamma }\,{\textbf{v}}:=\left\langle \left. \left( {\text {curl}}\,{\textbf{v}}^{*}\right) \right| _{\varGamma },{\textbf{n}}\right\rangle \qquad \text {on }\varGamma . \end{aligned}$$

The composition of the surface divergence and surface gradient leads to the *scalar Laplace-Beltrami operator* (see [43, (2.5.191)])

$$\begin{aligned} \varDelta _{\varGamma }u=\mathop {{\text {div}}}\nolimits _{\varGamma }\,\nabla _{\varGamma }u=-\mathop {{\text {curl}}}\nolimits _{\varGamma }\,\overrightarrow{\mathop {{\text {curl}}}\nolimits _{\varGamma }}\,u. \end{aligned}$$

From [43, (2.5.197)] it follows that

$$\begin{aligned} \mathop {{\text {div}}}\nolimits _{\varGamma }\,\left( {\textbf{v}}\times {\textbf{n}} \right) =\mathop {{\text {curl}}}\nolimits _{\varGamma }\,{\textbf{v}}. \end{aligned}$$

Next, we introduce Hilbert spaces of tangential fields on the compact and *simply connected* manifold $\varGamma$ and corresponding norms and refer for their definitions and properties to [43, Sec. 5.4.1]. We start with the definition of the space ${\textbf{L}}_{T}^{2}(\varGamma )$ of tangential vector fields given by

2.10 $$\begin{aligned} {\textbf{L}}_{T}^{2}(\varGamma ):=\left\{ {\textbf{v}}\in {\textbf{L}}^{2}(\varGamma )\mid \left\langle {\textbf{n}},{\textbf{v}}\right\rangle =0\text { on } \varGamma \right\} . \end{aligned}$$

Any tangential field ${\textbf{v}}_{T}$ on $\varGamma$ then can be represented in terms of the *Hodge decomposition*^2^as

2.11 $$\begin{aligned} {\textbf{v}}_{T}={\textbf{v}}_{T}^{\nabla }+{\textbf{v}}_{T}^{{\text {curl}} }\quad \text {with}\quad {\textbf{v}}_{T}^{\nabla }:=\nabla _{\varGamma }V^{\nabla } \quad \text {and}\quad {\textbf{v}}_{T}^{{\text {curl}}}:=\overrightarrow{\mathop {{\text {curl}}}\nolimits _{\varGamma }}\,V^{{\text {curl}}} \end{aligned}$$

for some scalar potentials

$$\begin{aligned} V^{\nabla }\in H^{1}(\varGamma )\quad \text {and}\quad V^{{\text {curl}}}\in H(\overrightarrow{\mathop {{\text {curl}}}\nolimits _{\varGamma }},\varGamma ):=\left\{ \phi \in L^{2}(\varGamma )\mid \overrightarrow{\mathop {{\text {curl}}}\nolimits _{\varGamma }}\,\phi \in {\textbf{L}}_{T}^{2}(\varGamma )\right\} . \end{aligned}$$

In particular, this decomposition is ${\textbf{L}}_{T}^{2}$-orthogonal:

$$\begin{aligned} \left( {\textbf{v}}_{T}^{\nabla },{\textbf{v}}_{T}^{{\text {curl}}}\right) _{{\textbf{L}}^{2}(\varGamma )}=\left( \nabla _{\varGamma }V^{\nabla },\overrightarrow{\mathop {{\text {curl}}}\nolimits _{\varGamma }}\,V^{{\text {curl}}}\right) _{{\textbf{L}}^{2}(\varGamma )}=0\quad \forall \,{\textbf{v}}_{T} = {\textbf{v}}_{T}^{\nabla } + {\textbf{v}}_{T}^{{\text {curl}}} \text { as in }(\text {2.11}). \end{aligned}$$

Hence, the splitting (2.11) is stable:

$$\begin{aligned} \left\| {\textbf{v}}_{T}\right\| _{{\textbf{L}}^{2}(\varGamma )}&=\left( \left\| \nabla _{\varGamma }V^{\nabla }\right\| _{{\textbf{L}}^{2}(\varGamma )} ^{2}+\left\| \overrightarrow{\mathop {{\text {curl}}}\nolimits _{\varGamma } }\,V^{{\text {curl}}}\right\| _{{\textbf{L}}^{2}(\varGamma )}^{2}\right) ^{1/2},\\ \left\| \nabla _{\varGamma }V^{\nabla }\right\| _{{\textbf{L}}^{2}(\varGamma )}&\le \left\| {\textbf{v}}_{T}\right\| _{{\textbf{L}}^{2}\left( \varGamma \right) }\quad \text {and}\quad \left\| \overrightarrow{\mathop {{\text {curl}}}\nolimits _{\varGamma }}\,V^{{\text {curl}}}\right\| _{{\textbf{L}}^{2}(\varGamma )}\le \left\| {\textbf{v}}_{T}\right\| _{{\textbf{L}}^{2}(\varGamma )}. \end{aligned}$$

Higher order spaces are defined for $s>0$ by

2.12 $$\begin{aligned} {\textbf{H}}_{T}^{s}(\varGamma ):=\left\{ {\textbf{v}}_{T}\in {\textbf{L}}_{T} ^{2}(\varGamma )\mid \left\| {\textbf{v}}_{T}\right\| _{{\textbf{H}}^{s}( \varGamma ) }<\infty \right\} \end{aligned}$$

and for negative *s* by duality.

The $H^{s}(\varGamma )$-norm of $\mathop {{\text {curl}}}\nolimits _{\varGamma }\,\left( \cdot \right)$ and ${\text {div}}_{\varGamma }\left( \cdot \right)$ can be expressed by using the Hodge decomposition:

$$\begin{aligned} \left\| \mathop {{\text {curl}}}\nolimits _{\varGamma }\,{\textbf{v}}_{T}\right\| _{H^{s}(\varGamma )}&=\left\| \mathop {{\text {curl}}}\nolimits _{\varGamma }\,{\textbf{v}}_{T}^{{\text {curl}}}\right\| _{H^{s}(\varGamma )}\!=\!\left\| \mathop {{\text {curl}}}\nolimits _{\varGamma }\,\overrightarrow{\mathop {{\text {curl}}}\nolimits _{\varGamma }}\,V^{{\text {curl}}}\right\| _{H^{s}(\varGamma )}\!\!=\!\left\| \varDelta _{\varGamma }V^{{\text {curl}}}\right\| _{H^{s}(\varGamma )}, \\ \left\| \mathop {{\text {div}}}\nolimits _{\varGamma }\,{\textbf{v}}_{T}\right\| _{H^{s}(\varGamma )}&=\left\| \mathop {{\text {div}}}\nolimits _{\varGamma }\,{\textbf{v}}_{T}^{\nabla }\right\| _{H^{s}(\varGamma )}=\left\| \mathop {{\text {div}}}\nolimits _{\varGamma }\,\nabla _{\varGamma }V^{\nabla }\right\| _{H^{s}(\varGamma )}=\left\| \varDelta _{\varGamma }V^{\nabla }\right\| _{H^{s}(\varGamma )}. \end{aligned}$$

We define

2.13a $$\begin{aligned} \left\| {\textbf{v}}_{T}\right\| _{{\textbf{H}}^{-1/2}( \mathop {{\text {curl}}}\nolimits _{\varGamma },\varGamma ) }&:=\left( \left\| \mathop {{\text {curl}}}\nolimits _{\varGamma }\,{\textbf{v}}_{T} ^{{\text {curl}}}\right\| _{H^{-1/2}( \varGamma ) } ^{2}+\left\| {\textbf{v}}_{T}\right\| _{{\textbf{H}}^{-1/2}( \varGamma ) }^{2}\right) ^{1/2}\nonumber \\&=\left( \left\| \varDelta _{\varGamma }V^{{\text {curl}}}\right\| _{H^{-1/2}( \varGamma ) } ^{2}+\left\| {\textbf{v}}_{T}\right\| _{{\textbf{H}}^{-1/2}( \varGamma ) }^{2}\right) ^{1/2}, \end{aligned}$$

2.13b $$\begin{aligned} \left\| {\textbf{v}}_{T}\right\| _{{\textbf{H}}^{-1/2}( {\text {div}}_{\varGamma },\varGamma ) }&:=\left( \left\| \mathop {{\text {div}}}\nolimits _{\varGamma }\,{\textbf{v}}_{T}^{\nabla }\right\| _{H^{-1/2}( \varGamma ) }^{2}+\left\| {\textbf{v}}_{T}\right\| _{{\textbf{H}}^{-1/2}( \varGamma ) }^{2}\right) ^{1/2} \nonumber \\&=\left( \left\| \varDelta _{\varGamma }V^{\nabla }\right\| _{H^{-1/2}\left( \varGamma \right) }^{2}+\left\| {\textbf{v}}_{T}\right\| _{{\textbf{H}} ^{-1/2}( \varGamma ) }^{2}\right) ^{1/2}. \end{aligned}$$

The corresponding spaces ${\textbf{H}}_{T}^{-1/2}(\mathop {{\text {curl}}}\nolimits _{\varGamma },\varGamma )$ and ${\textbf{H}}_{T}^{-1/2}(\mathop {{\text {div}}}\nolimits _{\varGamma },\varGamma )$ are characterized by

2.14 $$\begin{aligned} \begin{array}{ccc} {\textbf{v}}_{T}\in {\textbf{H}}_{T}^{-1/2}(\mathop {{\text {div}}}\nolimits _{\varGamma },\varGamma ) &{} \iff &{} {\textbf{v}}_{T}\text { has form }(\text {2.11})\text { and }\left\| {\textbf{v}}_{T}\right\| _{{\textbf{H}}^{-1/2}\left( {\text {div}}_{\varGamma },\varGamma \right) }<\infty ,\\ {\textbf{v}}_{T}\in {\textbf{H}}_{T}^{-1/2}(\mathop {{\text {curl}}}\nolimits _{\varGamma },\varGamma ) &{} \iff &{} {\textbf{v}}_{T}\text { has form }(\text {2.11})\text { and }\left\| {\textbf{v}}_{T}\right\| _{{\textbf{H}}^{-1/2}\left( \mathop {{\text {curl}}}\nolimits _{\varGamma },\varGamma \right) }<\infty . \end{array} \nonumber \\ \end{aligned}$$

We also introduce indexed norms for functions in Sobolev spaces on the boundary: for $\nu \in {\mathbb {R}}$ with $2\nu \in {\mathbb {N}}_{0}$, we formally set

2.15a $$\begin{aligned} \left\| {\textbf{g}}_{T}\right\| _{{\textbf{H}}^{\nu }(\varGamma ),k}&:=\left( \sum \limits _{\ell =0}^{2\nu } \left| k\right| ^{1-\ell }\left\| {\textbf{g}}_{T}\right\| _{{\textbf{H}}^{\ell /2}(\varGamma )}^{2}\right) ^{1/2}\quad \text {and} \end{aligned}$$

2.15b $$\begin{aligned} \left\| {\textbf{g}}_{T}\right\| _{{\textbf{H}}^{-\nu }(\varGamma ),k}&:=\left| k\right| ^{\nu +1/2}\left\| {\textbf{g}}_{T}\right\| _{{\textbf{H}}^{-\nu }(\varGamma )}. \end{aligned}$$

For ${\text {D}}_{\varGamma }\in \left\{ \mathop {{\text {curl}}}\nolimits _{\varGamma },{\text {div}}_{\varGamma }\right\}$, we introduce^3^

2.16 $$\begin{aligned} \left\| {\textbf{g}}_{T}\right\| _{{\textbf{H}}^{\nu }({\text {D}} _{\varGamma },\varGamma ),k}:=\left( \left\| \mathop {{\text {D}}}\nolimits _{\varGamma }\,{\textbf{g}}_{T}\right\| _{{\textbf{H}}^{\nu }(\varGamma ),k}^{2}+\left| k\right| ^{2}\left\| {\textbf{g}}_{T}\right\| _{{\textbf{H}}^{\nu }(\varGamma ),k}^{2}\right) ^{1/2}. \end{aligned}$$

In particular, we have

$$\begin{aligned} \left\| {\textbf{g}}_{T}\right\| _{{\textbf{H}}^{0}( \varGamma ),k}=\left| k\right| ^{1/2}\left\| {\textbf{g}}_{T}\right\| _{{\textbf{L}}^2(\varGamma )}\quad \text {and}\quad \left\| {\textbf{g}}_{T}\right\| _{{\textbf{H}}^{\nu }\left( \varGamma \right) }\le C\left| k\right| ^{-1/2+\nu }\left\| {\textbf{g}}_{T}\right\| _{{\textbf{H}}^{\nu }\left( \varGamma \right) ,k}. \end{aligned}$$

We remark that the special dual norms $\Vert \cdot \Vert _{{\textbf{H}} ^{-1/2}({\text {div}}_{\varGamma },\varGamma ),k}$ and $\Vert \cdot \Vert _{{\textbf{X}}_{{\text {imp}}}^{\prime }( \varGamma ),k}$ on the boundary will be defined later in (4.4) and in (4.5). By using standard interpolation inequalities for Sobolev spaces we obtain the following lemma.

#### Lemma 2.2

For $m\in {\mathbb {N}}_{0}$, there holds

2.17 $$\begin{aligned} \left\| {\textbf{g}}_{T}\right\| _{{\textbf{H}}^{m+1/2}(\varGamma ),k}&\le C\left( \left| k\right| \left\| {\textbf{g}}_{T}\right\| _{{\textbf{L}}^{2}(\varGamma )}^{2}+ \sum \limits _{r=1}^{m+1} \left| k\right| ^{2-2r}\left\| {\textbf{g}}_{T}\right\| _{{\textbf{H}}^{r-1/2}(\varGamma )}^{2}\right) ^{1/2} \nonumber \\&\le C\left( \displaystyle \sum \limits _{r=0}^{m+1} \left| k\right| ^{2-2r}\left\| {\textbf{g}}_{T}\right\| _{{\textbf{H}}^{r-1/2}(\varGamma )}^{2}\right) ^{1/2}, \nonumber \\ \left\| {\textbf{g}}_{T}\right\| _{{\textbf{H}}^{m+1/2}(\varGamma ),k}&\le C\left( \left| k\right| \left\| {\textbf{g}}_{T}\right\| _{{\textbf{L}}^{2}(\varGamma )}^{2}+\left| k\right| ^{-2m}\left\| {\textbf{g}}_{T}\right\| _{{\textbf{H}}^{m+1/2}(\varGamma )}^{2}\right) ^{1/2} \nonumber \\&\le C\left( \left| k\right| ^{2}\left\| {\textbf{g}} _{T}\right\| _{{\textbf{H}}^{-1/2}(\varGamma )}^{2}+\left| k\right| ^{-2m}\left\| {\textbf{g}}_{T}\right\| _{{\textbf{H}}^{m+1/2}(\varGamma )} ^{2}\right) ^{1/2}. \end{aligned}$$

### Trace Operators and Energy Spaces for Maxwell’s Equations

We introduce tangential trace operators $\varPi _{T}$ and $\gamma _{T}$, which map sufficiently smooth functions ${\textbf{u}}$ in $\overline{\varOmega }$ to tangential fields on $\varGamma$, by

2.18 $$\begin{aligned} \varPi _{T}:{\textbf{u}}\mapsto {\textbf{n}}\times \left( {\textbf{u}}|_{\varGamma } \times {\textbf{n}}\right) ,\quad \gamma _{T}:{\textbf{u}}\mapsto {\textbf{u}} |_{\varGamma }\times {\textbf{n}}. \end{aligned}$$

The following theorem shows that ${\textbf{H}}_{T}^{-1/2}(\mathop {{\text {div}}}\nolimits _{\varGamma },\varGamma )$ and ${\textbf{H}}_{T}^{-1/2}(\mathop {{\text {curl}}}\nolimits _{\varGamma },\varGamma )$ are the correct spaces for the continuous extension of the tangential trace operators to Hilbert spaces.

#### Proposition 2.3

([7, 43, Thm. 5.4.2]) The trace mappings $\varPi _{T}$ and $\gamma _{T}$ in (2.18) extend to continuous and surjective operators

$$\begin{aligned} \varPi _{T}:{\textbf{X}}\rightarrow {\textbf{H}}_{T}^{-1/2}(\mathop {{\text {curl}}}\nolimits _{\varGamma },\varGamma ),\qquad \gamma _{T}:{\textbf{X}}\rightarrow {\textbf{H}}_{T}^{-1/2}(\mathop {{\text {div}}}\nolimits _{\varGamma },\varGamma ). \end{aligned}$$

Moreover, for theses trace spaces there exist continuous divergence-free liftings ${\mathcal {E}}_{{\text {curl}}}^{\varGamma }:{\textbf{H}}_{T} ^{-1/2}( \mathop {{\text {curl}}}\nolimits _{\varGamma },\varGamma ) \rightarrow {\textbf{X}}$ and ${\mathcal {E}}_{{\text {div}}}^{\varGamma }:{\textbf{H}}_{T}^{-1/2}( \mathop {{\text {div}}}\nolimits _{\varGamma },\varGamma ) \rightarrow {\textbf{X}}$.

For a vector field ${\textbf{u}}\in {\textbf{X}}$, we will employ frequently the notation

$$\begin{aligned} {\textbf{u}}_{T}:=\varPi _{T}{\textbf{u}}. \end{aligned}$$

From [43, (2.5.161), (2.5.208)] and the relation $\varPi _{T}\nabla u\mathbf {=n\times }\left( \left. \nabla u\right| _{\varGamma }\times {\textbf{n}}\right) =\left. \nabla u\right| _{\varGamma }-\left( \partial _{{\textbf{n}}}u\right) {\textbf{n}}$ we conclude

2.19 $$\begin{aligned} \varPi _{T}\nabla u&=\nabla _{\varGamma }( u\vert _{\varGamma }), \end{aligned}$$

2.20 $$\begin{aligned} \gamma _{T}\nabla u&=( \varPi _{T}\nabla u) \times {\textbf{n}} =\nabla _{\varGamma }( u\vert _{\varGamma }) \times {\textbf{n}}. \end{aligned}$$

#### Remark 2.4

For gradient fields $\nabla \varphi$ we have $(\nabla \varphi )_{T}^{{\text {curl}}}=0$ and $(\nabla \varphi )_{T}^{\nabla }=\nabla _{\varGamma }\varphi$. $\square$

#### Definition 2.5

Let $\varOmega \subset {\mathbb {R}}^{3}$ be a bounded domain with sufficiently smooth Lipschitz boundary $\varGamma$ as described in Sect. 2.1. The *energy space* for Maxwell’s equations with *impedance boundary conditions* on $\varGamma$ and real wavenumber $k\in {\mathbb {R}}\backslash \left( -k_{0},k_{0}\right)$ is

2.21 $$\begin{aligned} {\textbf{X}}_{{\text {imp}}}:=\left\{ {\textbf{u}}\in {\textbf{X}}:\varPi _{T}\mathbf {u\in L}_{T}^{2}(\varGamma )\right\} \end{aligned}$$

with corresponding norm

$$\begin{aligned} \left\| {\textbf{u}}\right\| _{{\text {imp}},k}:=\left[ \left\| {\text {curl}}\,{\textbf{u}}\right\| ^{2}+\left\| {\textbf{u}} \right\| _{k,+}^{2}\right] ^{1/2}\text { with }\left\| {\textbf{u}}\right\| _{k,+}:=\left[ k^{2}\left\| {\textbf{u}}\right\| ^{2}+\left| k\right| \left\| {\textbf{u}}_{T}\right\| _{{\textbf{L}}^{2}(\varGamma )}^{2}\right] ^{1/2}. \end{aligned}$$

Its *companion space* of scalar potentials is

2.22 $$\begin{aligned} H_{{\text {imp}}}^{1}(\varOmega ):=\left\{ \varphi \in H^{1}(\varOmega )\mid \left. \varphi \right| _{\varGamma }\in H^{1}(\varGamma )\right\} . \end{aligned}$$

### Regular Decompositions

We will rely on various decompositions of functions into regular parts and gradient parts. The decompositions may not be orthogonal but must be stable. We refer to [20, §4.4] and the bibliographic notes therein for some early contributions. Many variants have been introduced since then, and the results in this section are essentially taken from the literature: Lemma 2.6 is a consequence of [15, Thm. 4.6]; Lemma 2.7 relies on [41, Thm. 3.38] and Lemma 2.6; Lemma 2.8 is based on [13] while closely related results can be found in [3]. Finally, Lemma 2.9 is a consequence of [50, Thm. 4.2(2)] and [13]. For newer overview articles, we refer to, e.g., [21, 24]. The following Lemma 2.6 collects a key result from the seminal paper [15]. The operator ${\textbf{R}}_{2}$, which is essentially a right inverse of the curl operator, will frequently be employed in the present paper.

#### Lemma 2.6

Let $\varOmega$ be a bounded Lipschitz domain. There exist pseudodifferential operators $R_{1}$, ${\textbf{R}}_{2}$ of order $-1$ and ${\textbf{K}}$, ${\textbf{K}}_{2}$ of order $-\infty$ on ${{\mathbb {R}}}^{3}$ with the following properties: For each $m\in {{\mathbb {Z}}}$ they have the mapping properties $R_{1}:{\textbf{H}}^{-m}(\varOmega )\rightarrow H^{1-m}( \varOmega )$, ${\textbf{R}}_{2}:{\textbf{H}}^{-m} (\varOmega )\rightarrow {\textbf{H}}^{1-m}( \varOmega )$, and ${\textbf{K}}$, ${\textbf{K}}_{2}:{\textbf{H}}^{m}( \varOmega ) \rightarrow ({C}^{\infty }(\overline{\varOmega }))^{3}$, and for any ${\textbf{u}}\in {\textbf{H}}^{m}( {\text {curl}},\varOmega )$ there holds

2.23 $$\begin{aligned} {\textbf{u}}=\nabla R_{1}( {\textbf{u}}-{\textbf{R}}_{2}( {\text {curl}}\,{\textbf{u}}) ) +{\textbf{R}}_{2}( {\text {curl}}\,{\textbf{u}}) +\textbf{Ku}. \end{aligned}$$

For ${\textbf{u}}$ with ${\text {div}}\,{\textbf{u}}=0$ on $\varOmega$ there holds

2.24 $$\begin{aligned} {\text {curl}}\,{\textbf{R}}_{2}{\textbf{u}}={\textbf{u}}-{\textbf{K}} _{2}{\textbf{u}}. \end{aligned}$$

#### Proof

In [15, Thm. 4.6], operators $R_{1}$, ${\textbf{R}}_{2}$, ${\textbf{R}}_{3}$, ${\textbf{K}}_{1}$, ${\textbf{K}}_{2}$ with the mapping properties

2.25 $$\begin{aligned}{}\begin{array}{cl} R_{1}:{\textbf{H}}^{-m}(\varOmega )\rightarrow H^{1-m}\left( \varOmega \right) , &{} \\ {\textbf{R}}_{2}:{\textbf{H}}^{-m}(\varOmega )\rightarrow {\textbf{H}}^{1-m}\left( \varOmega \right) , &{} \\ {\textbf{R}}_{3}:H^{-m}(\varOmega )\rightarrow {\textbf{H}}^{1-m}\left( \varOmega \right) , &{} \\ {\textbf{K}}_{\ell }:{\textbf{H}}^{m}\left( \varOmega \right) \rightarrow ({C} ^{\infty }(\overline{\varOmega }))^{3}, &{} \ell =1,2, \end{array} \end{aligned}$$

are constructed with

2.26a $$\begin{aligned} \nabla R_{1}{\textbf{v}}+{\textbf{R}}_{2}({\text {curl}}\,{\textbf{v}})&={\textbf{v}}-{\textbf{K}}_{1}{\textbf{v}}, \end{aligned}$$

2.26b $$\begin{aligned} {\text {curl}}\,{\textbf{R}}_{2}{\textbf{v}}+{\textbf{R}}_{3}\left( {\text {div}}\,{\textbf{v}}\right)&={\textbf{v}}-{\textbf{K}}_{2} {\textbf{v}}. \end{aligned}$$

We note that (2.26) implies (2.24). It is worth stressing that the mapping properties given in (2.25) express a locality of the operators, which are pseudodifferential operators on ${\mathbb {R}}^{3}$: on $\varOmega$, the operators depend only on the argument restricted to $\varOmega$ and not on the values on ${\mathbb {R}}^{3}\setminus \overline{\varOmega }$.

Selecting ${\textbf{v}}={\textbf{u}}-{\textbf{R}}_{2}({\text {curl}}\,{\textbf{u}})$ in (2.26a) we obtain

2.27 $$\begin{aligned} \begin{aligned}&\nabla R_{1}({\textbf{u}}-{\textbf{R}}_{2}({\text {curl}}\,{\textbf{u}} ))+{\textbf{R}}_{2}({\text {curl}}({\textbf{u}}-{\textbf{R}}_{2} ({\text {curl}}\,{\textbf{u}})))\\&\quad = {\textbf{u}}-{\textbf{R}}_{2} ({\text {curl}}\,{\textbf{u}})-{\textbf{K}}_{1}({\textbf{u}}-{\textbf{R}} _{2}({\text {curl}}\,{\textbf{u}})). \end{aligned} \end{aligned}$$

Since ${\text {curl}}\,{\textbf{u}}$ is divergence free, we obtain from (2.26b)

$$\begin{aligned} {\textbf{R}}_{2}({\text {curl}}({\textbf{u}}-{\textbf{R}}_{2}\left( {\text {curl}}\,{\textbf{u}}))\right)&={\textbf{R}}_{2}\left( {\text {curl}}\,{\textbf{u}}\right) -{\textbf{R}}_{2}({\text {curl}}\,{\textbf{u}}-{\textbf{K}}_{2}{\text {curl}}\,{\textbf{u}})\\&={\textbf{R}}_{2}({\textbf{K}}_{2}({\text {curl}}\,{\textbf{u}} ))=:{\textbf{K}}_{3}{\textbf{u}}, \end{aligned}$$

where, again, ${\textbf{K}}_{3}$ is a smoothing operator of order $-\infty$. Inserting this into (2.27) leads to

$$\begin{aligned} \nabla R_{1}\left( {\textbf{u}}-{\textbf{R}}_{2}\left( {\text {curl}}\,{\textbf{u}}\right) \right) +{\textbf{R}}_{2}\left( {\text {curl}}\,{\textbf{u}}\right) ={\textbf{u}}-{\textbf{K}}_{1}\left( {\textbf{u}}-{\textbf{R}} _{2}\left( {\text {curl}}\,{\textbf{u}}\right) \right) -{\textbf{K}} _{3}{\textbf{u}}. \end{aligned}$$

By choosing ${\textbf{K}}{\textbf{u}}:={\textbf{K}}_{1}\left( {\textbf{u}} -{\textbf{R}}_{2}{\text {curl}}\,{\textbf{u}}\right) +{\textbf{K}} _{3}{\textbf{u}}$ the representation (2.23) is proved. $\square$

#### Lemma 2.7

Let $\varOmega$ be a bounded, connected Lipschitz domain.

(i) There is $C>0$ such that for every ${\textbf{u}}\in {\textbf{X}}$ there is a decomposition ${\textbf{u}} =\nabla \varphi +{\textbf{z}}$ with
  2.28 $$\begin{aligned} {\text {div}}\,{\textbf{z}}=0,\quad \left\| {\textbf{z}}\right\| _{{\textbf{H}}^{1}(\varOmega )}\le C\left\| {\text {curl}}\,{\textbf{u}} \right\| ,\quad \left\| \varphi \right\| _{H^{1}(\varOmega )}\le C\left\| {\textbf{u}}\right\| _{{\textbf{H}}\left( {\text {curl}},\varOmega \right) }. \nonumber \\ \end{aligned}$$
(ii) Let $m\in {\mathbb {Z}}$. For each ${\textbf{u}}\in {\textbf{H}}^{m}( {\text {curl}},\varOmega )$ there is a splitting independent of *m* of the form ${\textbf{u}}=\nabla \varphi +{\textbf{z}}$ with $\varphi \in H^{m+1}( \varOmega )$, ${\textbf{z}}\in {\textbf{H}}^{m+1}( \varOmega )$ satisfying
  2.29a $$\begin{aligned} \left\| {\textbf{z}}\right\| _{{\textbf{H}}^{m+1}(\varOmega )}&\le C\left\| {\textbf{u}}\right\| _{{\textbf{H}}^{m}( {\text {curl}} ,\varOmega ) }\quad \text {and}\quad \end{aligned}$$
  2.29b $$\begin{aligned} \left\| {\textbf{z}}\right\| _{{\textbf{H}}^{m}(\varOmega )}+\left\| \varphi \right\| _{H^{m+1}(\varOmega )}&\le C\left\| {\textbf{u}}\right\| _{{\textbf{H}}^{m}( \varOmega ) }. \end{aligned}$$
(iii) There is $C > 0$ depending only on $\varOmega$ such that each ${\textbf{u}} \in {\textbf{X}} _{{\text {imp}}}$ can be written as ${\textbf{u}} = \nabla \varphi + {\textbf{z}}$ with $\varphi \in H^{1}_{{\text {imp}}}(\varOmega )$, ${\textbf{z}} \in {\textbf{H}}^{1}(\varOmega )$ and
  2.30 $$\begin{aligned} \Vert \nabla \varphi \Vert _{{\text {imp}},k} + \Vert {\textbf{z}}\Vert _{{\textbf{H}} ^{1}(\varOmega )} + |k| \Vert {\textbf{z}}\Vert _{{\textbf{L}}^{2}(\varOmega )} + |k|^{1/2} \Vert {\textbf{z}}\Vert _{{\textbf{L}}^{2}(\varGamma )} \le C \Vert {\textbf{u}} \Vert _{{\text {imp}},k}. \end{aligned}$$

#### Proof

*Proof of* (i): Let ${\textbf{u}}\in {\textbf{X}}$. The point is to choose ${\textbf{z}}$ in the splitting ${\textbf{u}}=\nabla \varphi +{\textbf{z}}$ such that it can be controlled by ${\text {curl}}\,{\textbf{u}}$. To this end, we set ${\textbf{v}} ={\text {curl}}\,{\textbf{u}}\in {\textbf{L}}^{2}(\varOmega )$ and observe that ${\text {div}}\,{\textbf{v}}=0$ and therefore $(1,\langle {{\textbf{n}} },{{\textbf{v}}}\rangle )_{L^{2}(\varGamma )}=0$. By [41, Thm. 3.38], this allows us to conclude the existence of ${\textbf{z}}\in {\textbf{H}} ^{1}(\varOmega )$ with ${\text {div}}\,{\textbf{z}}=0$, ${\textbf{v}} ={\text {curl}}\,{\textbf{z}}$ and

$$\begin{aligned} \left\| {\textbf{z}}\right\| _{{\textbf{H}}^{1}(\varOmega )}\le C\left\| {\textbf{v}}\right\| . \end{aligned}$$

Since ${\text {curl}}({\textbf{u}}-{\textbf{z}})=0$, we have ${\textbf{u}} -{\textbf{z}}=\nabla \varphi$ for a $\varphi \in H^{1}(\varOmega )$, which trivially satisfies

$$\begin{aligned} \left( \nabla \varphi ,\nabla \psi \right) =\left( {\textbf{u}}-{\textbf{z}},\nabla \psi \right) \qquad \forall \psi \in H^{1}(\varOmega ). \end{aligned}$$

By fixing $\varphi$ such that $\int _{\varOmega }\varphi =0$, the estimate of $\varphi$ follows by a Poincaré inequality.

*Proof of* (ii): With the operators of Lemma 2.6, we define

$$\begin{aligned} {\textbf{z}}:={\textbf{R}}_{2}({\text {curl}}\,{\textbf{u}})+{\textbf{K}} {\textbf{u}},\qquad \nabla \varphi :=\nabla R_{1}({\textbf{u}}-{\textbf{R}} _{2}({\text {curl}}\,{\textbf{u}})). \end{aligned}$$

Lemma 2.6 implies ${\textbf{u}}={\textbf{z}}+\nabla \varphi$ as well as the bounds by the mapping properties given in Lemma 2.6.

*Proof of* (iii): Multiplying estimate (2.29b) for the decomposition of (ii) and $m=0$ by $\left| k\right|$ leads to $\left| k\right| \Vert {\textbf{z}}\Vert _{{\textbf{L}}^{2}(\varOmega )}+\left| k\right| \Vert \nabla \varphi \Vert _{{\textbf{L}}^{2}(\varOmega )}\le C\left| k\right| \Vert {\textbf{u}}\Vert _{{\textbf{L}}^{2}(\varOmega )}$. (2.29a) gives $\Vert {\textbf{z}} \Vert _{{\textbf{H}}^{1}(\varOmega )}\le C\Vert {\textbf{u}}\Vert _{{\textbf{H}} ({\text {curl}},\varOmega )}$. The multiplicative trace inequality gives $|k|\Vert {\textbf{z}}\Vert _{{\textbf{L}}^{2}(\varGamma )}^{2}\le C\left| k\right| \Vert {\textbf{z}}\Vert _{{\textbf{L}}^{2}(\varOmega )}\Vert {\textbf{z}} \Vert _{{\textbf{H}}^{1}(\varOmega )}\le C\left| k\right| ^{2} \Vert {\textbf{z}}\Vert _{{\textbf{L}}^{2}(\varOmega )}^{2}+C\Vert {\textbf{z}} \Vert _{{\textbf{H}}^{1}(\varOmega )}^{2}$. Hence follows $\Vert {\textbf{z}}\Vert _{{\text {imp}},k}\le C\Vert {\textbf{u}}\Vert _{{\textbf{H}} ({\text {curl}},\varOmega ),k}\le C\Vert {\textbf{u}}\Vert _{{\text {imp}},k}$. The triangle inequality then provides the bound $\Vert \nabla \varphi \Vert _{{\text {imp}},k}\le \Vert {\textbf{u}}\Vert _{{\text {imp}},k}+\Vert {\textbf{z}}\Vert _{{\text {imp}},k}\le C\Vert {\textbf{u}} \Vert _{{\text {imp}},k}$. $\square$

The following result relates the space ${\textbf{H}}({\text {curl}},\varOmega )\cap {\textbf{H}}({\text {div}},\varOmega )$ to classical Sobolev spaces. The statement (2.32) is from [13]; closely related results can be found in [3].

#### Lemma 2.8

Let $\partial \varOmega$ be smooth and simply connected. Then there is $C>0$ such that for every ${\textbf{u}}\in {\textbf{H}}\left( {\text {curl}},\varOmega \right) \cap {\textbf{H}}\left( {\text {div}},\varOmega \right)$ there holds

2.31a $$\begin{aligned} \left\| {\textbf{u}}\right\|&\le C\left( \left\| {\text {curl}}\,{\textbf{u}}\right\| +\left\| {\text {div}}\,{\textbf{u}}\right\| +\left\| \left\langle {\textbf{u}},{\textbf{n}} \right\rangle \right\| _{H^{-1/2}( \varGamma ) }\right) , \end{aligned}$$

2.31b $$\begin{aligned} \left\| {\textbf{u}}\right\|&\le C\left( \left\| {\text {curl}}\,{\textbf{u}}\right\| +\left\| {\text {div}}\,{\textbf{u}}\right\| +\left\| \gamma _{T}{\textbf{u}}\right\| _{{\textbf{H}}^{-1/2}( \varGamma ) }\right) . \end{aligned}$$

Under the assumption^4^ that $\left\langle {\textbf{u}},{\textbf{n}}\right\rangle \in L^{2}( \varGamma )$ or $\gamma _{T}\mathbf {u\in L}_{T}^{2}( \varGamma )$, there holds

2.32a $$\begin{aligned} \left\| {\textbf{u}}\right\| _{{\textbf{H}}^{1/2}(\varOmega )}&\le C\left( \left\| {\text {curl}}\,{\textbf{u}}\right\| +\left\| {\text {div}}\,{\textbf{u}}\right\| +\left\| \left\langle {\textbf{u}},{\textbf{n}}\right\rangle \right\| _{L^{2}\left( \varGamma \right) }\right) , \end{aligned}$$

2.32b $$\begin{aligned} \Vert {\textbf{u}}\Vert _{{\textbf{H}}^{1/2}(\varOmega )}&\le C\left( \left\| {\text {curl}}\,{\textbf{u}}\right\| +\left\| {\text {div}}\,{\textbf{u}}\right\| +\left\| \gamma _{T}{\textbf{u}}\right\| _{{\textbf{L}}^{2}\left( \varGamma \right) }\right) . \end{aligned}$$

#### Proof

We use the regular decomposition $\displaystyle {\textbf{u}}=\nabla \varphi +{\textbf{z}},$ of Lemma 2.7 (i) where ${\textbf{z}}\in {\textbf{H}}^{1}( \varOmega )$ satisfies

2.33 $$\begin{aligned} \left\| {\textbf{z}}\right\| _{{\textbf{H}}^{1}( \varOmega ) }\le C\left\| {\text {curl}}\,{\textbf{u}}\right\| . \end{aligned}$$

Since ${\text {div}}\,{\textbf{z}}=0$ we have $\displaystyle \varDelta \varphi ={\text {div}}\,{\textbf{u}}.$ Concerning the boundary conditions for $\varphi$, we consider two cases corresponding to (2.31a), (2.32a) and (2.31b), (2.32b) separately as Case 1 and Case 2.

*Case 1*: The function $\varphi$ satisfies the Neumann problem

$$\begin{aligned} \varDelta \varphi ={\text {div}}\,{\textbf{u}},\qquad \partial _{n}\varphi =\left\langle {\textbf{n}},\nabla \varphi \right\rangle =\left\langle {\textbf{n}},{\textbf{u}}-{\textbf{z}}\right\rangle , \end{aligned}$$

and we note that the condition ${\text {div}}\,{\textbf{z}}=0$ implies that the solvability condition for this Neumann problem is satisfied. We estimate

$$\begin{aligned} \left\| \left\langle {\textbf{n}},{\textbf{u}}-{\textbf{z}}\right\rangle \right\| _{H^{-1/2}( \varGamma ) }&\le \Vert \left\langle {\textbf{n}},{\textbf{u}}\right\rangle \Vert _{H^{-1/2}( \varGamma ) }+\left\| \left\langle {\textbf{n}},{\textbf{z}}\right\rangle \right\| _{H^{-1/2}( \varGamma ) } \\&\le \Vert \left\langle {\textbf{n}} ,{\textbf{u}}\right\rangle \Vert _{H^{-1/2}\left( \varGamma \right) }+ C \left\| {\textbf{z}}\right\| _{{\textbf{H}}^{1}(\varOmega )}. \end{aligned}$$

An energy estimate for $\varphi$ provides $\displaystyle \left\| \nabla \varphi \right\| \le C( \left\| {\text {div}}\,{\textbf{u}}\right\| +\left\| \partial _{n} \varphi \right\| _{H^{-1/2}\left( \varGamma \right) }).$ The combination of these estimates lead to (2.31a). We also note that if $\left\langle {\textbf{u}},{\textbf{n}}\right\rangle \in L^{2}( \varGamma )$, then we get by the smoothness of $\varGamma$ that $\varphi \in H^{3/2}(\varOmega )$ with $\Vert \varphi \Vert _{H^{3/2}(\varOmega )}\le C(\Vert {\text {div}}\,{{\textbf{u}}}\Vert +\Vert \partial _{n}\varphi \Vert _{L^{2}(\varGamma )})$, which shows (2.32a).

*Case 2*: We obtain regularity assertions for $\varphi$ by using that $\varphi$ satisfies $\varDelta \varphi = {\text {div}}\,{\textbf{u}}$ and determine the boundary regularity $\varphi |_\varGamma$. We observe

$$\begin{aligned} \overrightarrow{\mathop {{\text {curl}}}\nolimits _{\varGamma }}\,\varphi =\gamma _{T}\nabla \varphi =\gamma _{T}\left( {\textbf{u}}-{\textbf{z}}\right) \end{aligned}$$

and therefore

$$\begin{aligned} \varDelta _{\varGamma }\varphi =-{\text {curl}}_{\varGamma }\,\overrightarrow{{\text {curl}}_{\varGamma }}\varphi =-{\text {curl}}_{\varGamma }\left( \gamma _{T}\left( {\textbf{u}}-{\textbf{z}}\right) \right) . \end{aligned}$$

Hence, by smoothness of $\partial \varOmega$ (and the fact that $\partial \varOmega$ is connected) we get

$$\begin{aligned} \left\| \varphi \right\| _{H^{1/2}(\varGamma )}&\le C\left\| \varDelta _{\varGamma }\varphi \right\| _{H^{-3/2}(\varGamma )}=C\left\| {\text {curl}}_{\varGamma }\,\left( \gamma _{T}\left( {\textbf{u}} -{\textbf{z}}\right) \right) \right\| _{H^{-3/2}(\varGamma )} \\&\le C\left\| \gamma _{T}\left( {\textbf{u}}-{\textbf{z}}\right) \right\| _{{\textbf{H}} ^{-1/2}(\varGamma )} \le C\left( \left\| \gamma _{T}{\textbf{u}}\right\| _{{\textbf{H}} ^{-1/2}(\varGamma )}+\left\| {\textbf{z}}\right\| _{{\textbf{H}}^{1}(\varOmega )}\right) . \end{aligned}$$

Since $\Vert \varphi \Vert _{H^{1}(\varOmega )}\le C(\Vert {\text {div}}\,{\textbf{u}}\Vert +\Vert \varphi \Vert _{H^{1/2}(\varGamma )})$ we get (2.31b). By similar reasoning, $\gamma _{T}{\textbf{u}} \in {\textbf{L}}^{2}(\varGamma )$ implies $\varphi |_{ \varGamma }\in H^{1}( \varGamma )$ with $\Vert \varphi \Vert _{H^{1}(\varGamma )}\le C(\Vert \gamma _{T} {\textbf{u}}\Vert _{L^{2}(\varGamma )}+\Vert {\textbf{z}}\Vert _{{\textbf{H}}^{1}(\varOmega )})$ so that $\varphi \in H^{3/2}(\varOmega )$ and thus (2.32b). $\square$

The following lemma introduces some variants of Helmholtz decompositions.

#### Lemma 2.9

Let $\varOmega$ be a bounded sufficiently smooth Lipschitz domain with simply connected boundary. For any ${\textbf{u}} \in {\textbf{X}}_{{\text {imp}}}\cap {\textbf{H}}( {\text {div}},\varOmega )$, there exist $\varphi \in H_{0}^{1}(\varOmega ) \cap H^{3/2}( \varOmega )$ and ${\textbf{z}}\in {\textbf{H}}^{1}( \varOmega )$ with ${\text {div}}\,{\textbf{z}}=0$ such that ${\textbf{u}}=\nabla \varphi +{\text {curl}}\,{\textbf{z}}$. The function ${\textbf{u}}$ belongs to ${\textbf{H}}^{1/2}(\varOmega )$, and we have the estimates

2.34a $$\begin{aligned} \left\| \nabla \varphi \right\| _{{\textbf{H}}^{1/2}(\varOmega )}&\le C\left\| {\textbf{u}}\right\| _{{\textbf{H}}^{1/2}(\varOmega )}, \end{aligned}$$

2.34b $$\begin{aligned} \left\| {\text {curl}}\,{\textbf{z}}\right\| _{{\textbf{H}}^{1/2} (\varOmega )}&\le C\left( \left\| {\text {curl}}\,{\textbf{u}} \right\| +\left\| \gamma _{T}{\textbf{u}}\right\| _{{\textbf{L}} ^{2}(\varGamma )}\right) . \end{aligned}$$

If ${\textbf{u}}$ admits a decomposition of the form ${\textbf{u}}={\textbf{r}} +{\text {curl}}\,{\varvec{\psi }}$ with ${\textbf{r}}\in {\textbf{H}}^{1/2}( \varOmega )$, then the decomposition ${\textbf{u}} = \nabla \varphi + {\text {curl}}\,{\textbf{z}}$ satisfies

2.34c $$\begin{aligned} \left\| \nabla \varphi \right\| _{{\textbf{H}}^{1/2}(\varOmega )}\le C\left\| {\textbf{r}}\right\| _{{\textbf{H}}^{1/2}(\varOmega )}. \end{aligned}$$

#### Proof

The Helmholtz decomposition was considered in [50, Thm. 4.2(2)], [49, Thm. 28(i)]. Since ${\text {div}}{\text {curl}}=0$ and we require $\varphi \in H_{0}^{1}(\varOmega )$, we have

2.35 $$\begin{aligned} \varDelta \varphi ={\text {div}}\,{\textbf{u}}\quad \text {and}\quad \varphi |_{\partial \varOmega }=0. \end{aligned}$$

Lemma 2.8 implies for ${\textbf{u}} \in {\textbf{X}}_{{\text {imp}}}\cap {\textbf{H}}( {\text {div}},\varOmega )$ that ${\textbf{u}}\in {\textbf{H}}^{1/2}(\varOmega )$. A standard shift theorem for the Poisson equation leads to

2.36 $$\begin{aligned} \left\| \varphi \right\| _{H^{3/2}(\varOmega )}\le C\left\| {\text {div}}\,{{\textbf{u}}}\right\| _{H^{-1/2}(\varOmega )}\le C\left\| {{\textbf{u}}}\right\| _{{\textbf{H}}^{1/2}(\varOmega )}. \end{aligned}$$

Next, we estimate ${\textbf{z}}$. Note that $\varphi \in H_{0}^{1}(\varOmega )$ implies $\nabla _{\varGamma }\varphi =0$ so that also $\gamma _{T}\nabla \varphi =0$ on $\varGamma$. Lemma 2.8 then implies

$$\begin{aligned} \Vert {\text {curl}}\,{\textbf{z}}\Vert _{{\textbf{H}}^{1/2}(\varOmega )}&\le C\left( \left\| {\text {curl}}\,{\text {curl}}\,{\textbf{z}} \right\| +\left\| \gamma _{T}{\text {curl}}\,{\textbf{z}}\right\| _{{\textbf{L}}^{2}(\varGamma )}\right) \\&\le C\left( \left\| {\text {curl}}\,{\textbf{u}}\right\| +\left\| \gamma _{T}{\textbf{u}}\right\| _{{\textbf{L}}^{2}(\varGamma )}+\left\| \gamma _{T}\nabla \varphi \right\| _{{\textbf{L}}^{2}(\varGamma )}\right) \\&\overset{\gamma _{T}\nabla \varphi =0}{=}C\left( \left\| {\text {curl}}\,{\textbf{u}}\right\| +\left\| \gamma _{T} {\textbf{u}}\right\| _{{\textbf{L}}^{2}(\varGamma )}\right) . \end{aligned}$$

The estimate (2.34c) follows from (2.36) via ${\text {div}}\,{\textbf{u}}={\text {div}}\,{\textbf{r}}$. This finishes the proof of (2.34). $\square$

### Maxwell’s Equations with Impedance Boundary Conditions

We have introduced all basic ingredients to formulate the electric Maxwell equations for constant wavenumber $k\in {\mathbb {R}}\backslash \left( -k_{0},k_{0}\right)$ with impedance boundary conditions on $\varGamma$. We define the sesquilinear form $A_{k}:{\textbf{X}}_{{\text {imp}}}\times {\textbf{X}} _{{\text {imp}}}\rightarrow {\mathbb {C}}$ by

2.37 $$\begin{aligned} A_{k}({\textbf{u}},{\textbf{v}}):=\left( {\text {curl}}\,{\textbf{u,}} \,{\text {curl}}\,{\textbf{v}}\right) -k^{2}\left( \textbf{u,v}\right) -{\text {i}}\,k\left( {\textbf{u}}_{T},{\textbf{v}}_{T}\right) _{{\textbf{L}}^{2}( \varGamma ) }. \end{aligned}$$

The variational formulation is: Given an electric current density ${\textbf{j}}$ and boundary data ${\textbf{g}}_T$ with

2.38 $$\begin{aligned} {\textbf{j}}\in {\textbf{L}}^{2}(\varOmega ),\qquad {\textbf{g}}_{T}\in {\textbf{H}} _{T}^{-1/2}\left( \mathop {{\text {div}}}\nolimits _{\varGamma },\varGamma \right) \cap {\textbf{L}}_{T}^{2}(\varGamma ) \end{aligned}$$

find ${\textbf{E}}\in {\textbf{X}}_{{\text {imp}}}$ such that

2.39 $$\begin{aligned} A_{k}({\textbf{E}},{\textbf{v}})=\left( \textbf{j,v}\right) +\left( {\textbf{g}}_{T},{\textbf{v}}_{T}\right) _{{\textbf{L}}^{2}( \varGamma ) }\quad \forall {\textbf{v}}\in {\textbf{X}}_{{\text {imp}}}. \end{aligned}$$

Note that the assumptions (2.38) on the data are not the most general ones (see (4.1), (4.2) and (4.17) below) but they reduce technicalities in some places. By integration by parts it is easy to see that the classical strong form of this equation is given by

2.40 $$\begin{aligned} \begin{array}{ll} {\mathcal {L}}_{\varOmega ,k}{\textbf{E}}={\textbf{j}} &{} \text {in }\varOmega ,\\ {\mathcal {B}}_{ \varGamma ,k}{\textbf{E}}={\textbf{g}}_{T} &{} \text {on }\varGamma \end{array} \end{aligned}$$

with the volume and boundary differential operators ${\mathcal {L}}_{\varOmega ,k}$ and ${\mathcal {B}}_{\varOmega ,k}$, defined by

$$\begin{aligned} {\mathcal {L}}_{\varOmega ,k}{\textbf{v}}:={\text {curl}}\,{\text {curl}}\,{\textbf{v}}-k^{2}{\textbf{v}}\text { in }\varOmega \quad \text {and}\quad {\mathcal {B}}_{ \varGamma ,k}{\textbf{v}}:=\gamma _{T} {\text {curl}}\,{\textbf{v}} -{\text {i}}\,k\varPi _{T}{\textbf{v}}\text { on }\varGamma \mathbf {.} \end{aligned}$$

We denote by

2.41 $$\begin{aligned} {\mathcal {S}}_{\varOmega ,k}^{{\text {MW}}}:{\textbf{X}}_{{\text {imp}} }^{\prime }\rightarrow {\textbf{X}}_{{\text {imp}}} \end{aligned}$$

the solution operator that maps the linear functional ${\textbf{X}} _{{\text {imp}}}\ni {\textbf{v}}\mapsto \left( \textbf{j,v}\right) +\left( {\textbf{g}}_{T},{\textbf{v}}\right) _{{\textbf{L}}^{2}\left( \varGamma \right) }$ to the solution ${\textbf{E}}$ of (2.40) and whose existence follows from Proposition 3.1 below.

In our analysis, the sesquilinear form

2.42 $$\begin{aligned} { \left( \!\left( \hspace{-0.50003pt} {\textbf{u}},{\textbf{v}} \hspace{-1.00006pt}\right) \!\right) }_k:=k^{2}\left( {\textbf{u}},{\textbf{v}}\right) +{\text {i}}\, k\left( {\textbf{u}}_{T},{\textbf{v}}_{T}\right) _{{\textbf{L}}^{2}( \varGamma ) } \end{aligned}$$

will play an important role. We note

2.43 $$\begin{aligned} A_{k}( {\textbf{u}},{\textbf{v}})&=\left( {\text {curl}}\,{\textbf{u,}}\, {\text {curl}}\,{\textbf{v}}\right) - { \left( \!\left( \hspace{-0.50003pt}{\textbf{u}},{\textbf{v}}\hspace{-1.00006pt}\right) \!\right) }_k, \end{aligned}$$

2.44 $$\begin{aligned} A_{k}( {\textbf{u}},\nabla \varphi )&=- { \left( \!\left( \hspace{-0.50003pt} {\textbf{u}},\nabla \varphi \hspace{-1.00006pt}\right) \!\right) }_k \qquad \forall {\textbf{u}}\in {\textbf{X}}_{{\text {imp}}} ,\quad \forall \varphi \in H_{{\text {imp}}}^{1}( \varOmega ) , \end{aligned}$$

2.45 $$\begin{aligned} { \left( \!\left( \hspace{-0.50003pt} {\textbf{u}},{\textbf{v}} \hspace{-1.00006pt}\right) \!\right) }_k&=\overline{ { \left( \!\left( \hspace{-0.50003pt} {\textbf{v}},{\textbf{u}}\hspace{-1.00006pt}\right) \!\right) }_k} . \end{aligned}$$

## Stability Analysis of the Continuous Maxwell Problem

In this section we show that the model problem (2.39) is well-posed and that the norm of the solution operator is $O(|k|^{\theta })$ for suitable choices of norms and some $\theta \ge 0$.

### Well-Posedness

The continuity of the sesquilinear form $A_{k}( \cdot ,\cdot )$ is obvious: it holds

$$\begin{aligned} \left| A_{k}( {\textbf{u}},{\textbf{v}}) \right| \le C_{{\text {cont}}}\left\| {\textbf{u}}\right\| _{{\text {imp}},k}\left\| {\textbf{v}}\right\| _{{\text {imp}},k}\qquad \text {with}\qquad C_{{\text {cont}}}:=1\text {.} \end{aligned}$$

Well-posedness of the Maxwell problem with impedance condition is proved in [41, Thm. 4.17]. Here we recall the statement and give a sketch of the proof.

#### Proposition 3.1

Let $\varOmega \subset {\mathbb {R}}^{3}$ be a bounded Lipschitz domain with simply connected and sufficiently smooth boundary. Then there exists $\gamma _{k}>0$ such that

$$\begin{aligned} \gamma _{k}\le \inf _{{\textbf{u}}\in {\textbf{X}}_{{\text {imp}}} \backslash \left\{ {\textbf{0}} \right\} }\sup _{{\textbf{v}}\in {\textbf{X}}_{{\text {imp}}}\backslash \left\{ {\textbf{0}} \right\} }\frac{\left| A_{k}({\textbf{u}},{\textbf{v}})\right| }{\left\| {\textbf{u}}\right\| _{{\text {imp}},k}\left\| {\textbf{v}}\right\| _{{\text {imp}},k}}. \end{aligned}$$

#### Proof

*Step 1*: We show uniqueness. If $A_{k}({\textbf{u}},{\textbf{v}})=0$ for all ${\textbf{v}}\in {\textbf{X}}_{{\text {imp}}}$ then

$$\begin{aligned} 0={\text {Im}}\,A_{k}({\textbf{u}},{\textbf{u}})=-k\left\| {\textbf{u}} _{T}\right\| _{{\textbf{L}}^{2}\left( \varGamma \right) }^{2}. \end{aligned}$$

Hence, ${\textbf{u}}_{T}={\textbf{0}}$ on $\varGamma$ and the extension of ${\textbf{u}}$ by zero outside of $\varOmega$ (denoted $\widetilde{{\textbf{u}}}$) is in ${\textbf{H}}({\text {curl}},{\widetilde{\varOmega }})$ for any bounded domain ${\widetilde{\varOmega }}\subset {\mathbb {R}}^{3}$. This zero extension $\widetilde{{\textbf{u}}}$ solves the homogeneous Maxwell equations on ${\mathbb {R}}^{3}$. An application of the operator “${\text {div}}$” shows that ${\text {div}}\widetilde{{\textbf{u}}}=0$ and thus $\widetilde{{\textbf{u}}}\in {\textbf{H}}^{1}({\mathbb {R}}^{3})$. Using ${\text {curl}}\,{\text {curl}}=-\varDelta +\nabla {\text {div}}$ we see that each component of $\widetilde{{\textbf{u}}}$ solves the homogeneous Helmholtz equation. Since $\widetilde{{\textbf{u}}}$ vanishes outside $\varOmega$, the unique continuation principle asserts $\widetilde{{\textbf{u}}}=0$.

*Step 2*: From [19, Thm. 4.8] or the technique developed in [5] it follows that the operator induced by $A_{k}$ is a compact perturbation of an isomorphism and the Fredholm alternative shows well-posedness of the problem. $\square$

### Wavenumber-Explicit Stability Estimates

Proposition 3.1 does not give any insight how the (positive) inf-sup constant $\gamma _{k}$ depends on the wavenumber *k*. In this section, we introduce the stability constant $C_{{\text {stab}}}(k)$ and estimate its dependence on *k* under certain assumptions.

#### Definition 3.2

(stability constant $C_{{\text {stab}}}(k)$) Let $\varOmega \subset {\mathbb {R}}^{3}$ be a bounded Lipschitz domain with simply connected and sufficiently smooth boundary. The stability constant $C_{{\text {stab}}}(k)$ is any constant such that for each ${\textbf{j}}\in {\textbf{L}}^{2}(\varOmega )$ and ${\textbf{g}}_{T}\in {\textbf{L}}_{T}^{2}(\varGamma )$ the solution ${\textbf{E}} = {\mathcal {S}}_{\varOmega ,k}^{{\text {MW}}} ({{\textbf{j}}}, {{\textbf{g}}}_T)$ of (2.39) satisfies

3.1 $$\begin{aligned} \Vert {\textbf{E}}\Vert _{{\text {imp}},k} \le C_{{\text {stab}}}\left( k\right) \left( \left\| {\textbf{j}}\right\| _{{\textbf{L}}^{2}(\varOmega )}+\left\| {\textbf{g}} _{T}\right\| _{{\textbf{L}}^{2}(\varGamma )}\right) . \end{aligned}$$

The behavior of the constant $C_{{\text {stab}}}\left( k\right)$ with respect to the wavenumber typically depends on the geometry of the domain $\varOmega$. Our stability and convergence theory for conforming Galerkin finite element discretization as presented in Sect. 9 requires that this constant grow at most algebraically in *k*, i.e.,

3.2 $$\begin{aligned} \exists \theta \ge 0,C_{{\text {stab}}}>0\quad \text {such that } C_{{\text {stab}}}\left( k\right) \le C_{{\text {stab}}}|k|^{\theta }\qquad \forall k\in {\mathbb {R}} \backslash \left( -k_{0},k_{0}\right) . \end{aligned}$$

#### Remark 3.3

For the *hp*-FEM application below, the term $\left| k\right| ^{\theta }$ will be mitigated by an exponentially converging approximation term so that any finite value $\theta \ge 0$ leads to an exponential convergence of the discretization. $\square$

Next we present an example^5^ which shows that in general the exponent $\theta$ in (3.2) cannot be negative.

#### Example 3.4

Let $\varOmega =\left( -2,2\right) ^{3}$, $\omega :=\left( -1,1\right) ^{2}$. Define the cutoff function $\chi :\varOmega \rightarrow {\mathbb {C}}$ by

$$\begin{aligned} \chi \left( {\textbf{x}}\right) :=\left\{ \begin{array}{ll} \left( 1-x_{1}^{2}\right) ^{2}\left( 1-x_{2}^{2}\right) ^{2}\left( 1-x_{3}^{2}\right) ^{2} &{}\text {if} \quad {\textbf{x}}=\left( x_{j}\right) _{j=1}^{3} \in \omega ,\\ 0 &{} \text {otherwise.} \end{array} \right. \end{aligned}$$

Note that $\chi \in H_{0}^{2}\left( \varOmega \right)$. With ${\textbf{e}}_{j} \in {\mathbb {R}}^3$, $1\le j\le 3$, denoting the *j*-th canonical unit vector we define

$$\begin{aligned} {\textbf{j}}:=\mathop {{\text {e}}}\nolimits ^{{\text {i}}\,kx_{1}}\,\left( -\left( \varDelta \chi +{\text {i}}\,k\partial _{1}\chi \right) {\textbf{e}} _{2}+\nabla \partial _{2}\chi +{\text {i}}\,k\,{\text {curl}}\,\left( \chi {\textbf{e}}_{3}\right) \right) . \end{aligned}$$

Then, $\mathbf {E:}=\chi {\text {e}}^{{\text {i}}\,kx_{1}} {\textbf{e}}_{2}$ is the unique weak solution of

$$\begin{aligned} {\mathcal {L}}_{\varOmega ,k}{\textbf{E}}={\textbf{j}}&\text { in }\varOmega ,&{\mathcal {B}}_{\varGamma ,k}{\textbf{E}}={\textbf{0}}&\text { on }\varGamma . \end{aligned}$$

Using the symbolic computer algebra program MATHEMATICA we obtain

$$\begin{aligned} \left\| {\textbf{j}}\right\| _{{\textbf{L}}^{2}\left( \varOmega \right) } ^{2}&=\frac{16777216\left( 5k^{2}+33\right) }{10418625}, \\ \left\| {\textbf{E}}\right\| _{{\textbf{H}}({\text {curl}},\varOmega ),k}^{2}&=2\frac{16777216\left( k^{2}+3\right) }{31255875},\quad \left\| {\textbf{E}}_{T}\right\| _{{\textbf{L}}^{2}(\varGamma )}^{2}=0, \end{aligned}$$

which shows that in general $\theta \ge 0$ in (3.1). $\square$

#### Remark 3.5

Let $\varOmega \subset {\mathbb {R}}^{3}$ be a bounded Lipschitz domain with simply connected and sufficiently smooth boundary. The sesquilinear form $A_{k}$ satisfies the inf-sup condition

3.3 $$\begin{aligned} \inf _{{{{\textbf {u}}}}\in {{{\textbf {X}}}}_{{\text{ imp }}}\setminus \{0\}} \sup _{{{{\textbf {v}}}}\in {{{\textbf {X}}}}_{{\text{ imp }}}\setminus \{0\}} \frac{|A_{k}({\textbf{u}},{\textbf{v}})|}{\Vert {\textbf{u}}\Vert _{{\text {imp}},k}\Vert {\textbf{v}} \Vert _{{\text {imp}},k}}\ge \frac{1}{1+|k| C_{{\text {stab}}} \left( k\right) }. \end{aligned}$$

This result is shown in the same way as in the Helmholtz case, see, e.g., [17, Thm. 2.5], [32, Prop. 8.2.7]. If assumption (3.2) holds, then

$$\begin{aligned} \inf _{{{{\textbf {u}}}}\in {{{\textbf {X}}}}_{{\text{ imp }}}\setminus \{0\}} \sup _{{{{\textbf {v}}}}\in {{{\textbf {X}}}}_{{\text{ imp }}}\setminus \{0\}} \frac{|A_{k}({\textbf{u}},{\textbf{v}})|}{\Vert {\textbf{u}}\Vert _{{\text {imp}},k}\Vert {\textbf{v}} \Vert _{{\text {imp}},k}}\ge \frac{1}{1+C_{{\text {stab}}} |k|^{\theta +1}}. \end{aligned}$$

$$\square $$

In the remaining part of this section, we prove estimate (3.2) for certain classes of domains. The following result removes the assumption in [23] for the right-hand side to be solenoidal.

#### Proposition 3.6

Let $\varOmega \subset {\mathbb {R}}^{3}$ be a bounded $C^{2}$ domain that is star-shaped with respect to a ball. Then, Assumption (3.2) holds with $\theta =0$.

#### Proof

Let $$\varphi \in H_{0}^{1}(\varOmega )$$ satisfy

$$\begin{aligned} -\varDelta \varphi ={\text {div}}\,{\textbf{j}}\qquad \text{ in } \varOmega \text {.} \end{aligned}$$

Then,

$$\begin{aligned} \Vert k^{-2}\nabla \varphi \Vert _{{\text {imp}},k}&=|k|^{-1}\Vert \nabla \varphi \Vert _{{\textbf{L}}^{2}(\varOmega )}\le C|k|^{-1}\Vert {\text {div}}\,{\textbf{j}}\Vert _{H^{-1}(\varOmega )}\le C|k|^{-1} \Vert {\textbf{j}}\Vert _{{\textbf{L}}^{2}(\varOmega )},\\ \Vert {\textbf{j}}+\nabla \varphi \Vert _{{\textbf{L}}^{2}(\varOmega )}&\le \Vert {\textbf{j}}\Vert _{{\textbf{L}}^{2}(\varOmega )}+C\Vert {\text {div}}\,{\textbf{j}}\Vert _{H^{-1}(\varOmega )}\le C\Vert {\textbf{j}}\Vert _{{\textbf{L}} ^{2}(\varOmega )}. \end{aligned}$$

Noting that $\varphi$ vanishes on $\varGamma$, the difference ${\mathcal {S}} _{\varOmega ,k}^{{\text {MW}}}({\textbf{j}},{\textbf{g}}_{T})-\left| k\right| ^{-2}\nabla \varphi$ satisfies

$$\begin{aligned} {\mathcal {L}}_{\varOmega ,k}\left( {\mathcal {S}}_{\varOmega ,k}^{{\text {MW}} }({\textbf{j}},{\textbf{g}}_{T})-\left| k\right| ^{-2}\nabla \varphi \right) ={\textbf{j}}+\nabla \varphi ,\quad {\mathcal {B}}_{ \varGamma ,k}\!\left( {\mathcal {S}}_{\varOmega ,k}^{{\text {MW}}}({\textbf{j}},{\textbf{g}}_{T})-\left| k\right| ^{-2}\nabla \varphi \right) ={\textbf{g}}_{T}, \end{aligned}$$

and ${\text {div}}({\textbf{j}}+\nabla \varphi )=0$. [23, Thm. 3.1] implies

$$\begin{aligned} \Vert {\mathcal {S}}_{\varOmega ,k}^{{\text {MW}}}({\textbf{j}},{\textbf{g}} _{T})-\left| k\right| ^{-2}\nabla \varphi \Vert _{{\text {imp}} ,k}&\le C\left( \Vert {\textbf{j}}+\nabla \varphi \Vert _{{\textbf{L}}^{2}(\varOmega )}+\Vert {\textbf{g}}_{T}\Vert _{{\textbf{L}}^{2}(\varGamma )}\right) \\&\le C\left( \left\| {\textbf{j}}\right\| _{{\textbf{L}}^{2}( \varGamma ) }+\Vert {\textbf{g}}_{T}\Vert _{{\textbf{L}}^{2}(\varGamma )}\right) . \end{aligned}$$

The estimate for ${\mathcal {S}}_{\varOmega ,k}^{{\text {MW}}}({\textbf{j}},{\textbf{g}}_{T})$ follows from a triangle inequality. $\square$

For the more general situation of domains that are not necessarily star-shaped we require some preliminaries. A bounded domain $\varOmega$ with smooth boundary admits, e.g., by [22, Cor. 4.1] a continuous extension operator ${\mathcal {E}}_{{\text {div}}}:{\textbf{H}}^{m}( {\text {div}},\varOmega ) \rightarrow {\textbf{H}}^{m}( {\text {div}},{\mathbb {R}}^{3})$ for any $m\in {\mathbb {N}}_{0}$. In particular this extension can be chosen such that for a ball $B_{R}$ of radius *R* with $\varOmega \subset B_{R}$ there holds

3.4 $$\begin{aligned} {\text {supp}}\,\left( {\mathcal {E}}_{{\text {div}}}{\textbf{h}} \right) \subset B_{R}\quad \forall \,{\textbf{h}}\in {\textbf{H}} ({\text {div}},\varOmega ). \end{aligned}$$

Since the right-hand side ${\textbf{j}}$ in (2.40), in general, does not belong to ${\textbf{H}}({\text {div}},\varOmega )$ we subtract an appropriate gradient field: Let $\psi \in H_{0}^{1}(\varOmega )$ be the weak solution of $-\varDelta \psi ={\text {div}}\,{\textbf{j}}$. As in the proof of Proposition 3.6, we write ${\mathcal {S}}_{\varOmega ,k}^{{\text {MW}}}({\textbf{j}},{\textbf{g}}_{T})={\mathcal {S}}_{\varOmega ,k}^{{\text {MW}}}(\widetilde{{\textbf{j}}},{\textbf{g}}_{T})-k^{-2} \nabla \psi$ with

3.5 $$\begin{aligned} \widetilde{{\textbf{j}}}:={\textbf{j}}+\nabla \psi \in {\textbf{H}}({\text {div}},\varOmega ). \end{aligned}$$

The operator ${\mathcal {E}}_{{\text {div}}}$ allows us to extend $\widetilde{{\textbf{j}}}$ to a compactly supported function ${\textbf{J}}:={\mathcal {E}}_{{\text {div}}}(\widetilde{{\textbf{j}}}) \in {\textbf{H}}({\text {div}},{\mathbb {R}}^{3})$. Next we introduce the solution operator for the full space problem

3.6 $$\begin{aligned}{}\begin{array}{cl} {\text {curl}}\,{\text {curl}}\,{\textbf{Z}}-k^{2}{\textbf{Z}}={\textbf{J}} &{} \text {in }{\mathbb {R}}^{3},\\ \left| \partial _{r}{\textbf{Z}}\left( {\textbf{x}}\right) -{\text {i}}\, k{\textbf{Z}}\left( {\textbf{x}}\right) \right| \le c/r^{2} &{} \text {as }r=\left\| {\textbf{x}}\right\| \rightarrow \infty , \end{array} \end{aligned}$$

via the Maxwell potential

3.7a $$\begin{aligned} {\textbf{Z}}={\mathcal {N}}_{{\text {MW}},k}\left( {\textbf{J}}\right) :={\mathcal {N}}_{{\text {MW}},k}^{\nabla }\left( {\textbf{J}}\right) +{\mathcal {N}}_{{\text {MW}},k}^{{\text {curl}}}\left( {\textbf{J}}\right) , \end{aligned}$$

where^6^

3.7b $$\begin{aligned} \left. \begin{array}{l} {\mathcal {N}}_{{\text {MW}},k}^{{\text {curl}}}\left( {\textbf{J}}\right) := {\displaystyle \int _{{\mathbb {R}}^{3}}} g_{k}\left( \left\| \mathbf {\cdot }-{\textbf{y}}\right\| \right) {\textbf{J}}\left( {\textbf{y}}\right) d{\textbf{y}}\\ \\ {\mathcal {N}}_{{\text {MW}},k}^{\nabla }\left( {\textbf{J}}\right) :=k^{-2}\nabla {\mathcal {N}}_{{\text {MW}},k}^{{\text {curl}}}\left( {\text {div}}\,{\textbf{J}}\right) \end{array} \right\} \quad \text {in }{\mathbb {R}}^{3} \end{aligned}$$

with the fundamental solution of the Helmholtz equation in ${\mathbb {R}}^{3}$

3.7c $$\begin{aligned} g_{k}\left( r\right) :=\frac{\mathop {{\text {e}}}\nolimits ^{{\text {i}}\, kr}}{4\pi r}\text {.} \end{aligned}$$

Note that the adjoint full space problem is given by replacing *k* in (3.6) by $-k$ with solution operator ${\mathcal {N}} _{{\text {MW}},-k}\left( {\textbf{J}}\right)$.

The layer operators ${\mathcal {S}}_{{\text {MW}},k}^{\nabla }$, ${\mathcal {S}}_{{\text {MW}},k}^{{\text {curl}}}$ map densities defined on the boundary $\varGamma$ to functions defined in $\varOmega$ by

3.8 $$\begin{aligned} \left. \begin{array}{l} {\mathcal {S}}_{{\text {MW}},k}^{{\text {curl}}}\left( {\varvec{\mu }} \right) := {\displaystyle \int _{\varGamma }} g_{k}\left( \left\| \mathbf {\cdot }-{\textbf{y}}\right\| \right) {\varvec{\mu }} \left( {\textbf{y}}\right) d \varGamma _{{\textbf{y}}}\\ \\ {\mathcal {S}}_{{\text {MW}},k}^{\nabla }\left( {\varvec{\mu }} \right) :=k^{-2}\nabla {\mathcal {S}}_{{\text {MW}},k} ^{{\text {curl}}}\left( {\text {div}}_{\varGamma }\, {\varvec{\mu }} \right) \end{array} \right\} \quad \text {in }{\mathbb {R}}^{3}\backslash \varGamma . \end{aligned}$$

We set $${\mathcal {S}}_{{\mathbb {R}}^{3},k}^{{\text {MW}}}:={\mathcal {S}} _{{\text {MW}},k}^{\nabla }+{\mathcal {S}}_{{\text {MW}},k}^{{\text {curl}}}$$.

#### Theorem 3.7

Let $\varOmega \subset {\mathbb {R}}^{3}$ be a bounded Lipschitz domain with simply connected, analytic boundary. Then, there is $C > 0$ depending only on $\varOmega$ such that

$$\begin{aligned} C_{{\text {stab}}}\left( k\right) \le C \left| k\right| ^{7/2}\sqrt{1+\ln \left| k\right| }. \end{aligned}$$

#### Remark 3.8

The analyticity requirement of $\partial \varOmega$ can be relaxed. It is due to our citing [34], which assumes analyticity. $\square$

#### Proof

We estimate ${\mathcal {S}}_{\varOmega ,k}^{{\text {MW}}}({\textbf{j}},{\textbf{g}}_{T})$ (see (2.41)) for given $({\textbf{j}},{\textbf{g}}_{T})\in {\textbf{L}}^{2}(\varOmega )\times {\textbf{L}}_{T}^{2}(\varGamma )$.

*Step 1* *(reduction to solenoidal right-hand side)*: Let $\psi$, $\widetilde{{\textbf{j}}}$ be as in (3.5) so that ${\mathcal {S}}_{\varOmega ,k}^{{\text {MW}}}({\textbf{j}},{\textbf{g}} _{T})={\mathcal {S}}_{\varOmega ,k}^{{\text {MW}}}(\widetilde{{\textbf{j}} },{\textbf{g}}_{T})-k^{-2}\nabla \psi$ and ${\text {div}}\widetilde{{\textbf{j}}}=0$. As in the proof of Proposition 3.6, we have

3.9 $$\begin{aligned} \Vert k^{-2}\nabla \psi \Vert _{{\text {imp}},k}\le C \left| k\right| ^{-1} \Vert {\textbf{j}}\Vert _{{\textbf{L}}^{2}(\varOmega )}, \quad \Vert \widetilde{{\textbf{j}}} \Vert _{{\textbf{L}}^{2}(\varOmega )}\le C\Vert {\textbf{j}}\Vert _{{\textbf{L}} ^{2}(\varOmega )}, \quad {\text {div}}\widetilde{{\textbf{j}}} =0.\nonumber \\ \end{aligned}$$

In particular,

$$\begin{aligned} \Vert \widetilde{{\textbf{j}}}\Vert _{{\textbf{H}}({\text {div}},\varOmega )}\le C\Vert {\textbf{j}}\Vert _{{\textbf{L}}^{2}(\varOmega )}. \end{aligned}$$

*Step 2* *(reduction to homogeneous volume right-hand side)*: We set

3.10 $$\begin{aligned} {\tilde{{\textbf{g}}}}_{T}:={\textbf{g}}_{T}-{\mathcal {B}}_{ \varGamma ,k}{\textbf{u}}_{{\textbf{j}}}\quad \text {with}\quad {\textbf{u}}_{{\textbf{j}} }:=\left. \left( {\mathcal {N}}_{{\text {MW}},k}{\mathcal {E}} _{{\text {div}}}{\tilde{{\textbf{j}}}}\right) \right| _{\varOmega } \end{aligned}$$

so that ${\mathcal {S}}_{\varOmega ,k}^{{\text {MW}}}(\widetilde{{\textbf{j}} },{\tilde{{\textbf{g}}}}_{T}) ={\textbf{u}}_{0}+{\textbf{u}}_{{\textbf{j}}}$ with ${\textbf{u}}_{0}$ being the solution of the homogeneous problem

3.11 $$\begin{aligned} \begin{array}{ll} {\text {curl}}\,{\text {curl}}\,{\textbf{u}}_{0}-k^{2}{\textbf{u}} _{0}={\textbf{0}} &{} \text {in }\varOmega ,\\ \gamma _{T}\left( {\text {curl}}\,{\textbf{u}}_{0}\right) -{\text {i}}\,k\left( {\textbf{u}}_{0}\right) _{T}={\tilde{{\textbf{g}}}}_{T} &{} \text {on }\varGamma . \end{array} \end{aligned}$$

To estimate ${\textbf{u}}_{{\textbf{j}}}$, we rely on the following estimate from [37, Lem. 3.5]

3.12 $$\begin{aligned}{} & {} |k|\Vert {\mathcal {N}}_{{\text {MW}},k}^{{\text {curl}}}(f)\Vert _{L^{2}(\varOmega )}+\Vert {\mathcal {N}}_{{\text {MW}},k}^{{\text {curl}} }(f)\Vert _{H^{1}(\varOmega )}\nonumber \\{} & {} \quad +|k|^{-1}\Vert {\mathcal {N}}_{{\text {MW}},k}^{{\text {curl}}}(f)\Vert _{H^{2}(\varOmega )}\le C\Vert f\Vert _{L^{2}({\mathbb {R}}^{3})} \end{aligned}$$

for all $f\in L^{2}({\mathbb {R}}^3)$. Abbreviate ${\textbf{N}}^{{\text {curl}}}:={\mathcal {N}}_{{\text {MW}},k}^{{\text {curl}}}({\mathcal {E}}_{{\text {div}}}\widetilde{{\textbf{j}}})$ and $N^{\nabla }:={\mathcal {N}}_{{\text {MW}},k} ^{{\text {curl}}}({\text {div}}\,{\mathcal {E}}_{{\text {div}} }\widetilde{{\textbf{j}}})$. Estimates (3.12) and (3.9) imply

$$\begin{aligned}&|k|\Vert {\textbf{N}}^{{\text {curl}}}\Vert _{{\textbf{L}}^{2}(\varOmega )} +\Vert {\textbf{N}}^{{\text {curl}}}\Vert _{{\textbf{H}}^{1}(\varOmega )} +|k|^{-1}\Vert {\textbf{N}}^{{\text {curl}}}\Vert _{{\textbf{H}}^{2}(\varOmega )} \le C\Vert {\textbf{j}}\Vert _{{\textbf{L}}^{2}(\varOmega )},\\&\quad |k|\Vert N^{\nabla }\Vert _{L^{2}(\varOmega )}+\Vert N^{\nabla }\Vert _{H^{1}(\varOmega )}+|k|^{-1}\Vert N^{\nabla }\Vert _{H^{2}(\varOmega )} \\&\quad \le C\Vert {\text {div}}\,{\mathcal {E}}_{{\text {div}}}{\tilde{{\textbf{j}}}} \Vert _{L^{2}({\mathbb {R}}^{3})}\le C\Vert {\textbf{j}}\Vert _{{\textbf{L}}^{2}(\varOmega )}. \end{aligned}$$

For ${\textbf{u}}_{{\textbf{j}}}={\textbf{N}}^{{\text {curl}}}+k^{-2}\nabla N^{\nabla }$ we get by a multiplicative trace inequality:

3.13 $$\begin{aligned} \Vert {\textbf{u}}_{{\textbf{j}}}\Vert _{{\text {imp}},k}&\le C\bigl (|k|\Vert {\textbf{N}}^{{\text {curl}}}\Vert _{{\textbf{L}}^{2}(\varOmega )}+\Vert {\textbf{N}}^{{\text {curl}}}\Vert _{{\textbf{H}}^{1}(\varOmega )}+|k|^{1/2}\Vert {\textbf{N}}^{{\text {curl}}}\Vert _{{\textbf{L}}^{2} (\varOmega )}^{1/2}\Vert {\textbf{N}}^{{\text {curl}}}\Vert _{{\textbf{H}} ^{1}(\varOmega )}^{1/2}\nonumber \\&\quad +|k|^{-1}\Vert {N}^{\nabla }\Vert _{{H}^{1}(\varOmega )} +|k|^{-3/2}\Vert \nabla _{\varGamma }{N}^{\nabla }\Vert _{{\textbf{L}}^{2}(\varGamma )}\bigr )\nonumber \\&\le C\left( \Vert {\textbf{j}}\Vert _{{\textbf{L}}^{2}(\varOmega )}+|k|^{-1} \Vert {\textbf{j}}\Vert _{{\textbf{L}}^{2}(\varOmega )}+|k|^{-3/2}\Vert N^{\nabla } \Vert _{H^{1}(\varOmega )}^{1/2}\Vert N^{\nabla }\Vert _{H^{2}(\varOmega )}^{1/2}\right) \nonumber \\&\le C\Vert {\textbf{j}}\Vert _{{\textbf{L}}^{2}(\varOmega )} . \end{aligned}$$

Arguing similarly, we get for $\widetilde{{\textbf{g}}}_{T}={\textbf{g}} _{T}-{\mathcal {B}}_{ \varGamma ,k}{\textbf{u}}_{{\textbf{j}}}$

3.14 $$\begin{aligned} \Vert \widetilde{{\textbf{g}}}_{T}\Vert _{{\textbf{L}}^{2}(\varGamma )}&\le \Vert {{\textbf{g}}}_{T}\Vert _{{\textbf{L}}^{2}(\varGamma )}+\Vert {\mathcal {B}}_{\varGamma ,k}{\textbf{u}}_{{\textbf{j}}}\Vert _{{\textbf{L}}^{2}(\varGamma )} \nonumber \\&\le C\left( \Vert {\textbf{g}}_{T}\Vert _{{\textbf{L}}^{2}(\varGamma )}+|k|^{1/2}\Vert {\textbf{j}}\Vert _{{\textbf{L}}^{2}(\varOmega )}\right) . \end{aligned}$$

*Step 3* *(Estimate of* $\gamma _{T}{\text {curl}}\,{\textbf{u}}_{0}$, $\gamma _{T}{\textbf{u}}_{0})$: To estimate the function ${\textbf{u}}_{0}$, we employ the Stratton-Chu formula (see, e.g., [12, Thm. 6.2], [43, (5.5.3)–(5.5.6)])

$$\begin{aligned} {\textbf{u}}_{0}={\text {curl}}\,{\mathcal {S}}_{{\text {MW}},k}^{{\text {curl}}}\left( \gamma _{T}{\textbf{u}}_{0}\right) +{\mathcal {S}}_{{{\mathbb {R}}}^{3},k}^{{\text {MW}}}\left( \gamma _{T}{\text {curl}}\,{\textbf{u}}_{0}\right) \quad \text {in }\varOmega . \end{aligned}$$

The weak formulation (2.39) implies

$$\begin{aligned} \left\| {\text {curl}}\,{\textbf{u}}_{0}\right\| ^{2}-k^{2}\left\| {\textbf{u}}_{0}\right\| ^{2}-{\text {i}}\,k\left\| \left( {\textbf{u}}_{0}\right) _{T}\right\| _{{\textbf{L}}^{2}(\varGamma )}^{2}=\left( {\tilde{{\textbf{g}}}}_{T},\left( {\textbf{u}}_{0}\right) _{T}\right) _{{\textbf{L}}^{2}(\varGamma )} \end{aligned}$$

from which we obtain by considering the imaginary part

3.15 $$\begin{aligned} \left| k\right| \left\| \left( {\textbf{u}}_{0}\right) _{T}\right\| _{{\textbf{L}}^{2}\left( \varGamma \right) }\le \left\| {\tilde{{\textbf{g}}}}_{T}\right\| _{{\textbf{L}}^{2}(\varGamma )}. \end{aligned}$$

From the real part, we then obtain by a Cauchy-Schwarz inequality

3.16 $$\begin{aligned} \left| \Vert {\text {curl}}\,{\textbf{u}}_{0}\Vert ^{2}-\left| k\right| ^{2}\Vert {\textbf{u}}_{0}\Vert ^{2}\right| \le C\left| k\right| ^{-1}\Vert {\tilde{{\textbf{g}}}}_{T}\Vert _{{\textbf{L}}^{2}(\varGamma )}^{2}. \end{aligned}$$

Next, we estimate the traces $\gamma _{T}{\text {curl}}\,{\textbf{u}}_{0}$ and $\gamma _{T}{\textbf{u}}_{0}$. Since $\varPi _{T}{\textbf{u}}_{0}\in {\textbf{L}} _{T}^{2}(\varGamma )$ we may employ $\gamma _{T}{\textbf{u}}_{0}=\left( \varPi _{T}{\textbf{u}}_{0}\right) \times {\textbf{n}}$ and (3.15) to obtain

3.17 $$\begin{aligned} \Vert \gamma _{T}{\textbf{u}}_{0}\Vert _{{\textbf{L}}^{2}(\varGamma )}=\left\| \varPi _{T}{\textbf{u}}_{0}\right\| _{{\textbf{L}}^{2}( \varGamma ) } \le \frac{1}{\left| k\right| }\Vert {\tilde{{\textbf{g}}}}_{T} \Vert _{{\textbf{L}}^{2}(\varGamma )}. \end{aligned}$$

The boundary conditions (second equation in (3.11)) lead to

3.18 $$\begin{aligned} \left\| \gamma _{T}\,{\text {curl}}\,{\textbf{u}}_{0}\right\| _{{\textbf{L}}^{2}( \varGamma ) }\le \left\| {\tilde{{\textbf{g}}}} _{T}\right\| _{{\textbf{L}}^{2}\left( \varGamma \right) }+\left| k\right| \left\| \left( {\textbf{u}}_{0}\right) _{T}\right\| _{{\textbf{L}}^{2}( \varGamma ) }\le 2\left\| {\tilde{{\textbf{g}}}} _{T}\right\| _{{\textbf{L}}^{2}( \varGamma ) }. \end{aligned}$$

The estimate (3.18) also implies

3.19 $$\begin{aligned} \left\| \mathop {{\text {div}}}\nolimits _{\varGamma }\,\gamma _{T}\,{\text {curl}}\,{\textbf{u}}_{0}\right\| _{{\textbf{H}}^{-1}( \varGamma ) } \le \left\| \gamma _{T}\,{\text {curl}}\,{\textbf{u}}_{0}\right\| _{{\textbf{L}}^{2}( \varGamma ) }\le 2\left\| {\tilde{{\textbf{g}}}} _{T}\right\| _{{\textbf{L}}^{2}( \varGamma ) }. \end{aligned}$$

*Step 4 (Mapping properties of Maxwell Layer Potentials)*:

The mapping properties of ${\text {curl}}\,{\mathcal {S}}_{{\text {MW}},k}^{{\text {curl}}}$, ${\mathcal {S}}_{{\text {MW}},k} ^{{\text {curl}}}$, and ${\mathcal {S}}_{{\text {MW}},k}^{\nabla }$ are well understood due to their relation with the acoustic single layer potential. We conclude from [34, Lem. 3.4, Thm. 5.3]:

3.20 $$\begin{aligned} \left\| {\mathcal {S}}_{{\text {MW}},k}^{{\text {curl}}} {\varvec{\mu }} \right\| _{{\textbf{H}}^{s}( \varOmega ) }&\le C_{s}\left| k\right| ^{s+1}\left\| {\varvec{\mu }} \right\| _{{\textbf{H}}^{s-3/2}( \varGamma ) }\quad \text {for } s\ge 0. \nonumber \\ \left\| {\textbf{u}}_{0}\right\| _{{\textbf{H}}^{-1/2}(\varOmega )}&\le \left\| {\text {curl}}\,{\mathcal {S}}_{{\text {MW}} ,k}^{{\text {curl}}}(\gamma _{T}{\textbf{u}}_{0})\right\| _{{\textbf{H}} ^{-1/2}(\varOmega )}\nonumber \\&\quad +\left\| {\mathcal {S}}_{{\text {MW}},k} ^{{\text {curl}}}(\gamma _{T}\,{\text {curl}}\,{\textbf{u}} _{0})\right\| _{{\textbf{H}}^{-1/2}(\varOmega )} +\left\| {\mathcal {S}}_{{\text {MW}},k}^{\nabla } (\gamma _{T}\,{\text {curl}}\,{\textbf{u}}_{0})\right\| _{{\textbf{H}} ^{-1/2}(\varOmega )} \nonumber \\&\le \left\| {\mathcal {S}}_{{\text {MW}},k} ^{{\text {curl}}}(\gamma _{T}{\textbf{u}}_{0})\right\| _{{\textbf{H}} ^{1/2}(\varOmega )}+\left\| {\mathcal {S}}_{{\text {MW}},k} ^{{\text {curl}}}(\gamma _{T}\,{\text {curl}}\,{\textbf{u}} _{0})\right\| \nonumber \\&\quad +\left| k\right| ^{-2}\left\| {\mathcal {S}} _{{\text {MW}},k}^{{\text {curl}}}(\mathop {{\text {div}}}\nolimits _{\varGamma }\,\gamma _{T}{\text {curl}}\,{\textbf{u}}_{0})\right\| _{{\textbf{H}}^{1/2}(\varOmega )}\nonumber \\&\le C\left( \left| k\right| ^{3/2}\left\| \gamma _{T}{\textbf{u}}_{0}\right\| _{{\textbf{L}}^{2}(\varGamma )}+C\left| k\right| \left\| \gamma _{T}{\text {curl}}\,{\textbf{u}} _{0}\right\| _{{\textbf{H}}^{-3/2}(\varGamma )}\right. \nonumber \\&\left. \quad +\left| k\right| ^{-1/2}\left\| \mathop {{\text {div}}}\nolimits _{\varGamma }\,\gamma _{T} \,{\text {curl}}\,{\textbf{u}}_{0}\right\| _{{\textbf{H}}^{-1}(\varGamma )}\right) . \end{aligned}$$

Inserting (3.17), (3.18), (3.19) in (3.20), we get

3.21 $$\begin{aligned} \left\| {\textbf{u}}_{0}\right\| _{{\textbf{H}}^{-1/2}\left( \varOmega \right) }\le C\left| k\right| \left\| {\tilde{{\textbf{g}}}}_{T}\right\| _{{\textbf{L}}^{2}(\varGamma )}. \end{aligned}$$

*Step 5*: Let ${\textbf{R}}_{2}$ and ${\textbf{K}}_{2}$ be as in Lemma 2.6 and consider

3.22 $$\begin{aligned} \widetilde{{\textbf{u}}}:={\textbf{r}}_{0}-{\text {curl}}\,{\textbf{u}}_{0} \quad \text {for}\quad {\textbf{r}}_{0}:={{\textbf{R}}}_{2}( {\text {curl}}\,{\text {curl}}\,{\textbf{u}}_{0}). \end{aligned}$$

Since ${\textbf{u}}_{0}\in {\textbf{X}}_{{\text {imp}}}$, we have ${\textbf{u}}_{0}\in {\textbf{L}}^{2}(\varOmega )$, and the relation ${\text {curl}}\,{\text {curl}}\,{\textbf{u}}_{0}-k^{2}{\textbf{u}} _{0}={\textbf{0}}$ implies ${\text {curl}}\,{\text {curl}}\,{\textbf{u}}_{0}\in {\textbf{L}}^{2}(\varOmega )$. Hence, ${{\textbf{r}}}_{0} \in {\textbf{H}}^{1}(\varOmega )$ together with the *k*-explicit bound

3.23 $$\begin{aligned} \left\| {{\textbf{r}}}_{0}\right\| _{{\textbf{H}}^{1/2}(\varOmega )}&=k^{2}\left\| {{\textbf{R}}}_{2}({\textbf{u}}_{0})\right\| _{{\textbf{H}} ^{1/2}(\varOmega )}\le C\left| k\right| ^{2}\left\| {\textbf{u}} _{0}\right\| _{{\textbf{H}}^{-1/2}(\varOmega )} \nonumber \\&\overset{(\text {3.21})}{\le }C\left| k\right| ^{3} \left\| {\tilde{{\textbf{g}}}}_{T}\right\| _{{\textbf{L}}^{2}(\varGamma )}. \end{aligned}$$

By the same reasoning and the mapping properties of ${{\textbf{R}}}_{2}$, we obtain

3.24 $$\begin{aligned} \left\| {{\textbf{r}}}_{0}\right\| _{{\textbf{H}}^{1}(\varOmega )}\le C\left| k\right| ^{2}\left\| {\textbf{u}}_{0}\right\| . \end{aligned}$$

Furthermore, we compute with Lemma 2.6

3.25 $$\begin{aligned} {\text {curl}}\,\widetilde{{\textbf{u}}}\overset{(\text {3.22})}{=}{\text {curl}}\,{\textbf{R}}_{2}({\text {curl}}\,{\text {curl}}\,{{\textbf{u}}}_{0})-{\text {curl}}\,{\text {curl}}\,{\textbf{u}}_{0} \overset{\text {Lem. }\text {2.6}}{=}-\,{\text {curl}}\,{{\textbf{K}}} _{2}{\text {curl}}\,{{\textbf{u}}}_{0}. \nonumber \\ \end{aligned}$$

We employ the Helmholtz decomposition of $\widetilde{{\textbf{u}}}$ in the form $\widetilde{{\textbf{u}}}=\nabla \varphi +{\text {curl}}\,{\textbf{z}}$ given in Lemma 2.9 with $\varphi \in H_{0}^{1}\left( \varOmega \right) \cap H^{3/2}( \varOmega )$, $\mathbf {z\in H}^{1}(\varOmega )$, and ${\text {div}}\,{\textbf{z}}=0$. Since ${\text {div}}\widetilde{{\textbf{u}}}={\text {div}}\,{\textbf{r}}_{0}$ the function $\varphi$ does not depend on ${\text {curl}}\,{\textbf{u}}_{0}$ (see (2.35)), and we obtain from (2.34a)

3.26 $$\begin{aligned} \left\| \varphi \right\| _{H^{3/2}(\varOmega )}\overset{(\text {2.34c})}{\le }C\left\| {{\textbf{r}}} _{0}\right\| _{{\textbf{H}}^{1/2}\left( \varOmega \right) }\overset{(\text {3.23})}{\le }C\left| k\right| ^{3} \left\| {\tilde{{\textbf{g}}}}_{T}\right\| _{{\textbf{L}}^{2}(\varGamma )}. \end{aligned}$$

Next, we estimate ${\textbf{z}}$. The definition of ${\textbf{r}}_{0}$ in (3.22) gives

3.27 $$\begin{aligned} \gamma _{T}\tilde{{\textbf{u}}}=\gamma _{T}{{\textbf{r}}}_{0}-\gamma _{T}\, {\text {curl}}\,{\textbf{u}}_{0}=\gamma _{T}{\textbf{r}}_{0}-{\tilde{{\textbf{g}}} }_{T}+{\text {i}}\,k({\textbf{u}}_{0})_{T}\in {\textbf{L}}_{T}^{2} (\varGamma ). \end{aligned}$$

Lemma 2.9 then implies

3.28 $$\begin{aligned}&\Vert {\text {curl}}\,{\textbf{z}}\Vert _{{\textbf{H}}^{1/2}( \varOmega ) } \le C\left( \left\| {\text {curl}}\, \widetilde{{\textbf{u}}}\right\| +\left\| \gamma _{T}\widetilde{{\textbf{u}} }\right\| _{{\textbf{L}}^{2}( \varGamma ) }\right) \nonumber \\&\quad \overset{(\text {3.25})}{=}C\left( \left\| {\text {curl}}\,{{\textbf{K}}}_{2}\left( {\text {curl}}\,{\textbf{u}} _{0}\right) \right\| +\left\| \gamma _{T}\widetilde{{\textbf{u}} }\right\| _{{\textbf{L}}^{2}( \varGamma ) }\right) \nonumber \\&\quad \overset{(\text {3.27}), \text {Lem. }\text {2.6}}{\le }C\left( \left\| {\textbf{u}}_{0}\right\| _{{\textbf{H}}^{-1/2}(\varOmega )}+\left\| \gamma _{T}{\textbf{r}}_{0}\right\| _{{\textbf{L}}^{2}( \varGamma ) }+\left| k\right| \left\| \left( {\textbf{u}} _{0}\right) _{T}\right\| _{{\textbf{L}}^{2}\left( \varGamma \right) }+\left\| {\tilde{{\textbf{g}}}}_{T}\right\| _{{\textbf{L}}^{2}( \varGamma ) }\right) \nonumber \\&\quad \overset{(\text {3.21}), (\text {3.15})}{\le }C\left( \left| k\right| \left\| {{\tilde{{\textbf{g}}}}}_{T}\right\| _{{\textbf{L}} ^{2}(\varGamma )}+\Vert \gamma _{T}{\textbf{r}}_{0}\Vert _{{\textbf{L}}^{2}( \varGamma ) }+\left\| {\tilde{{\textbf{g}}}}_{T}\right\| _{{\textbf{L}} ^{2}( \varGamma ) }\right) . \end{aligned}$$

*Step 6*: The combination of Step 5 with a trace inequality leads to

3.29 $$\begin{aligned} \left\| \widetilde{{\textbf{u}}}\right\| _{{\textbf{H}}^{1/2}( \varOmega ) }&\le \left\| \nabla \varphi \right\| _{{\textbf{H}} ^{1/2}( \varOmega ) }+\left\| {\text {curl}}\,{\textbf{z}} \right\| _{{\textbf{H}}^{1/2}( \varOmega ) } \nonumber \\&\overset{(\text {3.26}), (\text {3.28})}{\le }C\left( \left| k\right| ^{ 3 } \left\| {\tilde{{\textbf{g}}}}_{T}\right\| _{{\textbf{L}}^{2}\left( \varGamma \right) }+\left\| \gamma _{T}{\textbf{r}}_{0}\right\| _{{\textbf{L}} ^{2}( \varGamma ) }\right) . \end{aligned}$$

Let ${\textbf{B}}_{2,1}^{1/2}(\varOmega )$ denote the Besov space as defined, e.g., in [54]. Then the trace map $\displaystyle \gamma _{T}:{\textbf{B}}_{2,1}^{1/2}(\varOmega )\rightarrow {\textbf{L}}_{T}^{2}(\varGamma )$ is a continuous mapping (see [54, Thm. 2.9.3]), and we obtain from (3.29)

3.30 $$\begin{aligned} \left\| \widetilde{{\textbf{u}}}\right\| _{{\textbf{H}}^{1/2}( \varOmega ) }\le C\left( \left| k\right| ^{ 3 } \left\| {\tilde{{\textbf{g}}}}_{T}\right\| _{{\textbf{L}}^{2}( \varGamma ) }+\left\| {\textbf{r}}_{0}\right\| _{{\textbf{B}}_{2,1} ^{1/2}( \varOmega ) }\right) . \end{aligned}$$

This allows us to estimate

3.31 $$\begin{aligned} \left\| {\text {curl}}\,{\textbf{u}}_{0}\right\| _{{\textbf{H}} ^{1/2}( \varOmega ) }&\overset{(\text {3.22})}{\le }C\left( \left\| {\textbf{r}}_{0}\right\| _{{\textbf{H}}^{1/2}( \varOmega ) }+\left\| \widetilde{{\textbf{u}}}\right\| _{{\textbf{H}}^{1/2}( \varOmega ) }\right) \nonumber \\&\overset{(\text {3.23}), (\text {3.30})}{\le }C\left( \left| k\right| ^{ 3 } \Vert {\tilde{{\textbf{g}}}}_{T}\Vert _{{\textbf{L}}^{2}( \varGamma ) }+\left\| {\textbf{r}}_{0}\right\| _{{\textbf{B}}_{2,1}^{1/2}( \varOmega ) }\right) . \end{aligned}$$

To estimate $\left\| {\textbf{r}}_{0}\right\| _{{\textbf{B}}_{2,1} ^{1/2}\left( \varOmega \right) }$ we use the fact (see [54]) that the Besov space is an interpolation space $B_{2,1}^{1/2}( \varOmega ) =\left( L^{2}( \varOmega ),H^{1}( \varOmega ) \right) _{1/2,1}$ (via the so-called real method of interpolation). For $t\in (0,1]$ select $({\textbf{r}}_{0})_{t}\in {{\textbf{H}}}^{1}(\varOmega )$ as given by Lemma 3.9 and estimate with the interpolation inequality (by using the notation as in Lemma 3.9)

$$\begin{aligned}&\left\| {\textbf{r}}_{0}\right\| _{{\textbf{B}}_{2,1}^{1/2}( \varOmega ) } \le \left\| {\textbf{r}}_{0}-\left( {\textbf{r}} _{0}\right) _{t}\right\| _{{\textbf{B}}_{2,1}^{1/2}( \varOmega ) }+\left\| \left( {\textbf{r}}_{0}\right) _{t}\right\| _{{\textbf{B}} _{2,1}^{1/2}( \varOmega ) }\\&\quad \le C\left( \left\| {\textbf{r}}_{0}-\left( {\textbf{r}}_{0}\right) _{t}\right\| ^{1/2}\left\| {\textbf{r}}_{0}-\left( {\textbf{r}}_{0}\right) _{t}\right\| _{{\textbf{H}}^{1}\left( \varOmega \right) }^{1/2}+\left\| \left( {\textbf{r}}_{0}\right) _{t}\right\| _{{\textbf{B}}_{2,1}^{1/2}( \varOmega ) }\right) \\&\quad \overset{\text {Lem. }\text {3.9}}{\le }C\left( t^{1/4}\left\| {\textbf{r}}_{0}\right\| _{{\textbf{H}}^{1/2}( \varOmega ) }^{1/2}\left( \left\| {\textbf{r}}_{0}\right\| _{{\textbf{H}}^{1}( \varOmega ) }^{1/2}+t^{-1/4}\left\| {\textbf{r}}_{0}\right\| _{{\textbf{H}}^{1/2}( \varOmega ) } ^{1/2}\right) +\left\| \left( {\textbf{r}}_{0}\right) _{t}\right\| _{{\textbf{B}}_{2,1}^{1/2}( \varOmega ) }\right) \\&\quad \overset{(\text {3.34}), (\text {3.35})}{\le }C\left( \left\| {\textbf{r}}_{0}\right\| _{{\textbf{H}}^{1/2}( \varOmega ) }+t^{1/2}\Vert {\textbf{r}}_{0}\Vert _{{\textbf{H}}^{1}( \varOmega ) }+\sqrt{1+|\ln t|}\Vert {\textbf{r}}_{0}\Vert _{{\textbf{H}}^{1/2}( \varOmega ) }\right) \\&\quad \le C\left( t^{1/2}\left\| {\textbf{r}}_{0}\right\| _{{\textbf{H}} ^{1}( \varOmega ) }+\sqrt{1+\left| \ln t\right| }\left\| {\textbf{r}}_{0}\right\| _{{\textbf{H}}^{1/2}\left( \varOmega \right) }\right) \\&\quad \overset{(\text {3.23}), (\text {3.24})}{\le }C\left( t^{1/2}\left| k\right| ^{2}\left\| {\textbf{u}}_{0}\right\| +\sqrt{1+\left| \ln t\right| }\left| k\right| ^{2} \Vert {\textbf{u}}_{0}\Vert _{{\textbf{H}}^{-1/2}( \varOmega ) }\right) . \end{aligned}$$

Using (3.16) we get

$$\begin{aligned}&\left| k\right| \left\| {\textbf{u}}_{0}\right\| \le C\left( \left\| {\text {curl}}\,{\textbf{u}}_{0}\right\| ^{2}+\left| \left( \left| k\right| \left\| {\textbf{u}}_{0}\right\| \right) ^{2}-\left\| {\text {curl}}\,{\textbf{u}}_{0}\right\| ^{2}\right| \right) ^{1/2}\\&\quad \le C\left( \left| k\right| ^{-1/2}\left\| {\tilde{{\textbf{g}}}}_{T}\right\| _{{\textbf{L}}^{2}\left( \varGamma \right) }+\left\| {\text {curl}}\,{\textbf{u}}_{0}\right\| \right) \overset{(\text {3.31})}{\le }C\left( \left| k\right| ^{ 3 } \left\| {\tilde{{\textbf{g}}}}_{T}\right\| _{{\textbf{L}}^{2}\left( \varGamma \right) }+\left\| {\textbf{r}}_{0}\right\| _{{\textbf{B}}_{2,1} ^{1/2}\left( \varOmega \right) }\right) \\&\quad \le C\left( \left| k\right| ^{ 3 } \left\| {\tilde{{\textbf{g}}}}_{T}\right\| _{{\textbf{L}}^{2}\left( \varGamma \right) }+t^{1/2}\left| k\right| ^{2}\left\| {\textbf{u}} _{0}\right\| +\sqrt{1+\left| \ln t\right| }\left| k\right| ^{2}\left\| {\textbf{u}}_{0}\right\| _{{\textbf{H}}^{-1/2}\left( \varOmega \right) }\right) \\&\quad \overset{(\text {3.21})}{\le }C\left( \sqrt{1+\left| \ln t\right| }\left| k\right| ^{ 3 } \left\| {\tilde{{\textbf{g}}}}_{T}\right\| _{{\textbf{L}}^{2}\left( \varGamma \right) }+t^{1/2}\left| k\right| ^{2}\left\| {\textbf{u}} _{0}\right\| \right) . \end{aligned}$$

Selecting $t\sim 1/\left| k\right| ^{2}$ sufficiently small implies

$$\begin{aligned} \left| k\right| \left\| {\textbf{u}}_{0}\right\| \le C\left| k\right| ^{ 3 } \sqrt{1+\ln \left| k\right| }\left\| {\tilde{{\textbf{g}}}} _{T}\right\| _{{\textbf{L}}^{2}( \varGamma ) }. \end{aligned}$$

We conclude from this and (3.31)

3.32 $$\begin{aligned} \left\| {\text {curl}}\,{\textbf{u}}_{0}\right\| _{{\textbf{H}} ^{1/2}\left( \varOmega \right) }+\left| k\right| \left\| {\textbf{u}}_{0}\right\| \le C\left| k\right| ^{3 } \sqrt{1+\ln \left| k\right| }\left\| {\tilde{{\textbf{g}}}} _{T}\right\| _{{\textbf{L}}^{2}( \varGamma ) }. \end{aligned}$$

Combining (3.32) and (3.15) yields

3.33 $$\begin{aligned} \Vert {\textbf{u}}_{0}\Vert _{{\text {imp}},k}&\le C\left| k\right| ^{ 3 } \sqrt{1+\ln \left| k\right| }\left\| {\tilde{{\textbf{g}}}} _{T}\right\| _{{\textbf{L}}^{2}( \varGamma ) }\nonumber \\&\overset{(\text {3.14})}{\le }C\left| k\right| ^{ 3 } \sqrt{1+\ln \left| k\right| }\left( \left\| {\textbf{g}} _{T}\right\| _{{\textbf{L}}^{2}( \varGamma ) }+|k|^{1/2} \Vert {\textbf{j}}\Vert _{{\textbf{L}}^{2}(\varOmega )}\right) . \end{aligned}$$

*Step 7*: Combining (3.9), (3.13), and (3.33), we have arrived at

$$\begin{aligned} \left\| {\mathcal {S}}_{\varOmega ,k}^{{\text {MW}}}({\textbf{j}} ,{\textbf{g}}_{T})\right\| _{{\text {imp}},k}&\le \Vert k^{-2}\nabla \psi \Vert _{{\text {imp}},k}+\Vert {\textbf{u}} _{{\textbf{j}}}\Vert _{{\text {imp}},k}+\Vert {\textbf{u}}_{0}\Vert _{{\text {imp}},k}\\&\le C\left| k\right| ^{ 3 } \sqrt{1+\ln \left| k\right| }\left( \left\| {\textbf{g}} _{T}\right\| _{{\textbf{L}}^{2}( \varGamma ) }+|k|^{1/2} \Vert {\textbf{j}}\Vert _{{\textbf{L}}^{2}(\varOmega )}\right) , \end{aligned}$$

which is the claimed estimate. $\square$

#### Lemma 3.9

([35, Prop. 4.14]) Let $\varOmega \subset {\mathbb {R}}^{3}$ be a bounded Lipschitz domain. Then there is $C>0$ such that for every $w\in H^{1/2}(\varOmega )$ and every $t\in \left( 0,1\right]$ there exists some $w_{t}\in H^{1}(\varOmega )$ such that

3.34 $$\begin{aligned} \left\| w-w_{t}\right\| +t\left\| w_{t}\right\| _{H^{1}(\varOmega )}&\le Ct^{1/2}\left\| w\right\| _{H^{1/2}\left( \varOmega \right) }, \end{aligned}$$

3.35 $$\begin{aligned} \Vert w_{t}\Vert _{B_{2,1}^{1/2}(\varOmega )}&\le C\sqrt{1+\left| \ln t\right| }\left\| w\right\| _{H^{1/2}(\varOmega )}. \end{aligned}$$

## Maxwell’s Equations with the “Good” Sign

### Norms

We consider Maxwell’s equations with the “good” sign and first describe the spaces for the given data. Since the sesquilinear form $A_{k}\left( \cdot ,\cdot \right)$ is considered in the space ${\textbf{X}}_{{\text {imp}}}$, the natural space for the right-hand side is its dual ${\textbf{X}}_{{\text {imp}}}^{\prime }$. In our setting, the right-hand side is given in $\varOmega$ via the volume data ${\textbf{j}}$ and on $\varGamma$ via the boundary data ${\textbf{g}}_{T}$. In order to view ${\textbf{j}}$ and ${\textbf{g}}_T$ as elements of ${\textbf{X}}_{{\text {imp}}}^\prime$, we introduce the spaces ${\textbf{X}}_{{\text {imp}}}^{\prime }( \varOmega )$ and ${\textbf{X}}_{{\text {imp}}}^{\prime }( \varGamma )$. By using the usual notation $V^{\prime }$ for the dual space of a normed vector space *V* we define

4.1 $$\begin{aligned} {\textbf{X}}_{{\text {imp}},0}&:=\left\{ {\textbf{w}}\in {\textbf{X}} _{{\text {imp}}}:{\text {curl}}\,{\textbf{w}}=0\right\} ={\{\nabla \varphi \,|\,\varphi \in H_{{\text {imp}}}^{1}(\varOmega )\}},\nonumber \\ {\textbf{X}}_{{\text {imp}}}^{\prime }(\varOmega )&:=\left( {\textbf{H}} ^{1}(\varOmega )\right) ^{\prime }\cap {\textbf{X}}_{{\text {imp}},0}^{\prime }, \end{aligned}$$

4.2 $$\begin{aligned} {\textbf{H}}_{T}^{-1}(\mathop {{\text {div}}}\nolimits _{\varGamma },\varGamma )&:=\left\{ {\textbf{w}}\in {\textbf{H}}_{T}^{-1}(\varGamma )\mid \mathop {{\text {div}}}\nolimits _{\varGamma }\,{\textbf{w}}\in {\textbf{H}}_{T}^{-1}(\varGamma )\right\} ,\nonumber \\ {\textbf{X}}_{{\text {imp}}}^{\prime }(\varGamma )&:={\textbf{H}}_{T} ^{-1/2}(\varGamma )\cap {\textbf{H}}_{T}^{-1}\left( \mathop {{\text {div}}}\nolimits _{\varGamma },\varGamma \right) . \end{aligned}$$

and equip the spaces ${\textbf{X}}_{{\text {imp}}}^{\prime }(\varOmega )$ and ${\textbf{X}}_{{\text {imp}}}^{\prime }(\varGamma )$ with the norms (cf. also Lemma 4.1 below)

4.3 $$\begin{aligned} \Vert {\textbf{f}}\Vert _{{\textbf{X}}_{{\text {imp}}}^{\prime }(\varOmega ),k}&:=\sup _{{{\textbf{v}}}\in {{\textbf{X}}}_{{\text {imp}}}\setminus \{0\}} \frac{|({{\textbf{f}}},{{\textbf{v}}})|}{\Vert {{\textbf{v}}}\Vert _{{\text {imp}},k}}, \end{aligned}$$

4.4 $$\begin{aligned} \Vert {\textbf{g}}_{T}\Vert _{{\textbf{X}}_{{\text {imp}}}^{\prime }(\varGamma ),k}&:=\sup _{{{\textbf{v}}}\in {{\textbf{X}}}_{{{\text{ imp }}}\setminus \{0\} }}\frac{|({{\textbf{g}}}_{T},{{\textbf{v}}}_{T})_{{{\textbf{L}}}^{2}(\varGamma )}|}{\Vert {{\textbf{v}}}\Vert _{{\text {imp}},k}}. \end{aligned}$$

We also introduce for ${\textbf{g}}_{T}\in {\textbf{H}}_{T}^{-1/2}\left( \mathop {{\text {div}}}\nolimits _{\varGamma },\varGamma \right)$ (cf. (2.14))

4.5 $$\begin{aligned} \Vert {\textbf{g}}_{T}\Vert _{{\textbf{H}}^{-1/2}({\text {div}}_{\varGamma },\varGamma ),k}:=|k|\Vert {\text {div}}_{\varGamma }\,{\textbf{g}}_{T}\Vert _{H^{-1/2}(\varGamma )}+\left| k\right| ^{2}\Vert {\textbf{g}}_{T} \Vert _{{\textbf{X}}_{{\text {imp}}}^{\prime }\left( \varGamma \right) ,k}. \end{aligned}$$

An equivalent norm that is more naturally associated with the intersection spaces ${\textbf{X}}_{{\text {imp}}}^{\prime }(\varOmega )$ and ${\textbf{X}} _{{\text {imp}}}^{\prime }(\varGamma )$ is given in the following lemma.

#### Lemma 4.1

The spaces ${\textbf{X}}_{{\text {imp}} }^{\prime }(\varOmega )$ and ${\textbf{X}}_{{\text {imp}}}^{\prime }(\varGamma )$ can be viewed in a canonical way as subspaces of ${\textbf{X}}_{{\text {imp}} }^{\prime }$, and there holds the norm equivalences

4.6 $$\begin{aligned} \left\| {\textbf{f}}\right\| _{{\textbf{X}}_{{\text {imp}}}^{\prime }\left( \varOmega \right) ,k}&\sim \!\!\sup _{\varphi \in H_{{\text {imp}} }^{1}\left( \varOmega \right) :\nabla \varphi \ne {\textbf{0}}}\frac{|({\textbf{f}} ,\nabla \varphi )|}{\Vert \nabla \varphi \Vert _{{\text {imp}},k}} +\sup _{{\textbf{z}}\in {\textbf{H}}^{1}\left( \varOmega \right) \backslash \left\{ {\textbf{0}}\right\} }\frac{|({\textbf{f}},{\textbf{z}})|}{ \left| k\right| \left\| {\textbf{z}}\right\| _{{\textbf{H}} ^{1}(\varOmega ),k}} , \end{aligned}$$

4.7 $$\begin{aligned} \left\| {\textbf{g}}_{T}\right\| _{{\textbf{X}}_{{\text {imp}} }^{\prime }\left( \varGamma \right) ,k}&\sim \sup _{\varphi \in H_{{\text {imp}}}^{1}\left( \varOmega \right) :\nabla \varphi \ne {\textbf{0}}}\frac{|({\textbf{g}}_{T},\nabla _{\varGamma }\varphi )_{{\textbf{L}} ^{2}(\varGamma )}|}{\Vert \nabla \varphi \Vert _{{\text {imp}},k}}+\sup _{{\textbf{z}}\in {\textbf{H}}^{1}\left( \varOmega \right) \backslash \left\{ {\textbf{0}}\right\} }\frac{|({\textbf{g}}_{T},{\textbf{z}}_{T})_{{\textbf{L}} ^{2}(\varGamma )}|}{ \left| k\right| \left\| {\textbf{z}}\right\| _{{\textbf{H}} ^{1}(\varOmega ),k}} \end{aligned}$$

with constants implied in $\sim$ that are independent of $\left| k\right| \ge k_{0}$.

#### Proof

*Proof of* (4.6): Since $\nabla \varphi \in {\textbf{X}}_{{\text {imp}}}$ for $\varphi \in H_{{\text {imp}}} ^{1}(\varOmega )$ and ${{\textbf{H}}}^{1}(\varOmega )\subset {{\textbf{X}}} _{{\text {imp}}}$, the right-hand side of (4.6) is easily bounded by the left-hand side. For the reverse estimate, we decompose any element ${\textbf{v}}\in {\textbf{X}}_{{\text {imp}}}$ with the aid of Lemma 2.7 as ${\textbf{v}}=\nabla \varphi +{\textbf{z}}$ with $\Vert \nabla \varphi \Vert _{{\textbf{L}}^{2}(\varOmega )}+\Vert {\textbf{z}}\Vert _{{\textbf{L}}^{2}(\varOmega )}\le C\Vert {\textbf{v}} \Vert _{{\textbf{L}}^{2}(\varOmega )}$ and $\Vert {\textbf{z}}\Vert _{{\textbf{H}} ^{1}(\varOmega )}\le C\Vert {\textbf{v}}\Vert _{{\textbf{H}}({\text {curl}},\varOmega )}$. Hence,

4.8 $$\begin{aligned} \Vert \nabla \varphi \Vert _{{\text {imp}},k}+|k|\Vert {\textbf{z}} \Vert _{{\textbf{H}}^{1}(\varOmega ),k}&\le C\left( |k|^{1/2}(\Vert {\textbf{v}} _{T}\Vert _{{\textbf{L}}^{2}(\varGamma )}+\Vert {\textbf{z}}_{T}\Vert _{{\textbf{L}} ^{2}(\varGamma )})+\Vert {\textbf{v}}\Vert _{{\text {imp}},k}\right) \nonumber \\&\le C\Vert {\textbf{v}}\Vert _{{\text {imp}},k}, \end{aligned}$$

where, in the last step we used the multiplicative trace estimate $\Vert {\textbf{z}}_{T}\Vert _{{\textbf{L}}^{2}(\varGamma )}^{2}\le C\Vert {\textbf{z}}\Vert _{{\textbf{L}}^{2}(\varOmega )}\Vert {\textbf{z}}\Vert _{{\textbf{H}} ^{1}(\varOmega )}$. This implies that the left-hand side of (4.6) can be bounded by the right-hand side.

*Proof of* (4.7): The proof is analogous to that of (4.6). $\square$

Note that ${\textbf{L}}^{2}(\varOmega )\subset {\textbf{X}}_{{\text {imp}} }^{\prime }(\varOmega )$ and ${{\textbf{L}}}_{T}^{2}(\varGamma )\subset {\textbf{X}} _{{\text {imp}}}^{\prime }(\varGamma )$ with continuous embeddings as can be seen from the following reasoning. For $m\in {\mathbb {N}}_{0}$ and ${\textbf{f}} \in {\textbf{L}}^{2}(\varOmega )$ or ${\textbf{f}}\in {\textbf{H}}^{m}(\varOmega )$ or ${\textbf{f}}\in {\textbf{H}}^{m}({\text {div}},\varOmega )$ and for ${\textbf{g}}_{T}\in {\textbf{L}}_{T}^{2}(\varGamma )$ or ${\textbf{g}}_{T}\in {\textbf{H}}_{T}^{m+1/2}(\varGamma )$ or ${\textbf{g}}_{T}\in {\textbf{H}}_{T} ^{m+1/2}(\mathop {{\text {div}}}\nolimits _{\varGamma },\varGamma )$, we have by direct estimations

4.9 $$\begin{aligned} \Vert {\textbf{f}}\Vert _{{\textbf{X}}_{{\text {imp}}}^{\prime }( \varOmega ) ,k}&\le C|k|^{-1}\Vert {\textbf{f}}\Vert _{{\textbf{L}} ^{2}(\varOmega )}\!\le \! C|k|^{-1}\Vert {\textbf{f}}\Vert _{{\textbf{H}}^{m}(\varOmega ),k}\!\le \! C|k|^{-2}\Vert {\textbf{f}}\Vert _{{\textbf{H}}^{m}({\text {div}},\varOmega ),k},\!\! \end{aligned}$$

4.10 $$\begin{aligned} \Vert {\textbf{g}}_{T}\Vert _{{\textbf{X}}_{{\text {imp}}}^{\prime }( \varGamma ) ,k}&\le C|k|^{-1/2}\Vert {\textbf{g}}_{T}\Vert _{{\textbf{L}} ^{2}(\varGamma )}\le C|k|^{-1}\Vert {\textbf{g}}_{T}\Vert _{{\textbf{H}}^{m+1/2} (\varGamma ),k} \nonumber \\&\le C\left| k\right| ^{-2}\Vert {\textbf{g}}_{T} \Vert _{{\textbf{H}}^{m+1/2}({\text {div}}_{\varGamma },\varGamma ),k} , \end{aligned}$$

4.11 $$\begin{aligned} \Vert {\textbf{g}}_{T}\Vert _{{\textbf{H}}^{-1/2}({\text {div}}_{\varGamma } ,\varGamma ),k}&\le C|k|\Vert {\textbf{g}}_{T}\Vert _{{\textbf{H}}^{1/2}(\varGamma ),k}. \end{aligned}$$

We also have the following result for $\Vert {\textbf{g}}_{T}\Vert _{{\textbf{H}} ^{-1/2}({\text {div}}_{\varGamma },\varGamma ),k}$:

#### Lemma 4.2

There is $C>0$ depending only on $\varOmega$ such that

$$\begin{aligned} \Vert {\textbf{g}}_{T}\Vert _{{\textbf{H}}^{-1/2}({\text {div}}_{\varGamma },\varGamma ),k}\le C\Vert {\text {div}}_{\varGamma }\,{\textbf{g}}_{T}\Vert _{H^{-1/2}(\varGamma ),k}+|k|\Vert {\textbf{g}}_{T}\Vert _{ {\textbf{H}}^{-1/2}(\varGamma ),k}. \end{aligned}$$

#### Proof

We use the minimum norm lifting ${\mathcal {E}}_{\varOmega }^{\varDelta }$ from (6.13) with the property that $\Vert \nabla \varphi \Vert _{{\textbf{L}}^{2}(\varOmega )}\ge \Vert \nabla {\mathcal {E}}_{\varOmega }^{\varDelta } (\varphi |_{\varGamma })\Vert _{{\textbf{L}}^{2}(\varOmega )}$ for arbitrary $\varphi \in H_{{\text {imp}}}^{1}(\varOmega )$. By continuity of the trace mapping, we get $\inf _{c\in {\mathbb {R}}}\Vert \varphi -c\Vert _{H^{1/2}(\varGamma )}\le C\Vert \nabla {\mathcal {E}}_{\varOmega }^{\varDelta }(\varphi |_{\varGamma })\Vert _{{\textbf{L}}^{2}(\varOmega )}\le C\Vert \nabla \varphi \Vert _{{\textbf{L}}^{2} (\varOmega )}$. An integration by parts shows for arbitrary $\varphi \in H_{{\text {imp}}}^{1}(\varOmega )$ and arbitrary $c\in {\mathbb {R}}$

$$\begin{aligned} |({\textbf{g}}_{T},\nabla _{\varGamma }\varphi )_{{\textbf{L}}^{2}(\varGamma )} |=|({\text {div}}_{\varGamma }\,{\textbf{g}}_{T},\varphi -c)_{{\textbf{L}} ^{2}(\varGamma )}|\le \Vert {\text {div}}_{\varGamma }\,{\textbf{g}}_{T} \Vert _{H^{-1/2}(\varGamma )}\Vert \varphi -c\Vert _{H^{1/2}(\varGamma )}. \end{aligned}$$

Taking the infimum over all $c\in {\mathbb {R}}$ yields, for arbitrary $\varphi \in H_{{\text {imp}}}^{1}(\varOmega )$,

$$\begin{aligned} |({\textbf{g}}_{T},\nabla _{\varGamma }\varphi )_{{\textbf{L}}^{2}(\varGamma )}|\le C\Vert {\text {div}}_{\varGamma }\,{\textbf{g}}_{T}\Vert _{H^{-1/2}(\varGamma )} \Vert \nabla \varphi \Vert _{{\textbf{L}}^{2}(\varOmega )}, \end{aligned}$$

and we conclude

$$\begin{aligned} \sup _{\varphi \in H_{{\text {imp}}}^{1}(\varOmega ):\nabla _{\varGamma } \varphi \ne {\textbf{0}}}\frac{|({\textbf{g}}_{T},\nabla _{\varGamma }\varphi )_{{\textbf{L}}^{2}(\varGamma )}|}{|k|^{1/2}\Vert \nabla _{\varGamma }\varphi \Vert _{{\textbf{L}}^{2}(\varGamma )}+|k|\Vert \nabla \varphi \Vert _{{\textbf{L}} ^{2}(\varOmega )}}\le C|k|^{-1}\Vert {\text {div}}_{\varGamma }\,{\textbf{g}} _{T}\Vert _{H^{-1/2}(\varGamma )}. \end{aligned}$$

Similarly, for ${\textbf{z}}\in {\textbf{H}}^{1}(\varOmega )$ we estimate $|({\textbf{g}}_{T},{\textbf{z}}_{T})_{{\textbf{L}}^{2}(\varGamma )}|\le C\Vert {\textbf{g}}_{T}\Vert _{{\textbf{H}}^{-1/2}(\varGamma )}\Vert {\textbf{z}}\Vert _{{\textbf{H}}^{1}(\varOmega )}$. Hence,

4.12 $$\begin{aligned} \Vert {\textbf{g}}_{T}\Vert _{{\textbf{X}}_{{\text {imp}}}^{\prime }\left( \varGamma \right) ,k}\le C\left( \left| k\right| ^{-1}\Vert {\text {div}}_{\varGamma }\,{\textbf{g}}_{T}\Vert _{H^{-1/2}(\varGamma )} +\Vert {\textbf{g}}_{T}\Vert _{{\textbf{H}}^{-1/2}(\varGamma )}\right) . \end{aligned}$$

The result follows. $\square$

### The Maxwell Problem with the Good Sign

The Maxwell problem with the good sign reads: Given $\displaystyle {\textbf{f}} \in {\textbf{X}}_{{\text {imp}}}^{\prime }(\varOmega )$ and ${\textbf{g}}_{T} \in {\textbf{X}}_{{\text {imp}}}^{\prime }(\varGamma ),$ find ${\textbf{v}} \in {\textbf{X}}_{{\text {imp}}}$ such that

4.13 $$\begin{aligned} {\mathcal {L}}_{\varOmega ,{\text {i}}\,k}{\textbf{v}}={\textbf{f}}\text { in } \varOmega \quad \text {and}\quad {\mathcal {B}}_{ \varGamma ,k}{\textbf{v}}={\textbf{g}}_{T}\text { on }\varGamma . \end{aligned}$$

The weak formulation is:

4.14 $$\begin{aligned} \text {find }{\textbf{z}}\in {\textbf{X}}_{{\text {imp}}}\quad \text {s.t.}\quad A_{k}^{+}({\textbf{z}}, {\textbf{v}} )=\left( {\textbf{f}},{\textbf{v}}\right) +\left( {\textbf{g}}_{T},{\textbf{v}} _{T}\right) _{{\textbf{L}}^{2}\left( \varGamma \right) }\quad \forall {\textbf{v}} \in {\textbf{X}}_{{\text {imp}}}, \end{aligned}$$

where the sesquilinear form $A_{k}^{+}$ is given by

4.15 $$\begin{aligned} A_{k}^{+}({\textbf{u}},{\textbf{v}}):=\left( {\text {curl}}\,\textbf{u,}\, {\text {curl}}\,{\textbf{v}}\right) +k^{2}\left( \textbf{u,v}\right) -{\text {i}}\,k\left( {\textbf{u}}_{T},{\textbf{v}}_{T}\right) _{{\textbf{L}}^{2}(\varGamma )}. \end{aligned}$$

The solution operator is denoted $({\textbf{f}},{\textbf{g}}_{T})\mapsto {\mathcal {S}}_{\varOmega ,k}^{+}({\textbf{f}},{\textbf{g}}_{T}$). In this section, we develop the regularity theory for problem (4.13). Indeed, as the following Theorem 4.3 shows, (4.14) is uniquely solvable.

#### Theorem 4.3

Let $\varOmega$ be a bounded Lipschitz domain with simply connected boundary. Then there is $C>0$ independent of *k* such that the following holds:

(i) The sesquilinear form $A_{k}^{+}$ satisfies ${\text {Re}} A_{k}^{+}({\textbf{v}},\sigma {\textbf{v}}) = 2^{-1/2} \Vert {\textbf{v}}\Vert ^{2}_{{\text {imp}},k}$ for all ${\textbf{v}} \in {\textbf{X}}_{{\text {imp}}}$, where $\sigma =\exp \left( \frac{\pi {\text {i}}\,}{4}{\text {sign}}k\right)$.
(ii) The sesquilinear form is continuous: $|A_{k}^{+}({\textbf{u}},{\textbf{v}})| \le \Vert {\textbf{u}} \Vert _{{\text {imp}},k} \Vert {\textbf{v}}\Vert _{{\text {imp}},k}$ for all ${\textbf{u}}$, ${\textbf{v}} \in {\textbf{X}}_{{\text {imp}}}$.
(iii) The solution ${\textbf{u}} \in {\textbf{X}}_{{\text {imp}}}$ of (4.13) satisfies
  4.16 $$\begin{aligned} \left\| {\textbf{u}}\right\| _{{\text {imp}},k}&\le C\left( |k|^{-1}\left\| {\textbf{f}}\right\| _{{\textbf{L}}^{2}(\varOmega )}+\left| k\right| ^{-1/2}\left\| {\textbf{g}}_{T}\right\| _{{\textbf{L}} ^{2}(\varGamma )}\right) , \end{aligned}$$
  4.17 $$\begin{aligned} \left\| {\textbf{u}}\right\| _{{\text {imp}},k}&\le C\left( \left\| {\textbf{f}}\right\| _{{\textbf{X}}_{{\text {imp}}}^{\prime }( \varOmega ) ,k}+\left\| {\textbf{g}}_{T}\right\| _{{\textbf{X}}_{{\text {imp}}}^{\prime }( \varGamma ) ,k}\right) , \end{aligned}$$
  provided $\left( {\textbf{f}},{\textbf{g}}_{T}\right) \in {\textbf{L}}^{2}( \varOmega ) \times {\textbf{L}}_{T}^{2}( \varGamma )$ for (4.16) and $\left( {\textbf{f}},{\textbf{g}}_{T}\right) \in {\textbf{X}}_{{\text {imp}}}^{\prime }( \varOmega ) \times {\textbf{X}}_{{\text {imp}}}^{\prime }( \varGamma )$ for (4.17).
(iv) Let $m\in {\mathbb {N}} _{0}$. If $\varGamma$ is sufficiently smooth and ${\textbf{f}}\in {\textbf{H}} ^{m}({\text {div}},\varOmega )$, ${\textbf{g}}_{T}\in {\textbf{H}}_{T} ^{m+1/2}( \varGamma )$, then
  4.18a $$\begin{aligned} \left\| {\textbf{u}}\right\| _{{\textbf{H}}^{m+1}\left( \varOmega \right) ,k}&\le C\left| k\right| ^{-3}\left( \left\| {\textbf{f}}\right\| _{{\textbf{H}}^{m}({\text {div}},\varOmega ),k}+\left\| {\textbf{g}} _{T}\right\| _{{\textbf{H}}^{m-1/2}({\text {div}}_{\varGamma },\varGamma ),k}\right) , \end{aligned}$$
  4.18b $$\begin{aligned} \left\| {\textbf{u}}\right\| _{{\textbf{H}}^{m+1}\left( {\text {curl}},\varOmega \right) ,k}&\le C\left| k\right| ^{-2}\left( \left\| {\textbf{f}}\right\| _{{\textbf{H}}^{m} ({\text {div}},\varOmega ),k}+|k|\left\| {\textbf{g}}_{T}\right\| _{{\textbf{H}}^{m+1/2}\left( \varGamma \right) ,k}\right) . \end{aligned}$$

#### Proof

*Proof of* (i), (ii): For (i) we compute

$$\begin{aligned} {\text {Re}}\left( A_{k}^{+}({\textbf{v}},\sigma {\textbf{v}})\right) ={\text {Re}}\left( {\bar{\sigma }}\left\| {\textbf{v}}\right\| _{{\textbf{H}}\left( {\text {curl}},\varOmega \right) ,k}^{2} +{\text {i}}\,{\bar{\sigma }}k\left\| {\textbf{v}}_{T}\right\| _{{\textbf{L}}^{2}\left( \varGamma \right) }^{2}\right) =\frac{\sqrt{2}}{2}\left\| {\textbf{v}}\right\| _{{\text {imp}},k}^{2}. \end{aligned}$$

The continuity assertion (ii) follows by the Cauchy-Schwarz inequality.

*Proof of* (iii): The estimate (iii) follows directly from a variant of the Lax-Milgram lemma: We choose ${\textbf{v}}=\sigma {{\textbf {u}}}$ in the weak form (4.14) and estimate

4.19 $$\begin{aligned} \frac{\sqrt{2}}{2}\left\| {\textbf{u}}\right\| _{{\text {imp}} ,k}^{2}&={\text {Re}}\,A^{+}({\textbf{u}},\sigma {\textbf{u}} )={\text {Re}}\,\left( \left( {\textbf{f}},\sigma {\textbf{u}}\right) +\left( {\textbf{g}}_{T},\sigma {\textbf{u}}_{T}\right) _{{\textbf{L}}^{2}(\varGamma )}\right) \nonumber \\&\le \left( \Vert {\textbf{f}}\Vert _{{{\textbf{X}}}_{{\text {imp}}} ^{\prime }(\varOmega ),k}+\Vert {\textbf{g}}_{T}\Vert _{{{\textbf{X}}} _{{\text {imp}}}^{\prime }(\varGamma ),k}\right) \Vert {{\textbf{u}}} \Vert _{{\text {imp}},k} \end{aligned}$$

from which (4.17) follows. Estimate (4.16) is then obtained from (4.17) and (4.9), (4.10).

*Proof of* (iv): From now on, we assume that $\varGamma$ is sufficiently smooth. We proceed by induction on $m\in {\mathbb {N}}_{0}$ and show that if the solution ${\textbf{u}}\in {\textbf{H}}^{m}({\text {curl}},\varOmega )$, then ${\textbf{u}}\in {\textbf{H}}^{m+1}({\text {curl}},\varOmega )$. Specifically, after the preparatory Step 1, we will show ${\textbf{u}}\in {\textbf{H}}^{m+1}(\varOmega )$ in Step 2 and ${\text {curl}}\,{\textbf{u}}\in {\textbf{H}}^{m+1}(\varOmega )$ in Step 3. Step 4 shows the induction hypothesis for $m=0$ including the norm bounds. Step 5 completes the induction argument for the norm bounds.

*Step 1*: Taking the surface divergence of the boundary conditions we get by using the differential equation

4.20 $$\begin{aligned} -{\text {i}}\,k{\text {div}}_{\varGamma }\,{\textbf{u}}_{T}&={\text {div}}_{\varGamma }\,{\textbf{g}}_{T}-\,{\text {div}}_{\varGamma }\,\left( \gamma _{T}{\text {curl}}\,{\textbf{u}}\right) \overset{\text {[43, (2.5.197)]}}{=}{\text {div}}_{\varGamma }\, {\textbf{g}}_{T}+{\text {curl}}_{\varGamma }\,{\text {curl}}\,{\textbf{u}} \nonumber \\&={\text {div}}_{\varGamma }\,{\textbf{g}}_{T}+\langle {\text {curl}}\,{\text {curl}}\,{\textbf{u}},{\textbf{n}}\rangle ={\text {div}}_{\varGamma }\,{\textbf{g}}_{T}+\langle {\textbf{f}} -k^{2}{\textbf{u}},{\textbf{n}}\rangle . \end{aligned}$$

We note that ${\text {div}}({\textbf{f}}-k^{2}{\textbf{u}})=0$ so that

4.21 $$\begin{aligned} \Vert \langle {\textbf{f}}-k^{2}{\textbf{u}},{\textbf{n}}\rangle \Vert _{{\textbf{H}} ^{m-1/2}(\varGamma )}\le C\Vert {\textbf{f}}-k^{2}{\textbf{u}}\Vert _{{\textbf{H}} ^{m}(\varOmega )}. \end{aligned}$$

Inserting this in (4.20) yields

4.22 $$\begin{aligned} \left\| {\text {div}}_{\varGamma }\,{\textbf{u}}_{T}\right\| _{{\textbf{H}} ^{m-1/2}( \varGamma ) }\le & {} C|k|^{-1}\left[ \left\| {\text {div}}_{\varGamma }\,{\textbf{g}}_{T}\right\| _{{\textbf{H}} ^{m-1/2}( \varGamma ) }\right. \nonumber \\{} & {} \quad \left. +\left\| {\textbf{f}}\right\| _{{\textbf{H}}^{m}(\varOmega )}+\left| k\right| ^{2}\left\| {\textbf{u}}\right\| _{{\textbf{H}}^{m}\left( \varOmega \right) }\right] . \end{aligned}$$

It will be convenient to abbreviate

4.23 $$\begin{aligned} \begin{aligned} R_{m}&:=|k|^{-1}\Bigl [ \left\| {\text {div}}_{\varGamma }\,{\textbf{g}} _{T}\right\| _{{\textbf{H}}^{m-1/2}\left( \varGamma \right) }+\left\| {\textbf{f}}\right\| _{{\textbf{H}}^{m}(\varOmega )}+|k|^{-1}\left\| {\textbf{f}}\right\| _{{\textbf{H}}^{m}({\text {div}},\varOmega )} \\&\quad + \left| k\right| ^{2}\left\| {\textbf{u}}\right\| _{{\textbf{H}}^{m}\left( \varOmega \right) }+|k|\left\| {\textbf{u}}\right\| _{{\textbf{H}}^{m}\left( {\text {curl}},\varOmega \right) }\Bigr ]. \end{aligned} \end{aligned}$$

*Step 2* (${\textbf{H}}^{m+1}(\varOmega )$-estimate): With the aid of Lemma 2.7 (ii), we write ${\textbf{u}} =\nabla \varphi +{\textbf{z}}$ with $\varphi \in H^{m+1}(\varOmega )$ and ${\textbf{z}}\in {\textbf{H}}^{m+1}(\varOmega )$ and

4.24 $$\begin{aligned} \Vert \varphi \Vert _{{\textbf{H}}^{m+1}(\varOmega )}+\Vert {\textbf{z}}\Vert _{{\textbf{H}}^{m}(\varOmega )}&\le C\Vert {\textbf{u}}\Vert _{{\textbf{H}} ^{m}(\varOmega )}\overset{(\text {4.23}) }{\le }C|k|^{-1}R_{m}, \end{aligned}$$

4.25 $$\begin{aligned} \Vert {\textbf{z}}\Vert _{{\textbf{H}}^{m+1}(\varOmega )}&\le C\Vert {\textbf{u}} \Vert _{{\textbf{H}}^{m}({\text {curl}},\varOmega )}\overset{ (\text {4.23})}{\le }CR_{m}. \end{aligned}$$

*Step 2a*: We bound

4.26 $$\begin{aligned} \left\| \mathop {{\text {div}}}\nolimits _{\varGamma }\,{\textbf{z}}_{T}\right\| _{{\textbf{H}}^{m-1/2}\left( \varGamma \right) }\le C\Vert {\textbf{z}}_{T} \Vert _{{\textbf{H}}^{m+1/2}(\varGamma )}\le C\Vert {\textbf{z}}\Vert _{{\textbf{H}} ^{m+1}(\varOmega )}\overset{(\text {4.25})}{\le }CR_{m}. \end{aligned}$$

*Step 2b*: Applying ${\text {div}}_{\varGamma }\varPi _{T}$ to the decomposition of ${\textbf{u}}$ leads to

4.27 $$\begin{aligned} \varDelta _{\varGamma }\varphi |_{\varGamma }={\text {div}}_{\varGamma }\,\nabla _{\varGamma }\varphi ={\text {div}}_{\varGamma }\,{\textbf{u}}_{T}-{\text {div}}_{\varGamma }\,{\textbf{z}}_{T} \end{aligned}$$

with

4.28 $$\begin{aligned} \left\| {\text {div}}_{\varGamma }\,{\textbf{u}}_{T}-{\text {div}} _{\varGamma }\,{\textbf{z}}_{T}\right\| _{{\textbf{H}}^{m-1/2}\left( \varGamma \right) }\overset{(\text {4.22}),(\text {4.26}),(\text {4.23})}{\le }CR_{m}. \end{aligned}$$

Together with (4.27), we infer $\varphi |_{\varGamma }\in H^{m+3/2}(\varGamma )$. Since $\varGamma$ is connected, $\varphi |_{\varGamma }$ is unique up to a constant. We select this constant such that $\varphi |_{\varGamma }$ has zero mean. Elliptic regularity implies

$$\begin{aligned} \left\| \varphi \right\| _{H^{3/2+m}\left( \varGamma \right) }\le C\left\| {\text {div}}_{\varGamma }\,{\textbf{u}}_{T}-{\text {div}} _{\varGamma }\,{\textbf{z}}_{T}\right\| _{H^{-1/2+m}\left( \varGamma \right) }\overset{(\text {4.28})}{\le }CR_{m}. \end{aligned}$$

The function $\varphi$ satisfies the following Dirichlet problem:

$$\begin{aligned} \varDelta \varphi ={\text {div}}\,{\textbf{u}}-{\text {div}}\,{\textbf{z}} =k^{-2}{\text {div}}\,{\textbf{f}}-{\text {div}}\,{\textbf{z}}\in H^{m}(\varOmega ),\qquad \varphi |_{\varGamma }\in H^{3/2+m}( \varGamma ), \end{aligned}$$

from which we get by elliptic regularity

$$\begin{aligned} \left\| \varphi \right\| _{H^{2+m}( \varOmega ) }\le C\left( \Vert \varphi \Vert _{H^{3/2+m}( \varGamma ) }+\left| k\right| ^{-2}\Vert {\text {div}}\,{\textbf{f}}\Vert _{{\textbf{H}}^{m}(\varOmega )}+\left\| {\text {div}}\,{\textbf{z}}\right\| _{H^{m}(\varOmega )}\right) \le CR_{m}. \end{aligned}$$

We conclude

4.29 $$\begin{aligned} \Vert {\textbf{u}}\Vert _{{\textbf{H}}^{m+1}(\varOmega )}\le CR_{m}. \end{aligned}$$

*Step 3* (${\textbf{H}}^{m+1}( {\text {curl}},\varOmega )$-estimate): We set ${\textbf{w}}:={\text {curl}}\,{\textbf{u}}$. Since ${\textbf{u}}\in {\textbf{H}}^{m+1}( \varOmega )$ (cf. (4.29)) we know that ${\textbf{w}}\in {\textbf{H}}^{m}( \varOmega )$. As in Step 2 we write ${\textbf{w}}=\nabla {\tilde{\varphi }}+{\tilde{{\textbf{z}}}}$ and obtain

$$\begin{aligned} \Vert {\tilde{\varphi }}\Vert _{H^{m+1}(\varOmega )}+\Vert {\tilde{{\textbf{z}}}} \Vert _{{\textbf{H}}^{m}(\varOmega )}&\le C\Vert {\textbf{w}}\Vert _{{\textbf{H}} ^{m}(\varOmega )}\le C\Vert {\textbf{u}}\Vert _{{\textbf{H}}^{m+1}(\varOmega )} \overset{(\text {4.29})}{\le }CR_{m},\\ \Vert {\tilde{{\textbf{z}}}}\Vert _{{\textbf{H}}^{m+1}(\varOmega )}&\le C\Vert {\textbf{w}}\Vert _{{\textbf{H}}^{m}({\text {curl}},\varOmega )}\le C\left( \Vert {\text {curl}}\,{\textbf{w}}\Vert _{{\textbf{H}}^{m}(\varOmega )} +\Vert {\textbf{w}}\Vert _{{\textbf{H}}^{m}(\varOmega )}\right) \\&\le C\left( \Vert {\text {curl}}\,{\text {curl}}\,{\textbf{u}} \Vert _{{\textbf{H}}^{m}(\varOmega )}+\Vert {\textbf{u}}\Vert _{{\textbf{H}}^{m+1}(\varOmega )}\right) \\&\le C\left( \Vert {\textbf{f}}-k^{2}{\textbf{u}}\Vert _{{\textbf{H}} ^{m}(\varOmega )}+R_{m}\right) . \end{aligned}$$

To estimate the norm of ${\tilde{\varphi }}$, we employ the boundary condition satisfied by ${\textbf{u}}$, i.e.,

$$\begin{aligned} \nabla _{\varGamma }{\tilde{\varphi }}={\textbf{n}}\times \gamma _{T}\nabla \tilde{\varphi }={\textbf{n}}\times \left( \gamma _{T}{\textbf{w}}-\gamma _{T}{\tilde{{\textbf{z}}} }\right) ={\textbf{n}}\times \left( {\textbf{g}}_{T}+{\text {i}}\, k{\textbf{u}}_{T}-\gamma _{T}{\tilde{{\textbf{z}}}}\right) . \end{aligned}$$

In view of ${\textbf{g}}_{T}\in {\textbf{H}}_{T}^{m+1/2}\left( \varGamma \right)$, this implies ${\tilde{\varphi }}|_{\varGamma }\in H^{m+3/2}\left( \varGamma \right)$ with

$$\begin{aligned} \left\| \nabla _{\varGamma }{\tilde{\varphi }}\right\| _{{\textbf{H}} ^{m+1/2}( \varGamma ) }&\le C\left( \Vert {\textbf{g}}_{T} \Vert _{{\textbf{H}}^{m+1/2}( \varGamma ) }+\left| k\right| \left\| {\textbf{u}}_{T}\right\| _{{\textbf{H}}^{m+1/2}( \varGamma ) }+\left\| \gamma _{T}{\tilde{{\textbf{z}}}}\right\| _{{\textbf{H}}^{m+1/2}( \varGamma ) }\right) \\&\le C\left( \Vert {\textbf{g}}_{T}\Vert _{{\textbf{H}}^{m+1/2}(\varGamma )}+|k|\Vert {\textbf{u}}\Vert _{{\textbf{H}}^{m+1}(\varOmega )}+\Vert {\tilde{{\textbf{z}}} }\Vert _{{\textbf{H}}^{m+1}(\varOmega )}\right) \\&\le C\left( \Vert {\textbf{g}}_{T}\Vert _{{\textbf{H}}^{m+1/2}(\varGamma )}+|k|R_{m}\right) . \end{aligned}$$

The function ${\tilde{\varphi }}$ solves the Dirichlet problem

4.30 $$\begin{aligned} \varDelta {\tilde{\varphi }}={\text {div}}({\textbf{w}}-{\tilde{{\textbf{z}}} })=-{\text {div}}\,{\tilde{{\textbf{z}}}}\quad \text {in }\varOmega ,\qquad {\tilde{\varphi }}|_{\varGamma }\in H^{m+3/2}\left( \varGamma \right) . \end{aligned}$$

Since ${\tilde{\varphi }}|_{\varGamma }$ is determined up to a constant, we may assume that ${\tilde{\varphi }}|_{\varGamma }$ has vanishing mean. Elliptic regularity theory for (4.30) tells us that

$$\begin{aligned} \left\| \nabla {\tilde{\varphi }}\right\| _{{\textbf{H}}^{m+1}(\varOmega )}&\le C\left( \left\| \nabla _{\varGamma }{\tilde{\varphi }}\right\| _{{\textbf{H}} ^{m+1/2}(\varGamma )}^{2}+\left\| {\text {div}}\,{\tilde{{\textbf{z}}} }\right\| _{H^{m}(\varOmega )}^{2}\right) ^{1/2} \\&\le C\left( \Vert {\textbf{g}}_{T}\Vert _{{\textbf{H}}^{m+1/2}(\varGamma )}+|k|R_{m}\right) . \end{aligned}$$

We obtain ${\textbf{w}}\in {\textbf{H}}^{m+1}(\varOmega )$ with

4.31 $$\begin{aligned} \left| {\text {curl}}\,{\textbf{u}}\right| _{{\textbf{H}}^{m+1} (\varOmega )}&=\left| {\textbf{w}}\right| _{{\textbf{H}}^{m+1}(\varOmega )} \le C\left( \left| \nabla {\tilde{\varphi }}\right| _{{\textbf{H}} ^{m+1}(\varOmega )}+\left| \nabla {\tilde{{\textbf{z}}}}\right| _{{\textbf{H}}^{m}(\varOmega )}\right) \nonumber \\&\le C\left( \Vert {\textbf{g}}_{T} \Vert _{{\textbf{H}}^{m+1/2}(\varGamma )}+|k|R_{m}\right) . \end{aligned}$$

*Step 4*: We ascertain the bounds (4.18a), (4.18b) for $m=0$. We have

$$\begin{aligned} \Vert {\textbf{u}}\Vert _{{\text {imp}},k}&\overset{(\text {4.17}), (\text {4.9}), (\text {4.5})}{\le }C\left| k\right| ^{-2}\left( \Vert {\textbf{f}}\Vert _{{\textbf{H}}({\text {div}},\varOmega ),k}+\Vert {\textbf{g}}_{T}\Vert _{{\textbf{H}}^{-1/2}({\text {div}}_{\varGamma },\varGamma ),k}\right) ,\\ \Vert {\text {div}}_{\varGamma }\,{\textbf{g}}_{T}\Vert _{{\textbf{H}}^{-1/2} (\varGamma )}&\overset{(\text {4.5})}{\le }|k|^{-1}\Vert {\textbf{g}} _{T}\Vert _{{\textbf{H}}^{-1/2}\left( {\text {div}}_{\varGamma },\varGamma \right) ,k}. \end{aligned}$$

This implies for $R_{0}$ from (4.23)

4.32 $$\begin{aligned} R_{0}\le C\left| k\right| ^{-2}\left( \Vert {\textbf{f}}\Vert _{{\textbf{H}}({\text {div}},\varOmega ),k}+\Vert {\textbf{g}}_{T}\Vert _{{\textbf{H}}^{-1/2}({\text {div}}_{\varGamma },\varGamma ),k}\right) \end{aligned}$$

and in turn from (4.29)

$$\begin{aligned} \Vert {\textbf{u}}\Vert _{{\textbf{H}}^{1}(\varOmega ),k}&\le C\left( \Vert {\textbf{u}}\Vert _{{\textbf{L}}^{2}(\varOmega )}+|k|^{-1}\Vert {\textbf{u}} \Vert _{{\textbf{H}}^{1}(\varOmega )}\right) \overset{(\text {4.29}),(\text {4.23})}{\le }C|k|^{-1}R_{0}\\&\le C|k|^{-3}\left( \Vert {\textbf{f}}\Vert _{{\textbf{H}}({\text {div}} ,\varOmega ),k}+\Vert {\textbf{g}}_{T}\Vert _{{\textbf{H}}^{-1/2}({\text {div}} _{\varGamma },\varGamma ),k}\right) , \end{aligned}$$

which is formula (4.18a) for $m=0$. Next,

$$\begin{aligned} \Vert {\textbf{u}}\Vert _{{\textbf{H}}^{1}({\text {curl}},\varOmega ),k}&\le C\left( |k|^{-1}\Vert {\text {curl}}\,{\textbf{u}}\Vert _{{\textbf{H}} ^{1}(\varOmega )}+|k|\Vert {\textbf{u}}\Vert _{{\textbf{H}}^{1}(\varOmega ),k}\right) \\&\overset{(\text {4.31})}{\le }C\left( |k|^{-1}\Vert {\textbf{g}} _{T}\Vert _{{\textbf{H}}^{1/2}(\varGamma )}+R_{0}\right) \\&\overset{(\text {4.32}), (\text {4.11})}{\le }C\left( |k|^{-1}\Vert {\textbf{g}}_{T} \Vert _{{\textbf{H}}^{1/2}(\varGamma ),k}+\left| k\right| ^{-2} \Vert {\textbf{f}}\Vert _{{\textbf{H}}({\text {div}},\varOmega ),k}\right) , \end{aligned}$$

which is formula (4.18b) for $m=0$.

We now assume that the estimates (4.18a), (4.18b) holds up to *m* and show that they hold for $m+1$. Introduce the abbreviations

$$\begin{aligned} T_{1}(m)&:=\Vert {\textbf{f}}\Vert _{{\textbf{H}}^{m}({\text {div}} ,\varOmega ),k}+\Vert {\textbf{g}}_{T}\Vert _{{\textbf{H}}^{m-1/2}({\text {div}} _{\varGamma },\varGamma ),k},\\ T_{2}(m)&:=\Vert {\textbf{f}}\Vert _{{\textbf{H}}^{m}({\text {div}} ,\varOmega ),k}+\left| k\right| \Vert {\textbf{g}}_{T}\Vert _{{\textbf{H}} ^{m+1/2}(\varGamma ),k}. \end{aligned}$$

It is easy to verify that (using (4.11) for the case $m = 0$)

4.33 $$\begin{aligned} T_{1}\left( m\right) \le CT_{2}\left( m\right) \le CT_{1}\left( m+1\right) . \end{aligned}$$

By the induction hypothesis, we have

4.34 $$\begin{aligned}&|k|\Vert {\textbf{u}}\Vert _{{\textbf{H}}^{m+1}(\varOmega )}+\Vert {\textbf{u}} \Vert _{{\textbf{H}}^{m+1}({\text {curl}},\varOmega )} \le C|k|^{m+1} \Vert {\textbf{u}}\Vert _{{\textbf{H}}^{m+1}({\text {curl}},\varOmega ),k}\nonumber \\&\quad {\mathop {\le }\limits ^{\text {Ind. hyp.}}} C|k|^{m-1}T_{2}(m){\mathop {\le }\limits ^{(\text {4.33})}} C|k|^{m-1}T_{1}(m+1). \end{aligned}$$

Hence,

4.35 $$\begin{aligned} |k|^{-(m+2)}R_{m+1}&= |k|^{-(m+2)}\left( |k|^{-1}\Vert {\text {div}}_{\varGamma }\,{\textbf{g}}_{T}\Vert _{{\textbf{H}}^{m+1/2}(\varGamma )} +|k|^{-1}\Vert {\textbf{f}}\Vert _{{\textbf{H}}^{m+1}(\varOmega )}\right. \nonumber \\&\quad \left. +|k|^{-2}\Vert {\textbf{f}}\Vert _{{\textbf{H}}^{m+1} ({\text {div}},\varOmega )}+|k|\Vert {\textbf{u}}\Vert _{{\textbf{H}}^{m+1} (\varOmega )}+\Vert {\textbf{u}}\Vert _{{\textbf{H}}^{m+1}({\text {curl}},\varOmega )}\right) \nonumber \\&{\mathop {\le }\limits ^{(\text {4.34})}} C|k|^{-3}\left( \Vert {\text {div}}_{\varGamma }\,{\textbf{g}}_{T} \Vert _{{\textbf{H}}^{m+1/2}(\varGamma ),k}+\Vert {\textbf{f}}\Vert _{{\textbf{H}} ^{m+1}({\text {div}},\varOmega ),k}+T_{1}(m+1)\right) \nonumber \\&\le C|k|^{-3} T_{1}(m+1) \end{aligned}$$

and therefore by the induction hypothesis and (4.29)

4.36 $$\begin{aligned} \Vert {\textbf{u}}\Vert _{{\textbf{H}}^{m+2}(\varOmega ),k}&\le C\left( \Vert {\textbf{u}}\Vert _{{\textbf{H}}^{m+1}(\varOmega ),k}+|k|^{-(m+2)}|{\textbf{u}} |_{{\textbf{H}}^{m+2}(\varOmega )}\right) \nonumber \\&\overset{\text {ind. hyp., }(\text {4.29})}{\le }{C\left( |k|^{-3}T_{1}(m)+|k|^{-(m+2)}R_{m+1}\right) }\nonumber \\&\overset{(\text {4.35})}{\le }C|k|^{-3}T_{1}(m+1), \end{aligned}$$

which completes the induction step for formula (4.18a).

Again from the definition of $R_{m+1}$, the induction hypothesis, and (4.33), we have

$$\begin{aligned} |k|^{-(m+2)}R_{m+1}&\le C\left[ |k|^{-2}\Vert {\textbf{g}}_{T}\Vert _{{\textbf{H}}^{m+3/2}(\varGamma ),k}+|k|^{-3}\Vert {\textbf{f}}\Vert _{{\textbf{H}} ^{m+1}({\text {div}},\varOmega ),k}+|k|^{-3}T_{2}(m)\right] \\&\le C|k|^{-3}T_{2}(m+1). \end{aligned}$$

The combination of this with (4.36) and (4.33) leads to

$$\begin{aligned}&\Vert {\textbf{u}}\Vert _{{\textbf{H}}^{m+2}({\text {curl}},\varOmega ),k} \le C\left( \left| k\right| \Vert {\textbf{u}}\Vert _{{\textbf{H}}^{m+2} (\varOmega ),k}+|k|^{-(m+2)}|{\text {curl}}\,{\textbf{u}}|_{{\textbf{H}} ^{m+2}(\varOmega )}\right) \\&\quad \overset{(\text {4.36}),(\text {4.31})}{\le }C\left( \left| k\right| ^{-2}T_{2}(m+1)+|k|^{-(m+2)}\left( \Vert {\textbf{g}}_{T}\Vert _{{\textbf{H}}^{m+3/2}(\varGamma )}+|k|R_{m+1}\right) \right) \\&\quad \le C\left( \left| k\right| ^{-2}T_{2}(m+1)+|k|^{-1} \Vert {\textbf{g}}_{T}\Vert _{{\textbf{H}}^{m+3/2}(\varGamma ),k}+\left| k\right| ^{-2}T_{2}(m+1)\right) \\&\quad \le C\left| k\right| ^{-2} T_{2}(m+1), \end{aligned}$$

which completes the induction argument for (4.18b). $\square$

## Regularity Theory for Maxwell’s Equations

In this section, we collect regularity assertions for the Maxwell model problem (2.40). In particular, the case of analytic data studied in Sect. 5.2 will be a building block for the regularity by decomposition studied in Sect. 7.

### Finite Regularity Theory

The difference between Maxwell’s equations with the “good” sign and the time-harmonic Maxwell equations lies in a lower order term. Therefore, higher regularity statements for the solution of Maxwell’s equations can be inferred from those for with the “good” sign, i.e., from Theorem 4.3. The following result makes this precise.

#### Lemma 5.1

Let $\varOmega$ be a bounded Lipschitz domain with simply connected, sufficiently smooth boundary $\varGamma$. Let $m\in {\mathbb {N}}_{0}$. Then there is $C>0$ (depending only on *m* and $\varOmega$) such that for ${\textbf{f}}\in {\textbf{H}}^{m}({\text {div}},\varOmega )$, ${\textbf{g}}_{T}\in {\textbf{H}}_{T}^{m+1/2}(\varGamma )$ the solution ${\textbf{u}}$ of (2.40) (for ${\textbf{j}}:={\textbf{f}}$) satisfies ${\textbf{u}}\in {\textbf{H}}^{m+1}({\text {curl}},\varOmega )$ and

5.1 $$\begin{aligned} \Vert {\textbf{u}}\Vert _{{\textbf{H}}^{m+1}(\varOmega ),k}&\le C\left[ |k|^{-3}\left( \Vert {\textbf{f}}\Vert _{{\textbf{H}}^{m}({\text {div}},\varOmega ),k} +\Vert {\textbf{g}}_{T}\Vert _{{\textbf{H}}^{m-1/2}({\text {div}}_{\varGamma },\varGamma ),k}\right) +\Vert {\textbf{u}}\Vert _{{\textbf{L}}^{2}(\varOmega )}\!\right] , \end{aligned}$$

5.2 $$\begin{aligned} \Vert {\textbf{u}}\Vert _{{\textbf{H}}^{m+1}({\text {curl}},\varOmega ),k}&\le \! C\left[ \left| k\right| ^{-2}\left( \Vert {\textbf{f}}\Vert _{{\textbf{H}} ^{m}({\text {div}},\varOmega ),k}+|k|\Vert {\textbf{g}}_{T}\Vert _{{\textbf{H}} ^{m+1/2}(\varGamma ),k}\right) +\left| k\right| \Vert {\textbf{u}} \Vert _{{\textbf{L}}^{2}(\varOmega )}\!\right] .\! \end{aligned}$$

If Assumption (3.2) holds, then $\Vert {{\textbf {u}}} \Vert _{{{\textbf {L}}}^{2}(\varOmega )}\le C_{{\text {stab}}}|k|^{\theta -1}\left( \Vert {{\textbf {f}}} \Vert _{{{\textbf {L}}}^{2}(\varOmega )}+\Vert {{\textbf {g}}}_{T}\Vert _{{{\textbf {L}}}^{2} (\varGamma )}\right)$. In particular,

5.3 $$\begin{aligned} \Vert {\textbf{u}}\Vert _{{\textbf{H}}^{1}({\text {curl}},\varOmega ),k}&\le C\left| k\right| ^{-2}\Bigl \{\Vert {\text {div}}\,{\textbf{f}} \Vert _{{\textbf{L}}^{2}(\varOmega )}+{(1+C_\mathrm{{stab}})}|k|^{ \theta +2 } \Vert {\textbf{f}}\Vert _{{\textbf{L}}^{2}(\varOmega )} \nonumber \\&\quad +{(1+C_\mathrm{{stab}})}|k|\Vert {\textbf{g}}_{T}\Vert _{{\textbf{H}}^{1/2}(\varGamma )}+(1 + C_\mathrm{{stab}}) |k|^{ \theta +2 } \Vert {\textbf{g}}_{T}\Vert _{{\textbf{L}}^{2}(\varGamma )}\Bigr \}. \end{aligned}$$

#### Proof

The weak solution ${\textbf{u}}$ of (2.40) exists by Proposition 3.1 and depends continuously on the data. In particular, ${\textbf{u}}\in {\textbf{L}}^{2}(\varOmega )$. From the equation ${\mathcal {L}}_{\varOmega ,k}{\textbf{u}}={\textbf{f}}$, we have $-k^{2} {\text {div}}\,{\textbf{u}}={\text {div}}\,{\textbf{f}}$ so that ${\textbf{u}}\in {\textbf{H}}({\text {div}},\varOmega )$. The function ${\textbf{u}}$ solves

5.4 $$\begin{aligned} {\mathcal {L}}_{\varOmega ,{\text {i}}\,k}{\textbf{u}}={\textbf{f}}+2k^{2} {\textbf{u}},\qquad {\mathcal {B}}_{ \varGamma ,k}{\textbf{u}}={\textbf{g}}_{T}. \end{aligned}$$

It is easy to see that Theorem 4.3 is inductively applicable. We get

5.5 $$\begin{aligned}&\Vert {\textbf{u}}\Vert _{{\textbf{H}}^{m+1}(\varOmega ),k} \le C\left( |k|^{-3}\Vert {\textbf{f}}+2k^{2}{\textbf{u}}\Vert _{{\textbf{H}}^{m} ({\text {div}},\varOmega ),k}+|k|^{-3}\Vert {\textbf{g}}_{T}\Vert _{{\textbf{H}} ^{m-1/2}({\text {div}}_{\varGamma },\varGamma ),k}\right) \nonumber \\&\quad \le C\left( |k|^{-3}\Vert {\textbf{f}}\Vert _{{\textbf{H}}^{m} ({\text {div}},\varOmega ),k}+|k|^{-3}\Vert {\textbf{g}}_{T}\Vert _{{\textbf{H}} ^{m-1/2}({\text {div}}_{\varGamma },\varGamma ),k}+\Vert {\textbf{u}}\Vert _{{\textbf{H}}^{m}(\varOmega ),k}\right) . \end{aligned}$$

We may successively insert (5.5) into itself to arrive at (5.1). The statement (5.2) follows from (5.1) and Theorem 4.3 and the observation $\Vert {\textbf{g}}_{T}\Vert _{{\textbf{H}}^{m-1/2}({\text {div}}_{\varGamma },\varGamma ),k}\le C|k|\Vert {\textbf{g}}_{T}\Vert _{{\textbf{H}}^{m+1/2}(\varGamma ),k}$. $\square$

### Analytic Regularity Theory

In this section, we consider the Maxwell problem (2.40), i.e.,

5.6 $$\begin{aligned} {\mathcal {L}}_{\varOmega ,k}{\textbf{E}}={\textbf{f}}\text { in }\varOmega ,\qquad \qquad {\mathcal {B}}_{ \varGamma ,k}{\textbf{E}}={\textbf{g}}_{T}\text { on }\varGamma \end{aligned}$$

with *analytic* data ${\textbf{f}}$ and ${\textbf{g}}_{T}$ and analytic boundary $\varGamma$. We show in Theorem 5.2 that the solution is analytic, making the dependence on *k* explicit. In [40, Appendix A] we generalize the theory in [45, 53] to the case of inhomogeneous boundary data. The key idea there is to reformulate the problem (5.6) as an elliptic system and then to apply the regularity theory for elliptic systems with analytic data to this problem (see [14]). Here, we summarize the main results.

Problem (5.6) can be formulated as an elliptic system for ${\textbf{U}}=\left( {\textbf{E}},{\textbf{H}}\right)$, where ${\textbf{E}}$ is the electric and ${\textbf{H}}:=-\frac{{\text {i}}}{k}{\text {curl}}\,{\textbf{E}}$ the magnetic field:

5.7 $$\begin{aligned} \begin{array}{cclc} L\left( {\textbf{U}}\right) := &{} \left( \begin{array}{c} {\text {curl}}\,{\text {curl}}\,{\textbf{E}}-\nabla {\text {div}}\,{\textbf{E}}\\ {\text {curl}}\,{\text {curl}}\,{\textbf{H}}-\nabla {\text {div}}\,{\textbf{H}} \end{array} \right) &{} ={\textbf{F}}+k^{2}{\textbf{U}} &{} \text {in }\varOmega ,\\ T\left( {\textbf{U}}\right) := &{} {\textbf{H}}\times {\textbf{n}}-{\textbf{E}}_{T} &{} =-\frac{{\text {i}}}{k}{\textbf{g}}_{T} &{} \text {on }\varGamma ,\\ B\left( {\textbf{U}}\right) := &{} \left( \begin{array}{c} {\text {div}}\,{\textbf{E}}\\ {\text {div}}\,{\textbf{H}}\\ \gamma _{T}{\text {curl}}\,{\textbf{H}}+\left( {\text {curl}}\,{\textbf{E}}\right) _{T} \end{array} \right)&=kG{\textbf{U}}+{\textbf{G}}_{\varGamma }&\text {on }\varGamma \end{array} \end{aligned}$$

for

$$\begin{aligned} {\textbf{F}}:=\left( \begin{array}{c} {\textbf{f}}+\frac{1}{k^{2}}\nabla {\text {div}}\,{\textbf{f}}\\ -\frac{{\text {i}}}{k}{\text {curl}}\,{\textbf{f}} \end{array} \right) ,{\quad }G{\textbf{U}}:=\left( \begin{array}{c} 0\\ 0\\ {\text {i}}\,\left( {\textbf{H}}_{T}-\gamma _{T}{\textbf{E}}\right) \end{array} \right) {,\quad }{\textbf{G}}_{\varGamma }:=\left( \begin{array}{c} -\frac{1}{k^{2}}\left. \left( {\text {div}}\,{\textbf{f}}\right) \right| _{\varGamma }\\ 0\\ -\frac{{\text {i}}}{k}\gamma _{T}{\textbf{f}} \end{array} \right) . \end{aligned}$$

In [40, Appendix A] we show that this system is elliptic in the sense of [14]. For the special case ${\textbf{G}}_{\varGamma }={\textbf{0}}$ and ${\textbf{g}}_{T}={\textbf{0}}$, the analytic regularity theory for this problem has been developed in [45, 53]. The following Theorem 5.2 generalizes their result to the case of inhomogeneous boundary data ${\textbf{g}}_{T}$, ${\textbf{G}}_{\varGamma }$. To describe the analyticity of ${\textbf{g}}_{T}$ and ${\textbf{G}}_{\varGamma }$, we assume that these functions are restrictions of analytic functions ${\textbf{g}}^{*}$ and ${\textbf{G}}^{*}$ on an open neighborhood ${\mathcal {U}}_{\varGamma }$ of $\varGamma$ and satisfy ${\textbf{g}}_{T}=\gamma {\textbf{g}}^{*}$ and ${\textbf{G}}_{\varGamma } =\gamma {\textbf{G}}^{*}$ for the standard trace operator $\gamma$, i.e., the restriction to $\varGamma$. We write ${\textbf{g}}_{T}\in {\mathcal {A}}(C_{{\textbf{g}}},\lambda _{{\textbf{g}} },{\mathcal {U}}_{\varGamma }\cap \varOmega )$ if ${\textbf{g}}^{*}\in {\mathcal {A}} (C_{{\textbf{g}}},\lambda _{{\textbf{g}}},{\mathcal {U}}_{\varGamma }\cap \varOmega )$.

#### Theorem 5.2

Let $\varOmega \subset {{\mathbb {R}}}^{3}$ be a bounded Lipschitz domain with a simply connected, analytic boundary. Let ${\mathcal {U}}_{\varGamma }$ be an open neighborhood of $\varGamma$. Let ${\textbf{f}}\in {\mathcal {A}} (C_{{\textbf{f}}},\lambda _{{\textbf{f}}},\varOmega )$ and ${\textbf{g}}_{T} \in {\mathcal {A}}(C_{{\textbf{g}}},\lambda _{{\textbf{g}}},{\mathcal {U}}_{\varGamma } \cap \varOmega )$. Then there are constants *B*, $C>0$ (depending only on $\varOmega$, ${\mathcal {U}}_{\varGamma }$, and $\lambda _{{\textbf{f}}}$, $\lambda _{{\textbf{g}}}$) such that the solution ${\textbf{E}}$ of (5.6) satisfies

5.8 $$\begin{aligned} {\textbf{E}}\in {\mathcal {A}}(CC_{{\textbf{E}}},B,\varOmega ), \end{aligned}$$

where $C_{{\textbf{E}}}=C_{{\textbf{f}}}\left| k\right| ^{-2} +C_{{{\textbf{g}}}}\left| k\right| ^{-1}+\frac{1}{\left| k\right| }\left\| {{\textbf{E}}}\right\| _{{\textbf{H}}^{1}\left( {\text {curl}},\varOmega \right) ,k}$. If Assumption (3.2) holds, then

5.9 $$\begin{aligned} C_{{\textbf{E}}}\le C{(1+C_\mathrm{{stab}})}\left( C_{{\textbf{f}}}|k|^{ \theta -1 } +C_{{\textbf{g}}}|k|^{ \theta -1/2} \right) . \end{aligned}$$

#### Proof

The statement of the theorem follows from [40, Cor. A.2] and more details can be found there. The existence ${\textbf{u}}\in {\textbf{X}}_{{\text {imp}}}$ is implied by Proposition 3.1, and finite regularity assertions for ${\textbf{E}}$ are provided in Lemma 5.1. In particular, ${\textbf{E}}\in {\textbf{H}} ^{2}(\varOmega )$. In turn, ${\textbf{U}}=({\textbf{E}},{\textbf{H}})\in {\textbf{H}} ^{1}({\text {curl}},\varOmega )$ solves the elliptic system (5.7). This makes [40, Thm. A.1] applicable, which shows the corresponding result for ${\textbf{U}}$ by a boot-strapping argument and an explicit tracking of the wavenumber *k* to arrive at the result of [40, Cor. A.2]

5.10 $$\begin{aligned} |{{\textbf{E}}}|_{{\textbf{H}}^{p}\left( \varOmega \right) }\le CC_{{\textbf{E}} }B^{p}\max (p,|k|)^{p}\quad \forall p\in {\mathbb {N}}_{\ge 2} \end{aligned}$$

with $C_{{\textbf{E}}}$ as given in the statement. A direct calculation shows $\Vert {\textbf{E}}\Vert _{{\textbf{H}}^{1}(\varOmega )}\le C_{{\textbf{E}}} |k|$ and $\Vert {\textbf{E}}\Vert _{{\textbf{L}}^{2}(\varOmega )}\le C_{{\textbf{E}}}$ so that (5.10) also holds for $p=0$ and $p=1$. This shows (5.8).

The estimate (5.9) follows from (5.3) of Lemma 5.1 and the definition of the analyticity classes together with the trace estimates $\Vert {\textbf{g}}_{T}\Vert _{{\textbf{H}}^{1/2}(\varGamma )} \le C C_{{\textbf{g}}} |k|$ and $\Vert {\textbf{g}}_{T}\Vert _{{\textbf{L}}^{2}(\varGamma )} \le C C_{{\textbf{g}}} |k|^{1/2}$. $\square$

## Frequency Splittings

As in [17, 31, 34, 37–39] we analyze the regularity of Maxwell’s equations (2.40) via a decomposition of the right-hand side into high and low frequency parts.

### Frequency Splittings in $\varOmega$: $H_{{\mathbb {R}}^{3}}$, $L_{{\mathbb {R}}^{3}}$, $H_{\varOmega }$, $L_{\varOmega }$, ${H}_{\varOmega }^{0}$, ${L}_{\varOmega }^{0}$

In order to construct the splitting, we start by recalling the definition of the Fourier transform for sufficiently smooth functions with compact support

6.1 $$\begin{aligned} {\hat{u}}( {\varvec{\xi }}) ={\mathcal {F}}( u) ( {\varvec{\xi }} ) =\left( 2\pi \right) ^{-3/2} {\displaystyle \int _{{\mathbb {R}}^{3}}} {\text {e}}^{-{\text {i}}\,\langle {\varvec{\xi }},{\textbf{x}}\rangle }u( {\textbf{x}}) d{\textbf{x}}\qquad \forall {\varvec{\xi }} \in {\mathbb {R}}^{3} \end{aligned}$$

and the inversion formula

$$\begin{aligned} u( {\textbf{x}}) ={\mathcal {F}}^{-1}( u)( {\textbf{x}}) =\left( 2\pi \right) ^{-3/2} {\displaystyle \int _{{\mathbb {R}}^{3}}} {\text {e}}^{{\text {i}}\,\langle {\textbf{x}}, {\varvec{\xi }} \rangle }{\hat{u}}( {\varvec{\xi }}) d {\varvec{\xi }} \qquad \forall {\textbf{x}}\in {\mathbb {R}}^{3}. \end{aligned}$$

These formulas extend to tempered distributions and in particular to functions in $L^{2}({\mathbb {R}}^{3})$. Next, we introduce a frequency splitting for functions in ${\mathbb {R}}^{3}$ that depends on *k* and a parameter $\lambda >1$ by using the Fourier transformation. The low and high frequency part is given by

6.2 $$\begin{aligned} L_{{\mathbb {R}}^{3}}u:={\mathcal {F}}^{-1}\left( \chi _{\lambda \left| k\right| }{\mathcal {F}}( u) \right) \quad \text {and}\quad H_{{\mathbb {R}}^{3}}u:={\mathcal {F}}^{-1}\left( \left( 1-\chi _{\lambda \left| k\right| }\right) {\mathcal {F}}( u) \right) , \end{aligned}$$

where $\chi _{\delta }$ is the characteristic function of the open ball with radius $\delta >0$ centered at the origin. We note the splitting $H_{{\mathbb {R}} ^{3}}+L_{{\mathbb {R}}^{3}}=I$. By using Stein’s extension operator ${\mathcal {E}}_{{\text {Stein}}}$, [52, Chap. VI] this splitting induces a frequency splitting for functions in Sobolev spaces in $\varOmega$ via

6.3 $$\begin{aligned} L_{\varOmega }{\textbf{f}}:=\left. \left( L_{{\mathbb {R}}^{3}}{\mathcal {E}} _{{\text {Stein}}}{\textbf{f}}\right) \right| _{\varOmega } \quad \text {and}\quad H_{\varOmega }{\textbf{f}}:=\left. \left( H_{{\mathbb {R}}^{3} }{\mathcal {E}}_{{\text {Stein}}}{\textbf{f}}\right) \right| _{\varOmega }, \end{aligned}$$

where, again, $L_{\varOmega }{\textbf{f}}+H_{\varOmega }{\textbf{f}}={\textbf{f}}$ in $\varOmega$.

In general, the condition ${\text {div}}\,{\textbf{f}}=0$ neither implies ${\text {div}}L_{\varOmega }{\textbf{f}}=0$ nor ${\text {div}}H_{\varOmega }{\textbf{f}}=0$. We therefore introduce another lifting (instead of ${\mathcal {E}}_{{\text {Stein}}}$) for functions in Sobolev spaces on $\varOmega$ that passes on the divergence-free property to the lifting to the full space and allows for alternative frequency splittings $L_{\varOmega }^{0}$, $H_{\varOmega }^{0}$ at the expense that $L_{\varOmega }^{0}+H_{\varOmega }^{0}$ is not the identity but the *identity plus a smoothing operator*. With the operator ${\textbf{R}}_{2}$ of Lemma 2.6, which has been constructed in [15], we set

6.4 $$\begin{aligned} H_{\varOmega }^{0}{\textbf{f}}:={\text {curl}}\,H_{{\mathbb {R}}^{3}} {\mathcal {E}}_{{\text {Stein}}}{\textbf{R}}_{2}{\textbf{f}}\quad \text {and}\quad L_{\varOmega }^{0}{\textbf{f}}:={\text {curl}}\,L_{{\mathbb {R}} ^{3}}{\mathcal {E}}_{{\text {Stein}}}{\textbf{R}}_{2}{\textbf{f}} \end{aligned}$$

and define the operator ${\textbf{S}}$ by

6.5 $$\begin{aligned} {\textbf{S}}{\textbf{f}}:\mathbf {=f}-\left. \left( H_{\varOmega }^{0}{\textbf{f}} +L_{\varOmega }^{0}{\textbf{f}}\right) \right| _{\varOmega }\mathbf {.} \end{aligned}$$

In view of (2.24), we have for ${\textbf{f}}$ with ${\text {div}}\,{\textbf{f}}=0$ that ${\textbf{S}}{\textbf{f}}={\textbf{K}}_{2}{\textbf{f}}|_{\varOmega }$ so that in particular for all *s*, $s^{\prime }$

6.6 $$\begin{aligned} \Vert {\textbf{S}}{\textbf{f}}\Vert _{{\textbf{H}}^{s}(\varOmega )}\le C_{s,s^{\prime } }\Vert {\textbf{f}}\Vert _{{\textbf{H}}^{s^{\prime }}(\varOmega )}\qquad \forall {\textbf{f}}\in {\textbf{H}}^{s^{\prime }}(\varOmega ):{\text {div}}\,{\textbf{f}}=0. \end{aligned}$$

### Frequency Splittings on $\varGamma$

For the definition of the Hodge decompositions and frequency splittings of this section, we recall that $\varOmega$ has a simply connected, analytic boundary.

#### Remark 6.1

The Laplace–Beltrami operator $\varDelta _{\varGamma }$ is self-adjoint with respect to the $L^{2}(\varGamma )$ scalar product $\left( \cdot ,\cdot \right) _{L^{2}(\varGamma )}$ and positive semidefinite. It admits a countable sequence of eigenfunctions in $L^{2}(\varGamma )$ denoted by $Y_{\ell }^{m}$ such that

6.7 $$\begin{aligned} -\varDelta _{\varGamma }Y_{\ell }^{m}=\lambda _{\ell }Y_{\ell }^{m}\qquad \text { for } \ell =0,1,\ldots \text {and }m\in \iota _{\ell }. \end{aligned}$$

Here, $\iota _{\ell }$ is a finite index set whose cardinality equals the multiplicity of the eigenvalue $\lambda _{\ell }$, and we always assume that the eigenvalues $\lambda _{\ell }$ are distinct and ordered increasingly. We have $\lambda _{0}=0$ and for $\ell \ge 1$, they are real and positive and accumulate at infinity. Since we assumed that $\varGamma$ is simply connected we know that $\lambda _{0}=0$ is a simple eigenvalue. $\square$

According to [43, Sec. 5.4.1], any tangential field ${\textbf{h}}_{T}\in {\textbf{L}}_{T}^{2}(\varGamma )$ on the bounded, simply connected manifold $\varGamma$ admits an expansion

6.8 $$\begin{aligned} {\textbf{h}}_{T}=\sum _{\ell =1}^{\infty }\sum _{m\in \iota _{\ell }} \alpha _{\ell }^{m}\nabla _{\varGamma }Y_{\ell }^{m}+\beta _{\ell }^{m}\left( \overrightarrow{\mathop {{\text {curl}}}\nolimits _{\varGamma }}\,Y_{\ell }^{m} \right) . \end{aligned}$$

The functions $\left\{ \nabla _{\varGamma }Y_{\ell }^{m},\overrightarrow{\mathop {{\text {curl}}}\nolimits _{\varGamma }}\,Y_{\ell }^{m}:\ell \in {\mathbb {N}}_{\ge 1},\,m\in \iota _{\ell }\right\}$ constitute an orthogonal basis of ${\textbf{L}}_{T}^{2}(\varGamma )$ and hence the coefficients $\alpha _{\ell }^{m}$, $\beta _{\ell }^{m}$ are uniquely determined via (6.8). We set

6.9 $$\begin{aligned} \begin{array}{ll} {\mathcal {L}}_{{\text {imp}}}^{\nabla }{\textbf{h}}_{T}:= {\displaystyle \sum \limits _{\ell =1}^{\infty }} {\displaystyle \sum \limits _{m\in \iota _{\ell }}} \alpha _{\ell }^{m}Y_{\ell }^{m}, &{} \qquad {\mathcal {L}}_{{\text {imp}} }^{{\text {curl}}}{\textbf{h}}_{T}:= {\displaystyle \sum \limits _{\ell =1}^{\infty }} {\displaystyle \sum \limits _{m\in \iota _{\ell }}} \beta _{\ell }^{m}Y_{\ell }^{m},\\ \varPi _{{\text {imp}}}^{\nabla }:=\nabla _{\varGamma }{\mathcal {L}} _{{\text {imp}}}^{\nabla }, &{}\qquad \varPi _{{\text {imp}}} ^{{\text {curl}}}:=I-\varPi _{{\text {imp}}}^{\nabla }=\overrightarrow{\mathop {{\text {curl}}}\nolimits _{\varGamma }}\,{\mathcal {L}}_{{\text {imp}} }^{{\text {curl}}}, \end{array} \end{aligned}$$

where *I* denotes the identity operator.

#### Remark 6.2

${\mathcal {L}}_{{\text {imp}}}^{\nabla }{\textbf{h}}_{T}$ and ${\mathcal {L}}_{{\text {imp}}}^{{\text {curl}} }{\textbf{h}}_{T}$ are characterized by

6.10 $$\begin{aligned} \left( \nabla _{\varGamma }{\mathcal {L}}_{{\text {imp}}}^{\nabla }{\textbf{h}} _{T},\nabla _{\varGamma }\psi \right) _{{\textbf{L}}^{2}( \varGamma ) }&=\left( {\textbf{h}}_{T},\nabla _{\varGamma }\psi \right) _{{\textbf{L}}^{2}( \varGamma ) }\quad \forall \psi \in C^{\infty }(\varGamma ), \end{aligned}$$

6.11 $$\begin{aligned} \left( \overrightarrow{{\text {curl}}_{\varGamma }}\,{\mathcal {L}} _{{\text {imp}}}^{{\text {curl}}}{\textbf{h}}_{T},\overrightarrow{{\text {curl}}_{\varGamma }}\psi \right) _{{\textbf{L}}^{2}( \varGamma ) }&=\left( {\textbf{h}}_{T},\overrightarrow{{\text {curl}}_{\varGamma }}\psi \right) _{{\textbf{L}}^{2}( \varGamma ) }\quad \forall \psi \in C^{\infty }(\varGamma ), \end{aligned}$$

and the conditions $({\mathcal {L}}_{{\text {imp}}}^{\nabla }{\textbf{h}} _{T},1)_{ L^{2} (\varGamma )}=0$ and $({\mathcal {L}}_{{\text {imp}}}^{{\text {curl}} }{\textbf{h}}_{T},1)_{ L^{2} (\varGamma )}=0$. In strong form, we have in view of ${\text {curl}}_{\varGamma }\overrightarrow{{\text {curl}}_{\varGamma }}=-\varDelta _{\varGamma }$ that $\varDelta _{\varGamma }{\mathcal {L}}_{{\text {imp}}}^{\nabla }{\textbf{h}} _{T}={\text {div}}_{\varGamma }{\textbf{h}}_{T}$ and $\varDelta _{\varGamma }{\mathcal {L}}_{{\text {imp}}}^{{\text {curl}}}{\textbf{h}} _{T}=-{\text {curl}}_{\varGamma }{\textbf{h}}_{T}$. $\square$

In summary, we have introduced a Hodge decomposition:

6.12 $$\begin{aligned} {\textbf{h}}_{T}= & {} \varPi _{{\text {imp}}}^{\nabla }{\textbf{h}}_{T}+\varPi _{{\text {imp}}}^{{\text {curl}}}{\textbf{h}}_{T}=\nabla _{\varGamma }\varphi +\overrightarrow{\mathop {{\text {curl}}}\nolimits _{\varGamma }}\,\psi \nonumber \\{} & {} \quad \text {for }\varphi ={\mathcal {L}}_{{\text {imp}}}^{\nabla } {\textbf{h}}_{T}\text { and }\psi ={\mathcal {L}}_{{\text {imp}}} ^{{\text {curl}}}{\textbf{h}}_{T} \end{aligned}$$

(for further details see [43, Sec. 5.4.1]).

Next, we introduce the harmonic extension ${\mathcal {E}}_{\varOmega }^{\varDelta }:H^{1/2}\left( \varGamma \right) \rightarrow H^{1}\left( \varOmega \right)$ of Dirichlet boundary data defined by

6.13 $$\begin{aligned} \varDelta \left( {\mathcal {E}}_{\varOmega }^{\varDelta }\varphi \right) =0\quad \text {in }\varOmega {, \qquad }\left. {\mathcal {E}}_{\varOmega }^{\varDelta }\varphi \right| _{\varGamma }=\varphi . \end{aligned}$$

(Later, we will use that ${\mathcal {E}}_{\varOmega }^{\varDelta }$ extends to a continuous operator $H^{s}(\varGamma )\rightarrow H^{1/2+s}(\varOmega )$ for $s\ge 0$.) This allows us to define boundary frequency filters $L_{ \varGamma }$ and $H_{ \varGamma }$ based on this Dirichlet lifting by

6.14 $$\begin{aligned} L_{ \varGamma }\varphi :=\left. \left( L_{\varOmega }{\mathcal {E}}_{\varOmega }^{\varDelta } \varphi \right) \right| _{\varGamma }\quad \text {and}\quad H_{ \varGamma }\varphi :=\left. \left( H_{\varOmega }{\mathcal {E}}_{\varOmega }^{\varDelta } \varphi \right) \right| _{\varGamma }. \end{aligned}$$

The vector-valued versions for tangential fields on the surface are used to define

6.15 $$\begin{aligned} \begin{array}{ll} {\textbf{H}}_{\varGamma }^{\nabla }\left( {\textbf{h}}_{T}\right) :=\nabla _{\varGamma }\left( H_{ \varGamma }{\mathcal {L}}_{{\text {imp}}}^{\nabla }{\textbf{h}}_{T}\right) , &{} {\textbf{L}}_{\varGamma }^{\nabla }\left( {\textbf{h}}_{T}\right) :=\nabla _{\varGamma }\left( L_{ \varGamma }{\mathcal {L}}_{{\text {imp}}}^{\nabla }{\textbf{h}}_{T}\right) ,\\ {\textbf{H}}_{\varGamma }^{{\text {curl}}}\left( {\textbf{h}}_{T}\right) :=\overrightarrow{\mathop {{\text {curl}}}\nolimits _{\varGamma }}\,\left( H_{ \varGamma }{\mathcal {L}}_{{\text {imp}}}^{{\text {curl}}}{\textbf{h}}_{T}\right) , &{} {\textbf{L}}_{\varGamma }^{{\text {curl}}}\left( {\textbf{h}}_{T}\right) :=\overrightarrow{\mathop {{\text {curl}}}\nolimits _{\varGamma }}\,\left( L_{ \varGamma }{\mathcal {L}}_{{\text {imp}}}^{{\text {curl}}}{\textbf{h}}_{T}\right) , \end{array} \end{aligned}$$

and we set

6.16 $$\begin{aligned} {\textbf{H}}_{\varGamma }:={\textbf{H}}_{\varGamma }^{\nabla }+{\textbf{H}}_{\varGamma }^{{\text {curl}}}\quad \text {and}\quad {\textbf{L}}_{\varGamma }:={\textbf{L}} _{\varGamma }^{\nabla }+{\textbf{L}}_{\varGamma }^{{\text {curl}}}. \end{aligned}$$

### Estimates for the Frequency Splittings

#### Lemma 6.3

Let $\varOmega$ be a bounded Lipschitz domain with simply connected, analytic boundary. The operators ${\mathcal {L}}_{{\text {imp}} }^{\nabla }$ and ${\mathcal {L}}_{{\text {imp}}}^{{\text {curl}}}$ can be extended (uniquely) to bounded linear operators ${\textbf{H}}_{T}^{s} (\varGamma )\rightarrow H^{s+1}(\varGamma )$ for any $s\in {\mathbb {R}}$ and

6.17a $$\begin{aligned} \Vert {\mathcal {L}}_{{\text {imp}}}^{\nabla }{\textbf{h}}_{T}\Vert _{H^{s+1}(\varGamma )}&\le C_{s}\Vert {\text {div}}_{\varGamma }\,{\textbf{h}} _{T}\Vert _{H^{s-1}(\varGamma )}, \end{aligned}$$

6.17b $$\begin{aligned} \Vert {\mathcal {L}}_{{\text {imp}} }^{{\text {curl}}}{\textbf{h}}_{T}\Vert _{H^{s+1}(\varGamma )}&\le C_{s} \Vert {\text {curl}}_{\varGamma }\,{\textbf{h}}_{T}\Vert _{H^{s-1}(\varGamma )}. \end{aligned}$$

For every $s>-1$, there is $C_{s}>0$ independent of $\lambda >1$ (appearing in (6.2)) such that for any ${\textbf{h}}_{T}\in {\textbf{H}}_{T}^{s}(\varGamma )$ there holds ${\textbf{H}}_{\varGamma } {\textbf{h}}_{T}={\textbf{H}}_{\varGamma }^{\nabla }{\textbf{h}}_{T}+{\textbf{H}}_{\varGamma }^{{\text {curl}}}{\textbf{h}}_{T}$ together with

$$\begin{aligned} \left\| {\textbf{H}}_{\varGamma }^{\nabla }{\textbf{h}}_{T}\right\| _{{\textbf{H}}^{s}(\varGamma )}+\left\| {\textbf{H}}_{\varGamma }^{{\text {curl}} }{\textbf{h}}_{T}\right\| _{{\textbf{H}}^{s}\left( \varGamma \right) }\le C_{ s }\left\| {\textbf{h}}_{T}\right\| _{{\textbf{H}}^{s}\left( \varGamma \right) }. \end{aligned}$$

#### Proof

The mapping properties for ${\mathcal {L}}_{{\text {imp}}}^{\nabla }$, ${\mathcal {L}}_{{\text {imp}}}^{{\text {curl}}}$, follow directly from elliptic regularity theory on smooth manifolds in view of Remark 6.2. For the stability of the operators ${\textbf{H}}_{\varGamma }^{\nabla }$, ${\textbf{H}}_{\varGamma }^{{\text {curl}}}$ we use the stability of the operator $H_{\varOmega }:H^{s^{\prime }}(\varOmega )\rightarrow H^{s^{\prime }}(\varOmega )$ for $s^{\prime }\ge 0$ and the stability of the trace operator $\gamma :H^{1/2+s^{\prime }}(\varOmega )\rightarrow H^{s^{\prime }}(\varGamma )$ for $s^{\prime }>0$ as in [38, Lem. 4.2] to get that ${\textbf{h}}_{T}\mapsto \gamma H_{\varOmega }{\mathcal {E}}_{\varOmega }^{\varDelta }{\mathcal {L}}_{{\text {imp}}}^{\nabla }{\textbf{h}}_{T}$ maps continuously $H^{-1+\varepsilon }(\varGamma )\rightarrow H^{\varepsilon }(\varGamma )$ for any $\varepsilon >0$ with continuity constant independent of $\lambda >1$. Since $\nabla _{\varGamma }:H^{\varepsilon } (\varGamma )\rightarrow {\textbf{H}}_{T}^{-1+\varepsilon }(\varGamma )$, the result follows. The case of ${\textbf{H}}_{\varGamma }^{{\text {curl}}}$ is handled analogously. $\square$

We recall some properties of the high frequency splittings that are proved in [38, Lem. 4.2].

#### Proposition 6.4

Let $\lambda >1$ be the parameter appearing in the definition of $H_{{\mathbb {R}}^{3}}$ in (6.2). Let $H_{\varOmega }$, $H_{\varOmega }^{0}$, and ${\textbf{H}}_{\varGamma }$ be the operators of (6.3), (6.4), and (6.16). There are constants $C_{s^{\prime },s}$ independent of $\lambda >1$ such that the following holds.

(i) The frequency splitting (6.2) satisfies for all $0\le s^{\prime }\le s$ the estimates
  6.18 $$\begin{aligned} \left\| H_{{\mathbb {R}}^{3}}f\right\| _{H^{s^{\prime }}({\mathbb {R}}^{3})}&\le C_{s^{\prime },s}\left( \lambda \left| k\right| \right) ^{s^{\prime }-s}\left\| f\right\| _{H^{s}({\mathbb {R}}^{3})}\qquad \forall f\in H^{s}({\mathbb {R}}^{3}), \end{aligned}$$
  6.19 $$\begin{aligned} \left\| H_{\varOmega }f\right\| _{H^{s^{\prime }}(\varOmega )}&\le C_{s^{\prime },s}\left( \lambda \left| k\right| \right) ^{s^{\prime }-s}\left\| f\right\| _{H^{s}(\varOmega )}\qquad \forall f\in H^{s} (\varOmega ), \end{aligned}$$
  6.20 $$\begin{aligned} \left\| H_{\varOmega }^{0}{\textbf{f}}\right\| _{{\textbf{H}}^{s^{\prime } }(\varOmega )}&\le C_{s^{\prime },s}\left( \lambda \left| k\right| \right) ^{s^{\prime }-s}\left\| {\textbf{f}}\right\| _{{\textbf{H}} ^{s}(\varOmega )}\qquad \forall {\textbf{f}}\in {\textbf{H}}^{s}(\varOmega ). \end{aligned}$$
  These estimates hold also for Lipschitz domains.
(ii) Let $0\le s^{\prime }<s$ or $0<s^{\prime }\le s$. Then the operator $H_{ \varGamma }$ satisfies
  6.21 $$\begin{aligned} \left\| H_{ \varGamma }g\right\| _{H^{s^{\prime }}( \varGamma )}\le C_{s^{\prime },s}\left( \lambda \left| k\right| \right) ^{s^{\prime }-s}\Vert g\Vert _{H^{s}( \varGamma )}. \end{aligned}$$
(iii) Let $-1\le s^{\prime }<s$ or $-1<s^{\prime }\le s$. Then the operator ${\textbf{H}}_{\varGamma }$ satisfies for ${\textbf{g}}_{T}\in {\textbf{H}}_{T}^{s}(\varGamma )$
  6.22a $$\begin{aligned} \left\| {\textbf{H}}_{\varGamma }{\textbf{g}}_{T}\right\| _{{\textbf{H}} ^{s^{\prime }}(\varGamma )}&\le C_{s^{\prime },s}\left( \lambda \left| k\right| \right) ^{s^{\prime }-s}\Vert {\textbf{g}}_{T}\Vert _{{\textbf{H}} ^{s}(\varGamma )}, \end{aligned}$$
  6.22b $$\begin{aligned} \left\| \mathop {{\text {div}}}\nolimits _{\varGamma }\,{\textbf{H}}_{\varGamma } {\textbf{g}}_{T}\right\| _{H^{s^{\prime }-1}(\varGamma )}&\le C_{s^{\prime },s}\left( \lambda \left| k\right| \right) ^{s^{\prime }-s} \Vert \mathop {{\text {div}}}\nolimits _{\varGamma }\,{\textbf{g}}_{T}\Vert _{{\textbf{H}} ^{s-1}(\varGamma )}, \end{aligned}$$
  6.22c $$\begin{aligned} \left\| \mathop {{\text {curl}}}\nolimits _{\varGamma }\,{\textbf{H}}_{\varGamma } {\textbf{g}}_{T}\right\| _{H^{s^{\prime }-1}(\varGamma )}&\le C_{s^{\prime },s}\left( \lambda \left| k\right| \right) ^{s^{\prime }-s} \Vert \mathop {{\text {curl}}}\nolimits _{\varGamma }\,{\textbf{g}}_{T}\Vert _{{\textbf{H}} ^{s-1}(\varGamma )}. \end{aligned}$$

#### Proof

*Proof of* (i): Estimates (6.18) and (6.19) are shown in [38, Lem. 4.2]. To see (6.20), we bound $H_{\varOmega }^{0}$ as follows

$$\begin{aligned} \left\| H_{\varOmega }^{0}{\textbf{f}}\right\| _{{\textbf{H}}^{s^{\prime } }(\varOmega )}&=\left\| {\text {curl}}\,H_{{\mathbb {R}}^{3}} {\mathcal {E}}_{{\text {Stein}}}{\textbf{R}}_{2}{\textbf{f}}\right\| _{{\textbf{H}}^{s^{\prime }}(\varOmega )}\le \left\| H_{{\mathbb {R}}^{3}} {\text {curl}}\,{\mathcal {E}}_{{\text {Stein}}}{\textbf{R}} _{2}{\textbf{f}}\right\| _{{\textbf{H}}^{s^{\prime }}( {\mathbb {R}}^{3} )}\\&\overset{(\text {6.18})}{\le }C_{s^{\prime },s}\left( \lambda \left| k\right| \right) ^{s^{\prime }-s}\left\| {\text {curl}}\,{\mathcal {E}}_{{\text {Stein}}}{\textbf{R}} _{2}{\textbf{f}}\right\| _{{\textbf{H}}^{s}({\mathbb {R}}^{3})}\\&\le C_{s^{\prime },s}\left( \lambda \left| k\right| \right) ^{s^{\prime }-s}\left\| {\mathcal {E}}_{{\text {Stein}}}{\textbf{R}} _{2}{\textbf{f}}\right\| _{{\textbf{H}}^{s+1}({\mathbb {R}}^{3})} \\&\le C_{s^{\prime },s}\left( \lambda \left| k\right| \right) ^{s^{\prime }-s}\left\| {\textbf{R}}_{2}{\textbf{f}}\right\| _{{\textbf{H}}^{s+1}(\varOmega )} \le C_{s^{\prime },s}\left( \lambda \left| k\right| \right) ^{s^{\prime }-s}\left\| {\textbf{f}}\right\| _{{\textbf{H}}^{s}(\varOmega )}. \end{aligned}$$

*Proof of* (ii): For $s^{\prime }>0$ the definition of $H_{ \varGamma }$ in (6.14) implies

$$\begin{aligned} \left\| H_{ \varGamma }g\right\| _{H^{s^{\prime }}( \varGamma )}&=\left\| H_{\varOmega }{\mathcal {E}}_{\varOmega }^{\varDelta }g\right\| _{H^{s^{\prime }}( \varGamma )}\le C\left\| H_{\varOmega }{\mathcal {E}}_{\varOmega }^{\varDelta }g\right\| _{H^{s^{\prime }+1/2}(\varOmega )} \\&\overset{(\text {6.19})}{\le }\tilde{C}_{s^{\prime },s}\left( \lambda \left| k\right| \right) ^{s^{\prime }-s}\left\| {\mathcal {E}}_{\varOmega }^{\varDelta }g\right\| _{H^{s+1/2}(\varOmega )}. \end{aligned}$$

The regularity theory for the Laplace problem (6.13) leads to (6.21). For the case $s^{\prime }=0$, we have $s>0$, and the multiplicative trace inequality,

$$\begin{aligned} \Vert H_{ \varGamma }g\Vert _{L^{2}( \varGamma )}\le C\Vert H_{\varOmega }{\mathcal {E}}_{\varOmega }^{\varDelta }g\Vert _{L^{2}(\varOmega )}^{1-1/(2s+1)}\Vert H_{\varOmega }{\mathcal {E}}_{\varOmega }^{\varDelta }g\Vert _{H^{s+1/2}(\varOmega )}^{1/(2s+1)}, \end{aligned}$$

together with the properties of $H_{\varOmega }$ lead to the result.

*Proof of* (iii): We have $\displaystyle {\textbf{H}}_{\varGamma }{\textbf{g}}_{T}=\nabla _{\varGamma }( H_{ \varGamma }{\mathcal {L}}_{{\text {imp}}}^{\nabla }{\textbf{g}}_{T}) +\overrightarrow{\mathop {{\text {curl}}}\nolimits _{\varGamma }}\,( H_{ \varGamma }{\mathcal {L}}_{{\text {imp}}}^{{\text {curl}}}{\textbf{g}} _{T}).$ A triangle inequality leads to

$$\begin{aligned} \left\| {\textbf{H}}_{\varGamma }{\textbf{g}}_{T}\right\| _{{\textbf{H}} ^{s^{\prime }}\left( \varGamma \right) }&\le \left\| H_{ \varGamma }{\mathcal {L}}_{{\text {imp}}}^{\nabla }{\textbf{g}}_{T}\right\| _{{\textbf{H}}^{s^{\prime }+1}\left( \varGamma \right) }+\left\| H_{ \varGamma }{\mathcal {L}}_{{\text {imp}}}^{{\text {curl}}}{\textbf{g}} _{T}\right\| _{{\textbf{H}}^{s^{\prime }+1}\left( \varGamma \right) }\\&\overset{(\text {6.21})}{\le }C_{s^{\prime },s}\left( \lambda \left| k\right| \right) ^{s^{\prime }-s}\left( \left\| {\mathcal {L}}_{{\text {imp}}}^{\nabla } {\textbf{g}}_{T}\right\| _{{\textbf{H}}^{s+1}\left( \varGamma \right) }+\left\| {\mathcal {L}}_{{\text {imp}}}^{{\text {curl}} }{\textbf{g}}_{T}\right\| _{{\textbf{H}}^{s+1}\left( \varGamma \right) }\right) \\&\overset{(\text {6.17})}{\le }C_{s^{\prime },s}\left( \lambda \left| k\right| \right) ^{s^{\prime }-s}\left\| {\textbf{g}}_{T}\right\| _{{\textbf{H}}^{s}\left( \varGamma \right) }, \end{aligned}$$

which shows (6.22a). For (6.22b) we start from

$$\begin{aligned} \mathop {{\text {div}}}\nolimits _{\varGamma }\,{\textbf{H}}_{\varGamma }{\textbf{g}} _{T}=\mathop {{\text {div}}}\nolimits _{\varGamma }\,\left( \nabla _{\varGamma }( H_{ \varGamma }{\mathcal {L}}_{{\text {imp}}}^{\nabla }{\textbf{g}}_{T}) +\overrightarrow{\mathop {{\text {curl}}}\nolimits _{\varGamma }}\,( H_{ \varGamma }{\mathcal {L}}_{{\text {imp}}}^{{\text {curl}}}{\textbf{g}} _{T}) \right) =\varDelta _{\varGamma }( H_{ \varGamma }{\mathcal {L}}_{{\text {imp}}}^{\nabla }{\textbf{g}}_{T}). \end{aligned}$$

We apply the previous estimate (6.21) to get

$$\begin{aligned} \left\| \mathop {{\text {div}}}\nolimits _{\varGamma }\,{\textbf{H}}_{\varGamma } {\textbf{g}}_{T}\right\| _{H^{s^{\prime }-1}\left( \varGamma \right) }&=\left\| \varDelta _{\varGamma }( H_{ \varGamma }{\mathcal {L}}_{{\text {imp}}}^{\nabla }{\textbf{g}}_{T}) \right\| _{H^{s^{\prime }-1}\left( \varGamma \right) }\le C\left\| H_{ \varGamma }{\mathcal {L}}_{{\text {imp}}}^{\nabla }{\textbf{g}}_{T}\right\| _{H^{s^{\prime }+1}\left( \varGamma \right) }\\&\le C_{s^{\prime },s}\left( \lambda \left| k\right| \right) ^{s^{\prime }-s}\left\| {\mathcal {L}}_{{\text {imp}}}^{\nabla } {\textbf{g}}_{T}\right\| _{H^{s+1}\left( \varGamma \right) }. \end{aligned}$$

Since $\left\| {\mathcal {L}}_{{\text{ imp }} }^{\nabla }{{\textbf {g}}}_{T}\right\| _{H^{s+1}\left( \varGamma \right) }\le {C} \left\| \varDelta _{\varGamma }{\mathcal {L}}_{{\text{ imp }} }^{\nabla }{{\textbf {g}}}_{T}\right\| _{H^{s-1}\left( \varGamma \right) }$ and from (6.10) we obtain $\varDelta _{\varGamma }{\mathcal {L}} _{{\text{ imp }}}^{\nabla }{{\textbf {g}}}_{T}=\mathop {{\text{ div }}}\nolimits _{\varGamma }\,{{\textbf {g}}}_{T}$ so that

$$\begin{aligned} \left\| \mathop {{\text {div}}}\nolimits _{\varGamma }\,{\textbf{H}}_{\varGamma } {\textbf{g}}_{T}\right\| _{H^{s^{\prime }-1}\left( \varGamma \right) }&\le {\tilde{C}}_{s^{\prime },s}\left( \lambda \left| k\right| \right) ^{s^{\prime }-s}\left\| \varDelta _{\varGamma }{\mathcal {L}}_{{\text {imp}} }^{\nabla }{\textbf{g}}_{T}\right\| _{H^{s-1}\left( \varGamma \right) } \\&={\tilde{C}}_{s^{\prime },s}\left( \lambda \left| k\right| \right) ^{s^{\prime }-s}\left\| \mathop {{\text {div}}}\nolimits _{\varGamma }\,{\textbf{g}} _{T}\right\| _{H^{s-1}\left( \varGamma \right) }. \end{aligned}$$

This shows (6.22b). The proof of (6.22c) follows along the same lines by using $\mathop {{\text {curl}}}\nolimits _{\varGamma }\,\overrightarrow{\mathop {{\text {curl}}}\nolimits _{\varGamma }}=-\varDelta _{\varGamma }$ and $\mathop {{\text {curl}}}\nolimits _{\varGamma }\,\nabla _{\varGamma }=0$. $\square$

The following lemma concerns the parameter-explicit bounds for the low frequency operators.

#### Lemma 6.5

Let $\lambda >1$ be fixed in the Definition (6.2) of $L_{{\mathbb {R}}^{3}}$. There exists a constant $C>0$ independent of $\lambda$ such that for all $p\in {\mathbb {N}}_{0}$, ${\textbf{v}}\in {\textbf{L}}^{2}({\mathbb {R}}^{3})$, ${\textbf{w}}\in {\textbf{L}} ^{2}(\varOmega )$, there holds

6.23a $$\begin{aligned} \bigl \vert L_{{\mathbb {R}}^{3}}{\textbf{v}}\bigr \vert _{{\textbf{H}}^{p} ({\mathbb {R}}^{3})}&\le C\left( \lambda |k|\right) ^{p}\left\| {\textbf{v}}\right\| _{{\textbf{L}}^{2}({\mathbb {R}}^{3})}, \end{aligned}$$

6.23b $$\begin{aligned} \bigl \vert L_{\varOmega }{\textbf{w}}\bigr \vert _{{\textbf{H}}^{p}(\varOmega )}+\bigl \vert L_{\varOmega }^{0}{\textbf{w}}\bigr \vert _{{\textbf{H}}^{p}(\varOmega )}&\le C\left( \lambda |k|\right) ^{p}\left\| {\textbf{w}}\right\| . \end{aligned}$$

For the boundary frequency filter we have, due to the analyticity of $\varGamma$, the existence of $C>0$ and a neighborhood ${\mathcal {U}}_{\varGamma }\subset {\mathbb {R}}^{3}$ of $\varGamma$ (depending only on $\varOmega$) and some $\gamma >0$ (depending additionally on $\lambda$) such that for each ${\textbf{z}}_{T} \in {\textbf{H}}_{T}^{-1/2}(\varGamma )$ there exists a function

$$\begin{aligned} {\textbf{Z}}\in {\mathcal {A}}\bigl ( C\left\| {\textbf{z}}_{T}\right\| _{{\textbf{H}}^{-1/2}(\varGamma )},\gamma ,{\mathcal {U}}_{\varGamma }\bigr ) \end{aligned}$$

such that $${\textbf{Z}}|_{\varGamma }=L_{\varGamma }{\textbf{z}}_{T}$$.

#### Proof

From [37, (3.32b)] for the full space and from [38, Lem. 4.3] for bounded domains the estimates (6.23a) and the first one in (6.23b) follow. For the operator $L_{\varOmega }^{0}$, recall the lifting operator ${\textbf{R}}_{2}$ of (2.6). Then, for any ${\textbf{w}}\in {\textbf{L}}^{2}( \varOmega )$ and $p\in {\mathbb {N}}_{0}$, the second estimate in (6.23b) follows from

$$\begin{aligned} \bigl \vert L_{\varOmega }^{0}{\textbf{w}}\bigr \vert _{{\textbf{H}}^{p}(\varOmega )}&\le \bigl \vert L_{\varOmega }^{0}{\textbf{w}}\bigr \vert _{{\textbf{H}}^{p} ({\mathbb {R}}^{3})}=\left| {\text {curl}}\,L_{{\mathbb {R}}^{3}} {\mathcal {E}}_{{\text {Stein}}}{\textbf{R}}_{2}{\textbf{w}}\right| _{{\textbf{H}}^{p}({\mathbb {R}}^{3})}\\&=\left| L_{{\mathbb {R}}^{3}} {\text {curl}}\,{\mathcal {E}}_{{\text {Stein}}}{\textbf{R}}_{2} {\textbf{w}}\right| _{{\textbf{H}}^{p}({\mathbb {R}}^{3})}\\&\overset{(\text {6.23a})}{\le }C(\lambda |k|)^{p}\left\| {\text {curl}}\,{\mathcal {E}}_{{\text {Stein}}}{\textbf{R}}_{2} {\textbf{w}}\right\| \le C(\lambda |k|)^{p}\left\| {\textbf{w}}\right\| . \end{aligned}$$

Finally, we consider the boundary low frequency operator. For ${\textbf{z}} _{T}\in {\textbf{H}}^{-1/2}(\varGamma )$, we define functions in the volume $\varOmega$ via

$$\begin{aligned} \varPhi :=L_{\varOmega }{\mathcal {E}}_{\varOmega }^{\varDelta }{\mathcal {L}}_{{\text {imp}} }^{\nabla }{\textbf{z}}_{T},\qquad \varPsi :=L_{\varOmega }{\mathcal {E}}_{\varOmega }^{\varDelta }{\mathcal {L}}_{{\text {imp}}}^{{\text {curl}}}{\textbf{z}}_{T} \end{aligned}$$

so that, for ${\varvec{\phi }}:=\nabla \varPhi$ and ${\varvec{\psi }}:=\nabla \varPsi$,

$$\begin{aligned} {\textbf{L}}_{\varGamma }{\textbf{z}}_{T}&=\varPi _{T}\nabla \varPhi +\varPi _{T}\nabla \varPsi \times {\textbf{n}} ={\textbf{n}}\times \left( \left. {\varvec{\phi }} \right| _{\varGamma }\times {\textbf{n}}\right) +\left. {\varvec{\psi }} \right| _{\varGamma }\times {\textbf{n}}. \end{aligned}$$

Let ${\textbf{n}}^{*}$ denote an analytic extension of the normal vector field into the domain $\varOmega$; due to the analyticity of the domain we may assume that there are constants $C_{{\textbf{n}}}$, $\gamma _{{\textbf{n}}}>0$ and a tubular neighborhood ${\mathcal {U}}_{ \varGamma }\subset \varOmega$ with $\varGamma \subset \overline{{\mathcal {U}}_{ \varGamma }}$ such that ${\textbf{n}}^{*}\in {\mathcal {A}}^{\infty }\left( C_{{\textbf{n}} },\gamma _{{\textbf{n}}},{\mathcal {U}}_{ \varGamma }\right)$. Let

$$\begin{aligned} {\textbf{N}}_{*}:=\left[ \begin{array}{ccc} 0 &{} n_{3}^{*} &{} -n_{2}^{*}\\ -n_{3}^{*} &{} 0 &{} n_{1}^{*}\\ n_{2}^{*} &{} -n_{1}^{*} &{} 0 \end{array} \right] . \end{aligned}$$

Then,

$$\begin{aligned} \left. {\varvec{\psi }} \right| _{\varGamma }\times \mathbf {n=}\left( {\textbf{N}}_{*} {\varvec{\psi }} \right) _{\varGamma }\quad \text {and}\quad {\textbf{n}}\times \left( \left. {\varvec{\phi }} \right| _{\varGamma }\times {\textbf{n}}\right) =-\left( {\textbf{N}}_{*}^{2} {\varvec{\phi }} \right) _{\varGamma }, \end{aligned}$$

i.e.,

6.24 $$\begin{aligned} {\textbf{L}}_{\varGamma }{\textbf{z}}_{T}=\left. {\textbf{G}}_{{\textbf{z}}}\right| _{\varGamma }\quad \text {for }{\textbf{G}}_{{\textbf{z}}}:={\textbf{N}}_{*}\left( {\varvec{\psi }} -{\textbf{N}}_{*} {\varvec{\phi }} \right) . \end{aligned}$$

We further have for $p\in {\mathbb {N}}_{0}$

$$\begin{aligned} \left| {\varvec{\phi }} \right| _{{\textbf{H}}^{p}({\mathcal {U}}_{ \varGamma })}&=\bigl \vert \nabla L_{\varOmega }{\mathcal {E}}_{\varOmega }^{\varDelta } {\mathcal {L}}_{{\text {imp}}}^{\nabla }{\textbf{z}}_{T}\bigr \vert _{{\textbf{H}}^{p}({\mathcal {U}}_{ \varGamma })} =\bigl \vert L_{{\mathbb {R}}^{3}}\nabla {\mathcal {E}}_{{\text {Stein}} }{\mathcal {E}}_{\varOmega }^{\varDelta }{\mathcal {L}}_{{\text {imp}}}^{\nabla }{\textbf{z}}_{T}\bigr \vert _{{\textbf{H}}^{p}({\mathcal {U}}_{ \varGamma })} \\&\le C(\lambda |k|)^{p}\bigl \Vert \nabla {\mathcal {E}}_{{\text {Stein}} }{\mathcal {E}}_{\varOmega }^{\varDelta }{\mathcal {L}}_{{\text {imp}}}^{\nabla }{\textbf{z}}_{T}\bigr \Vert _{{\textbf{L}}^{2}({\mathbb {R}}^{3})}\le C(\lambda |k|)^{p}\bigl \Vert {\mathcal {E}}_{\varOmega }^{\varDelta }{\mathcal {L}} _{{\text {imp}}}^{\nabla }{\textbf{z}}_{T}\bigr \Vert _{{\textbf{H}} ^{1}(\varOmega )}\\&\le C\left( \lambda |k|\right) ^{p}\bigl \Vert {\mathcal {L}} _{{\text {imp}}}^{\nabla }{\textbf{z}}_{T}\bigr \Vert _{{\textbf{H}} ^{1/2}(\varGamma )}\le C_{1}\left( \lambda |k|\right) ^{p}\bigl \Vert {\textbf{z}}_{T}\bigr \Vert _{{\textbf{H}}^{-1/2}( \varGamma ) }. \end{aligned}$$

The proof of the estimate

$$\begin{aligned} \left| {\varvec{\psi }} \right| _{{\textbf{H}}^{p}\left( {\mathcal {U}}_{ \varGamma }\right) }\le C_{2}\left( \lambda \left| k\right| \right) ^{p}\left\| {\textbf{z}}_{T}\right\| _{{\textbf{H}}^{-1/2}\left( \varGamma \right) } \end{aligned}$$

follows along the same lines. Next we use [33, Lem. 4.3.1] (an inspection of the proof shows that this lemma also holds for $d=3$) to deduce that ${\textbf{N}}_{*}{\varvec{\psi }}$, $-{\textbf{N}}_{*}^{2} {\varvec{\phi }}\in {\mathcal {A}}\bigl ( C\left\| {\textbf{z}}_{T}\right\| _{{\textbf{H}}^{-1/2}( \varGamma ) },\gamma ,{\mathcal {U}}_{ \varGamma }\bigr )$, where *C* depends only on $C_{1}$, $C_{2}$, $C_{{\textbf{n}}}$, $\gamma _{{\textbf{n}}}$, while $\gamma$ depends additionally on $\lambda$. $\square$

## *k*-Explicit Regularity by Decomposition

In this section, we always assume that the bounded Lipschitz domain $\varOmega \subset {\mathbb {R}}^{3}$ has a simply connected, analytic boundary $\varGamma =\partial \varOmega$. We consider the Maxwell problem (2.40) with data ${\textbf{f}}$, ${\textbf{g}}_{T}$ with finite regularity.

For the regularity analysis of the operator ${\mathcal {S}}_{\varOmega ,k}^{{\text {MW}}}$ it is key to understand that the solutions for high frequency right-hand sides have low order regularity but well-behaved stability constant (with respect to the wavenumber) while solutions corresponding to low-frequency right-hand sides are analytic but with possibly growing stability constant. This different behavior is reflected in the regularity theory, which decomposes the solution ${\textbf{z}}={\mathcal {S}} _{\varOmega ,k}^{{\text {MW}}}({\textbf{f}},{\textbf{g}}_{T})$ into a part with finite regularity that can be controlled uniformly in *k* and an analytic part that can be controlled explicitly in *k*. This is achieved in Theorem 7.3. The main idea of the proof is to exploit that the operators ${\mathcal {L}}_{\varOmega ,k}$ and ${\mathcal {L}}_{\varOmega ,{\text {i}}\, k}$ have the same leading order differential operator. With the filter operators of the preceding Sect. 6 and recalling $I=H_{\varOmega }^{0}+L_{\varOmega }^{0}+{\textbf{S}}=H_{\varOmega }^{0}+L_{\varOmega } ^{0}+H_{\varOmega }{\textbf{S}}+L_{\varOmega }{\textbf{S}}$ as well as $I={\textbf{H}} _{\varGamma }+{\textbf{L}}_{\varGamma }$ one can write

$$\begin{aligned} {\mathcal {S}}_{\varOmega ,k}^{{\text {MW}}}({\textbf{f}},{\textbf{g}} _{T})={\mathcal {S}}_{\varOmega ,k}^{+}(H_{\varOmega }^{0}{\textbf{f}}+H_{\varOmega } {\textbf{S}}{\textbf{f}},{\textbf{H}}_{\varGamma }{\textbf{g}}_{T})+{\mathcal {S}}_{\varOmega ,k}^{{\text {MW}}}(L_{\varOmega }^{0}{\textbf{f}}+L_{\varOmega }{\textbf{S}} {\textbf{f}},{\textbf{L}}_{\varGamma }{\textbf{g}}_{T})+{\textbf{z}}^{\prime } \end{aligned}$$

for a remainder ${\textbf{z}}^{\prime }$. One then makes the following observations:

1. If ${\text {div}}\,{\textbf{f}} = 0$ (which may be achieved by subtracting a suitable gradient field), then the operator ${\textbf{S}}$ is smoothing by (6.6).
2. The term ${\mathcal {S}}_{\varOmega ,k}^{+}(H_{\varOmega }^{0} {\textbf{f}} + H_{\varOmega }{\textbf{S}}{\textbf{f}},{\textbf{H}}_{\varGamma }{\textbf{g}}_{T})$ has finite regularity properties given by Theorem 4.3. The effect of the high frequency filters $H_{\varOmega }^{0}$ and ${\textbf{H}}_{\varGamma }$ is that they improve the *k*-dependence of lower-order terms in the indexed norms such as $\Vert \cdot \Vert _{{\textbf{H}}^{m}(\varOmega ),k}$ (see Lemma 7.1 below).
3. ${\mathcal {S}}_{\varOmega ,k}^{{\text {MW}}}(L_{\varOmega }^{0} {\textbf{f}} + L_{\varOmega }{\textbf{S}} {\textbf{f}},{\textbf{L}}_{\varGamma }{\textbf{g}}_{T})$ is an analytic function and can be estimated with the aid of Theorem 5.2.
4. The function ${\textbf{z}}^{\prime }$ satisfies
  $$\begin{aligned} {\mathcal {L}}_{\varOmega ,k}^{{\text {MW}}}{\textbf{z}}^{\prime }={\textbf{r}},\qquad {\mathcal {B}}_{ \varGamma ,k}=0, \end{aligned}$$
  where, by suitably choosing the cut-off parameters $\lambda$ in the frequency operators, the residual ${\textbf{r}}$ satisfies $\Vert {\textbf{r}}\Vert _{*}\le q\Vert {\textbf{f}}\Vert _{*}$ for some $q\in (0,1)$ and a suitable norm $\Vert \cdot \Vert _{*}$. Hence, the arguments can be repeated for ${\textbf{z}}^{\prime }$ and the decomposition can be obtained by a geometric series argument.

### The Concatenation of ${\mathcal {S}}_{\varOmega ,k}^{+}$ with High Frequency Filters

The following lemma analyzes the mapping properties of the concatenation of the solution operator ${\mathcal {S}}_{\varOmega ,k}^{+}$ with the high frequency filter operators $H_{\varOmega }^{0}$ and ${\textbf{H}}_{\varGamma }$.

#### Lemma 7.1

Let $$m\in {\mathbb {N}}_{0}$$, $$\ell \in {\mathbb {N}}_{0}$$. Provided the right-hand sides are finite, the following estimates hold with a constant *C* independent of $$\lambda , f ,$$ and $$g_T $$:

7.1 $$\begin{aligned} | k| ^{m}\Vert H_{\varOmega }^{0}{\textbf{f}}\Vert _{{\textbf{H}}^{m}( {\text {div}},\varOmega ) ,k}&\le C\left| k\right| \left\| {\textbf{f}}\right\| _{{\textbf{H}} ^{m}(\varOmega )}, \end{aligned}$$

7.2 $$\begin{aligned} |k|^{m-1}\Vert H_{\varOmega }^{0}{\textbf{f}}\Vert _{{\textbf{H}}^{m -1}({\text {div}},\varOmega ),k}&\le C\lambda ^{-1}\Vert {\textbf{f}} \Vert _{{\textbf{H}}^{m}(\varOmega )},\qquad m \ge 1, \end{aligned}$$

7.3 $$\begin{aligned} {|k|}\Vert H_{\varOmega }^{0}{\textbf{f}}\Vert _{{\textbf{X}}_{{\text {imp}} }^{\prime }( \varOmega ) ,k}&\le C\lambda ^{-1/2}\Vert {\textbf{f}}\Vert _{{\textbf{L}}^{2}(\varOmega )} , \end{aligned}$$

7.4 $$\begin{aligned} |k|^{m+2}\Vert {\mathcal {S}}_{\varOmega ,k}^{+}({H}_{\varOmega }^{0}{\textbf{f}} ,0)\Vert _{{\textbf{H}}^{m}(\varOmega ),k}&\le C\lambda ^{-1/2}\Vert {\textbf{f}}\Vert _{{\textbf{H}}^{m}(\varOmega )}, \end{aligned}$$

as well as

7.5 $$\begin{aligned}&|k|^{m-1}\Vert {\textbf{H}}_{\varGamma }{\textbf{g}}_{T}\Vert _{{\textbf{H}} ^{m-1/2}({\text {div}}_{\varGamma },\varGamma ),k} \nonumber \\&\quad \le C(\lambda |k|)^{-\ell }\left( |k|\Vert {\textbf{g}}_{T}\Vert _{{\textbf{H}}^{m-1/2+\ell }(\varGamma )} +\Vert {\text {div}}_{\varGamma }{\textbf{g}}_{T}\Vert _{{H}^{m-1/2+\ell } (\varGamma )}\right) , \end{aligned}$$

7.6 $$\begin{aligned}&|k|^{m+2}\Vert {\mathcal {S}}_{\varOmega ,k}^{+}(0,{\textbf{H}}_{\varGamma }{\textbf{g}} _{T})\Vert _{{\textbf{H}}^{m}(\varOmega ),k} \nonumber \\&\quad \le C(\lambda |k|)^{-\ell } {\left\{ \begin{array}{ll} \lambda ^{-1}\left( |k|\Vert {\textbf{g}}_{T}\Vert _{{\textbf{H}}^{m-1/2+\ell }(\varGamma )}+\Vert {\text {div}}_{\varGamma }{\textbf{g}}_{T}\Vert _{{\textbf{H}} ^{m-1/2+\ell }(\varGamma )}\right) , &{} m\ge 1,\\ \left( |k|\Vert {\textbf{g}}_{T}\Vert _{{\textbf{H}}^{m-1/2+\ell }(\varGamma )} +\Vert {\text {div}}_{\varGamma }{\textbf{g}}_{T}\Vert _{{\textbf{H}}^{m-1/2+\ell }(\varGamma )}\right) , &{} m=0. \end{array}\right. } \end{aligned}$$

For ${\textbf{f}}\in \mathbf {L^{2}}(\varOmega )$ with ${\text {div}}\,{\textbf{f}}=0$ and the operator ${\textbf{S}}$ of (6.5) we have for any $n\in {\mathbb {N}}_{0}$

7.7 $$\begin{aligned} |k|^{n}\Vert H_{\varOmega }{\textbf{S}}{\textbf{f}}\Vert _{{\textbf{H}}^{m}(\varOmega ),k}&\le C_{n}\lambda ^{-n}\Vert {\textbf{f}}\Vert _{{\textbf{L}}^{2}(\varOmega )}, \end{aligned}$$

7.8 $$\begin{aligned} |k|^{n}\Vert {\mathcal {S}}_{\varOmega ,k}^{+}(H_{\varOmega }{\textbf{S}}{\textbf{f}} ,0)\Vert _{{\textbf{H}}^{m+1}(\varOmega ),k}&\le C_{n}\lambda ^{-n}\Vert {\textbf{f}}\Vert _{{\textbf{L}}^{2}(\varOmega )}. \end{aligned}$$

#### Proof

*Proof of* (7.1): (7.1) follows from the fact that ${\text {div}} H_{\varOmega }^{0} {\textbf{f}} = 0$ and Proposition 6.4.

*Proof of* (7.2): For $m \ge 1$, we estimate

$$\begin{aligned}&\Vert {H}_{\varOmega }^{0}{\textbf{f}}\Vert _{{\textbf{H}}^{m-1}(\varOmega ),k} \le C\left( \Vert {H}_{\varOmega }^{0}{\textbf{f}}\Vert _{{\textbf{L}}^{2}(\varOmega )}+|k|^{-(m-1)}|{H}_{\varOmega }^{0}{\textbf{f}}|_{{\textbf{H}}^{m-1}(\varOmega )}\right) \\&\quad \overset{\text {Prop. }\text {6.4}}{\le }C\left( (\lambda |k|)^{-m}+|k|^{-(m-1)}(\lambda |k|)^{-1}\right) \Vert {\textbf{f}}\Vert _{{\textbf{H}}^{m}(\varOmega )}\le C\lambda ^{-1}|k|^{-m }\Vert {\textbf{f}}\Vert _{{\textbf{H}}^{m}(\varOmega )}. \end{aligned}$$

Noting that ${\text {div}}H_{\varOmega }^{0}{\textbf{f}}=0$, the estimate (7.2) follows.

*Proof of* (7.3): Recall the definition of $\Vert \cdot \Vert _{{\textbf{X}}_{{\text {imp}}}^{\prime }(\varOmega ),k}$ in (4.3) and observe with the multiplicative trace inequality,

7.9 $$\begin{aligned} \Vert \gamma _{T}H_{{\mathbb {R}}^{3}}{\mathcal {E}}_{{\text {Stein}}} {\textbf{R}}_{2}{\textbf{f}}\Vert _{{\textbf{L}}^{2}(\varGamma )}\le C(\lambda |k|)^{-1/2}\Vert {\mathcal {E}}_{{\text {Stein}}}{\textbf{R}}_{2} {\textbf{f}}\Vert _{{\textbf{H}}^{1}({\mathbb {R}}^{3})}\le C(\lambda |k|)^{-1/2} \Vert {\textbf{f}}\Vert _{{\textbf{L}}^{2}(\varOmega )}. \nonumber \\ \end{aligned}$$

Since $H_{\varOmega }^{0}{\textbf{f}}={\text {curl}}H_{{\mathbb {R}}^{3} }{\mathcal {E}}_{{\text {Stein}}}{\textbf{R}}_{2}{\textbf{f}}$, we get with an integration by parts ([41, Thm. 3.29]) for ${{\textbf{v}}} \in {{\textbf{X}}}_{{\text {imp}}}$

$$\begin{aligned} \left| (H_{\varOmega }^{0}{{\textbf{f}}},{{\textbf{v}}})\right|&=\left| (\gamma _{T}H_{{{\mathbb {R}}}^{3}}{{\mathcal {E}}}_{{\text {Stein}} }{{\textbf{R}}}_{2}{{\textbf{f}}},{{\textbf{v}}}_{T})_{{\textbf{L}}^{2}(\varGamma )}+(H_{{\mathbb {R}}^{3}}{{\mathcal {E}}}_{{\text {Stein}}}{{\textbf{R}}} _{2}{{\textbf{f}}},{\text {curl}}\,{{\textbf{v}}})\right| \\&\le C(\lambda |k|)^{-1/2}\Vert {{\textbf{f}}}\Vert _{{\textbf{L}}^{2}(\varOmega )}\Vert {{\textbf{v}}}_{T}\Vert _{{\textbf{L}}^{2}(\varGamma )}+(\lambda |k|)^{-1} \Vert {{\textbf{f}}}\Vert _{{\textbf{L}}^{2}(\varOmega )}\Vert {\text {curl}}\,{{\textbf{v}}}\Vert _{{\textbf{L}}^{2}(\varOmega )}\\&\le C\lambda ^{-1/2}|k|^{-1}\Vert {{\textbf{f}}}\Vert _{{{\textbf{L}}}^{2}(\varOmega )} \Vert {{\textbf{v}}}\Vert _{{\text {imp}},k}. \end{aligned}$$

We conclude $\displaystyle \left| k\right| ^{1}\Vert H_{\varOmega }^{0} {\textbf{f}}\Vert _{{\textbf{X}}_{{\text {imp}}}^{\prime }( \varOmega ),k}\le C\lambda ^{-1/2}\Vert {\textbf{f}}\Vert _{{\textbf{L}} ^{2}(\varOmega )}$ by the definition (4.3) of $\Vert \cdot \Vert _{{\textbf{X}} _{{\text {imp}}}^{\prime }( \varOmega ),k}$, and the statement (7.3) is shown.

*Proof of* (7.4): For $m\ge 1$, we obtain (7.4) from (7.2) and Theorem 4.3, (4.18a). For $m=0$, we observe that Theorem 4.3, (4.17) and (7.3) imply $\Vert {\mathcal {S}}_{\varOmega ,k} ^{+}(H_{\varOmega }^{0}{\textbf{f}},0)\Vert _{{\textbf{L}}^{2}(\varOmega )}\le |k|^{-1}\Vert {\mathcal {S}}_{\varOmega ,k}^{+}(H_{\varOmega }^{0}{\textbf{f}},0)\Vert _{{\text {imp}},k}\le C{|k|^{-1}}\Vert H_{\varOmega }^{0} {\textbf{f}}\Vert _{{\textbf{X}}_{{\text {imp}}}^{\prime }( \varOmega ),k} \le C\lambda ^{-1/2}|k|^{-2}\Vert {\textbf{f}}\Vert _{{\textbf{L}}^{2}(\varOmega )}$.

*Proof of* (7.5): We distinguish the cases $m=0$ and $m\ge 1$. For $m\ge 1$, the statement follows from the estimates of ${{\textbf{H}}}_{\varGamma }$ given in Proposition 6.4. For $m=0$, in addition to Proposition 6.4 one invokes Lemma 4.2.

*Proof of* (7.6): For $m\ge 1$, the estimate (7.6) follows from combining Proposition 6.4 with (4.18a) and (7.5) (taking $\ell +1$ for $\ell$ there) to get

$$\begin{aligned}&\Vert {\mathcal {S}}_{\varOmega ,k}^{+}(0,{\textbf{H}}_{\varGamma }{\textbf{g}}_{T} )\Vert _{{\textbf{H}}^{m}(\varOmega ),k} \le C|k|^{-3}\Vert {\textbf{H}}_{\varGamma }{\textbf{g}}_{T}\Vert _{{\textbf{H}}^{m-1-1/2}({\text {div}}_{\varGamma } ,\varGamma ),k}\\&\quad \overset{(\text {7.5})}{\le }C|k|^{-3-m+2}(\lambda |k|)^{-1-\ell }\left( |k|\Vert {\textbf{g}}_{T}\Vert _{{\textbf{H}}^{m-1/2+\ell }(\varGamma )}+\Vert {\text {div}}_{\varGamma }{\textbf{g}}_{T}\Vert _{H^{m-1/2+\ell }(\varGamma )}\right) . \end{aligned}$$

For $m=0$, we use Theorem 4.3, (4.17) to get

$$\begin{aligned}&\Vert {\mathcal {S}}_{\varOmega ,k}^{+}(0,{\textbf{H}}_{\varGamma }{\textbf{g}}_{T} )\Vert _{{\textbf{L}}^{2}(\varOmega )} \le C|k|^{-1}\Vert {\mathcal {S}}_{\varOmega ,k}^{+}(0,{\textbf{H}}_{\varGamma }{\textbf{g}}_{T})\Vert _{{\text {imp}},k}\le C|k|^{-1}\Vert {\textbf{H}}_{\varGamma }{\textbf{g}}_{T}\Vert _{{\textbf{X}} _{{\text {imp}}}^{\prime }\left( \varGamma \right) ,k}\\&\qquad \overset{(\text {4.12})}{\le }C\left| k\right| ^{-2}\left( |k|\Vert {\textbf{H}}_{\varGamma }{\textbf{g}}_{T}\Vert _{{\textbf{H}} ^{-1/2}(\varGamma )}+\Vert {\text {div}}_{\varGamma }\,{\textbf{H}}_{\varGamma } {\textbf{g}}_{T}\Vert _{{H}^{-1/2}(\varGamma )}\right) \\&\qquad \overset{\text {Prop. 6.4}}{\le }C\left| k\right| ^{-2}(\lambda |k|)^{-\ell }\left( |k|\Vert {\textbf{g}}_{T} \Vert _{{\textbf{H}}^{-1/2+\ell }(\varGamma )}+\Vert {\text {div}}_{\varGamma } {\textbf{g}}_{T} \Vert _{{H}^{-1/2+\ell }(\varGamma )}\right) . \end{aligned}$$

This completes the proof of (7.6).

*Proof of* (7.7), (7.8): For ${\textbf{f}}$ with ${\text {div}}\,{\textbf{f}}=0$ we have ${\textbf{S}}{\textbf{f}}\in C^{\infty }( \varOmega )$ by (6.6). Hence, (7.7), (7.8) follow from Proposition 6.4 and (6.6) and again Theorem 4.3. $\square$

#### Lemma 7.2

Let $m\in {\mathbb {N}}_{0}$, ${\text {div}}\,{\textbf{f}}\in H^{m-1}(\varOmega )$, ${\text {div}}_{\varGamma }{\textbf{g}}_{T}\in H^{m-3/2}(\varGamma )$. Then there exist $\varphi _{{\textbf{f}}}\in H^{m+1} (\varOmega )\cap H_{0}^{1}(\varOmega )$ and $\varphi _{{\textbf{g}}}\in H^{m+1}(\varOmega )$ such that for $\ell =0,\ldots ,m$

$$\begin{aligned} \Vert \varphi _{{\textbf{f}}}\Vert _{H^{\ell +1}(\varOmega )}&\le C\Vert {\text {div}}\,{\textbf{f}}\Vert _{{\textbf{H}}^{\ell -1}(\varOmega )},&- {\text {div}}\,\nabla \varphi _{{\textbf{f}}}&={\text {div}}\,{\textbf{f}},&{\mathcal {B}}_{ \varGamma ,k}\nabla \varphi _{{\textbf{f}}}&=0,\\ \Vert \varphi _{{\textbf{g}}}\Vert _{H^{\ell +1}(\varOmega )}&\le C\Vert {\text {div}}_{\varGamma }\,{\textbf{g}}_{T}\Vert _{{\textbf{H}}^{\ell -3/2}(\varGamma )},&{\text {div}}\,\nabla \varphi _{{\textbf{g}}}&=0,&{\text {div}} _{\varGamma }\,\nabla _{\varGamma }\varphi _{{\textbf{g}}}&={\text {div}}_{\varGamma }\,{\textbf{g}}_{ T} . \end{aligned}$$

#### Proof

Define $\varphi _{{\textbf{f}}}\in H_{0}^{1}(\varOmega )$ as the weak solution of

$$\begin{aligned} - \varDelta \varphi _{{\textbf{f}}}={\text {div}}\,{\textbf{f}}. \end{aligned}$$

By elliptic regularity, we have $\varphi _{{\textbf{f}}}\in H^{m+1}(\varOmega )$ and the stated bounds. The function $\varphi _{{\textbf{g}}}$ is defined in two steps: First, let $\varphi \in H^{1}(\varGamma )$ with $\int _{\varGamma }\varphi =0$ denote the weak solution of

$$\begin{aligned} \varDelta _{\varGamma }\varphi ={\text {div}}_{\varGamma }\,{\textbf{g}}_{T}, \end{aligned}$$

which satisfies $\Vert \varphi \Vert _{H^{m+1/2}(\varGamma )}\le C\Vert {\text {div}}\,{\textbf{g}}_{T}\Vert _{H^{m-3/2}(\varGamma )}$. Then, $\varphi _{{\textbf{g}}}$ is defined on $\varOmega$ as the harmonic extension from $\varGamma$, i.e., $\varphi _{{\textbf{g}}}\in H^{m+1}(\varOmega )$ solves

$$\begin{aligned} \varDelta \varphi _{{\textbf{g}}}=0,\qquad \varphi _{{\textbf{g}}}|_{\varGamma }=\varphi . \end{aligned}$$

Again, the bounds follow from elliptic regularity theory. $\square$

### Regularity by Decomposition: The Main Result

#### Theorem 7.3

Let $\varOmega \subset {\mathbb {R}}^{3}$ be a bounded Lipschitz domain with a simply connected, analytic boundary $\varGamma =\partial \varOmega$. Let the stability Assumption (3.2) be satisfied. Then there is a linear mapping ${\textbf{L}}^{2}(\varOmega )\times {\textbf{H}}_{T}^{-1/2} ({\text {div}}_{\varGamma },\varGamma )\ni ({\textbf{f}},{\textbf{g}}_{T} )\mapsto ({\textbf{z}}_{H^{2}},{\textbf{z}}_{{\mathcal {A}}},\varphi _{{\textbf{f}} },\varphi _{{\textbf{g}}})$ such that the solution ${\textbf{z}}:={\mathcal {S}} _{\varOmega ,k}^{{\text {MW}}}({\textbf{f}},{\textbf{g}}_{T})\in {\textbf{X}} _{{\text {imp}}}$ of (2.40) can be written as ${\textbf{z}} ={\textbf{z}}_{H^{2}}+{\textbf{z}}_{{\mathcal {A}}}+k^{-2}\nabla \varphi _{{\textbf{f}} }+{\text {i}}\,k^{-1}\nabla \varphi _{{\textbf{g}}}$.

The linear mapping has the following properties: For any *m*, $m^{\prime } \in {\mathbb {N}}_{0}$, there are constants *C*, $B>0$ (depending only on $\varOmega$ and *m*, $m^{\prime }$) such that for $({\textbf{f}},{\textbf{g}}_{T})\in {\textbf{H}}^{m}(\varOmega )\times {\textbf{H}}_{T}^{m-1/2}(\varGamma )$ with $({\text {div}}\,{\textbf{f}},{\text {div}}_{\varGamma }{\textbf{g}}_{T})\in H^{m^{\prime }-1}(\varOmega )\times H^{m^{\prime }-3/2}(\varGamma )$ the following holds:

(i) The function ${\textbf{z}}_{H^{2}}$ satisfies
  7.10 $$\begin{aligned} \Vert {\textbf{z}}_{H^{2}}\Vert _{{\textbf{H}}^{m+1}(\varOmega ),k}\le C|k|^{-m-2} \left( |k|\Vert {\textbf{g}}_{T}\Vert _{{\textbf{H}}^{m-1/2}(\varGamma )} +\Vert {\textbf{f}}\Vert _{{\textbf{H}}^{m}(\varOmega )}\right) . \end{aligned}$$
  If $${\textbf{g}}_{T}\in {\textbf{H}}_{T}^{m+1/2}(\varGamma )$$, then
  7.11 $$\begin{aligned} \left\| {\textbf{z}}_{H^{2}}\right\| _{{\textbf{H}}^{m+1}\left( {\text {curl}},\varOmega \right) ,k}\le C\left| k\right| ^{-m-1}\left( \left\| {\textbf{g}}_{T}\right\| _{{\textbf{H}} ^{m+1/2}\left( \varGamma \right) }+\left\| {\textbf{f}}\right\| _{{\textbf{H}}^{m}\left( \varOmega \right) }\right) \end{aligned}$$
  and in (7.10) the term $|k|\Vert {\textbf{g}}_{T}\Vert _{{\textbf{H}}^{m-1/2}(\varGamma )}$ can be replaced with $\Vert {\textbf{g}}_{T} \Vert _{{\textbf{H}}^{m+1/2}(\varGamma )}$.
(ii) The gradient fields $\nabla \varphi _{{\textbf{f}}}$ and $\nabla \varphi _{{\textbf{g}}}$ are given by Lemma 7.2 and satisfy, for $\ell =0,\ldots ,m^{\prime }$:
  7.12 $$\begin{aligned} \left\| \varphi _{{\textbf{f}}}\right\| _{{\textbf{H}}^{\ell +1}(\varOmega )}&\le C\left\| {\text {div}}\,{\textbf{f}}\right\| _{H^{\ell -1}\left( \varOmega \right) }, \end{aligned}$$
  7.13 $$\begin{aligned} \left\| \varphi _{{\textbf{g}}}\right\| _{{\textbf{H}}^{\ell +1}(\varOmega )}&\le C\left\| {\text {div}}_{\varGamma }\,{\textbf{g}}_{T}\right\| _{H^{\ell -3/2}\left( \varGamma \right) }. \end{aligned}$$
(iii) The analytic part ${\textbf{z}}_{{\mathcal {A}}}$ satisfies
  7.14 $$\begin{aligned} {\textbf{z}}_{{\mathcal {A}}}\in {\mathcal {A}}(C{(1+C_\mathrm{{stab}})}|k|^{ \theta -1} \{\Vert {\textbf{f}}\Vert +|k|\Vert {\textbf{g}}_{T}\Vert _{{\textbf{H}}^{-1/2}(\varGamma )}\},B,\varOmega ). \end{aligned}$$

#### Proof

By linearity of the solution operator ${\mathcal {S}}_{\varOmega ,k} ^{{\text {MW}}}$, we consider the cases ${\mathcal {S}}_{\varOmega ,k}^{{\text {MW}}}({\textbf{f}},0)$ and ${\mathcal {S}}_{\varOmega ,k} ^{{\text {MW}}}(0,{\textbf{g}}_{T})$ separately. The fact that the right-hand sides in (7.10), (7.11), (7.14) do not contain the divergence of ${\textbf{f}}$ or ${\textbf{g}}_{T}$ is due to the fact that we suitably choose the functions $\varphi _{{\textbf{f}}}$, $\varphi _{{\textbf{g}}}$ in the course of the proof.

*Step 1* (reduction to divergence-free data): Let the functions $\varphi _{{\textbf{f}}}$, $\varphi _{{\textbf{g}}}$ be given by Lemma 7.2. These functions have the regularity properties given in (ii). The function ${\textbf{z}} ^{\prime }:={\textbf{z}}-k^{-2}\nabla \varphi _{{\textbf{f}}}-{\text {i}}\, k^{-1}\nabla \varphi _{{\textbf{g}}}$ satisfies

$$\begin{aligned} {\mathcal {L}}_{\varOmega ,k}{\textbf{z}}^{\prime }&={\textbf{f}} + \nabla \varphi _{{\textbf{f}}} + {\text {i}}\,k\nabla \varphi _{{\textbf{g}}}=:{\textbf{f}}^{\prime } \quad \text{ in } \varOmega ,\\ {\mathcal {B}}_{ \varGamma ,k}{\textbf{z}}^{\prime }&={\textbf{g}}_{T}-\nabla _{\varGamma }\varphi _{{\textbf{g}} }=:{\textbf{g}}_{T}^{\prime }\quad \text{ on } \varGamma . \end{aligned}$$

By construction, ${\text {div}}\,{\textbf{f}}^{\prime }=0$ and ${\text {div}}_{\varGamma }{\textbf{g}}_{T}^{\prime }=0$. Furthermore, using $\Vert {\text {div}}\,{\textbf{f}}\Vert _{{\textbf{H}}^{m-1}(\varOmega )}\le C\Vert {\textbf{f}}\Vert _{{\textbf{H}}^{m}(\varOmega )}$ and $\Vert {\text {div}} _{\varGamma }{\textbf{g}}_{T}\Vert _{{\textbf{H}}^{m-3/2}(\varGamma )}\le C\Vert {\textbf{g}}_{T}\Vert _{{\textbf{H}}^{m-1/2}(\varGamma )}$ we obtain

7.15 $$\begin{aligned} \Vert {\textbf{f}}^{\prime }\Vert _{{\textbf{H}}^{m}(\varOmega )}&\le C\left( \Vert {\textbf{f}}\Vert _{{\textbf{H}}^{m}(\varOmega )}+C|k|\Vert {\textbf{g}}_{T} \Vert _{{\textbf{H}}^{m-1/2}(\varGamma )}\right) , \end{aligned}$$

7.16 $$\begin{aligned} \Vert {\textbf{g}}_{T}^{\prime }\Vert _{{\textbf{H}}^{m-1/2}(\varGamma )}&\le C\Vert {\textbf{g}}_{T}\Vert _{{\textbf{H}}^{m-1/2}(\varGamma )}. \end{aligned}$$

*Step 2* (Analysis of ${\mathcal {S}}_{\varOmega ,k}^{{\text {MW}} }({\textbf{f}}^{\prime },0)$ with ${\text {div}}\,{\textbf{f}}^{\prime }=0$): We claim that

7.17 $$\begin{aligned} {\mathcal {S}}_{\varOmega ,k}^{{\text {MW}}}({\textbf{f}}^{\prime },0)={{\textbf{z}} }_{H^{2},{{\textbf{f}}}}+{{\textbf{z}}}_{{{\mathcal {A}}},{{\textbf{f}}}} \end{aligned}$$

for some functions ${{\textbf{z}}}_{H^{2},{{\textbf{f}}}}$ and ${{\textbf{z}} }_{{{\mathcal {A}}},{{\textbf{f}}}}$ satisfying the estimates (7.11) (and therefore also (7.10) since we focus on the case ${{\textbf{g}}}_T= 0)$ and (7.14). We have ${\text {div}}\,{\textbf{f}}^{\prime }=0$ and assume ${\textbf{g}}_{T}=0$, which implies ${\textbf{g}}_{T}^{\prime }=0$. Set ${\textbf{f}}_{0}^{\prime }:=\widetilde{{\textbf{f}}}_{0}:={\textbf{f}}^{\prime }$ and define, with the mapping ${\textbf{f}}\mapsto \varphi _{{\textbf{f}}}$ of Lemma 7.2, recursively for $n=0,1,\ldots ,$

7.18 $$\begin{aligned} {\textbf{z}}_{H^{2},n}&:={\mathcal {S}}_{\varOmega ,k}^{+}(H_{\varOmega }^{0} \widetilde{{\textbf{f}}}_{n},0)+{\mathcal {S}}_{\varOmega ,k}^{+}(H_{\varOmega } {\textbf{S}}\widetilde{{\textbf{f}}}_{n},0), \nonumber \\ {\textbf{z}}_{{\mathcal {A}},n}&:={\mathcal {S}}_{\varOmega ,k}^{{\text {MW}} }(L_{\varOmega }^{0}\widetilde{{\textbf{f}}}_{n},0)+{\mathcal {S}}_{\varOmega ,k}^{{\text {MW}}}(L_{\varOmega }{\textbf{S}}\widetilde{{\textbf{f}}} _{n},0),\nonumber \\ {\textbf{f}}_{n+1}^{\prime }&:=2k^{2}{\textbf{z}}_{H^{2},n},\nonumber \\ \widetilde{{\textbf{f}}}_{n+1}&:={\textbf{f}}_{n+1}^{\prime }+ \nabla \varphi _{{\textbf{f}}_{n+1}^{\prime }}. \end{aligned}$$

We note that ${\text {div}}\widetilde{{\textbf{f}}}_{n}=0$ for all *n*. From Lemma 7.1, we get: if $\widetilde{{\textbf{f}}}_{n}\in {\textbf{H}}^{\ell }( \varOmega )$, then

7.19 $$\begin{aligned} |k|^{\ell +2}\Vert {\mathcal {S}}_{\varOmega ,k}^{+}({H}_{\varOmega }^{0}\widetilde{{\textbf{f}}}_{n},0)\Vert _{{\textbf{H}}^{\ell }(\varOmega ),k}\le C\lambda ^{-1/2} \Vert \widetilde{{\textbf{f}}}_{n}\Vert _{{\textbf{H}}^{\ell }(\varOmega )}. \end{aligned}$$

Next, we obtain from Lemma 7.2 and the above defined recurrence relation

7.20 $$\begin{aligned} \Vert \nabla \varphi _{{\textbf{f}}_{n}^{\prime }}\Vert _{{\textbf{H}}^{\ell }(\varOmega )}&\le C\Vert {\text {div}}\,{\textbf{f}}_{n}^{\prime }\Vert _{{\textbf{H}} ^{\ell -1}(\varOmega )}\le C\Vert {\textbf{f}}_{n}^{\prime }\Vert _{{\textbf{H}}^{\ell }(\varOmega )},\qquad \ell =0,\ldots ,m, \end{aligned}$$

7.21 $$\begin{aligned} \Vert {\textbf{f}}_{n+1}^{\prime }\Vert _{{\textbf{H}}^{\ell }(\varOmega )}&\overset{(\text {7.19})}{\le }C\lambda ^{-1/2}\Vert \widetilde{{\textbf{f}}}_{n}\Vert _{{\textbf{H}}^{\ell }(\varOmega )}\le C\lambda ^{-1/2} \Vert {\textbf{f}}_{n}^{\prime }\Vert _{{\textbf{H}}^{\ell }(\varOmega )}, \ \ell =0,\ldots ,m, \end{aligned}$$

7.22 $$\begin{aligned} \Vert \widetilde{{\textbf{f}}}_{n+1}\Vert _{{\textbf{H}}^{m}(\varOmega )}&\le C\Vert {\textbf{f}}_{n+1}^{\prime }\Vert _{{\textbf{H}}^{m}(\varOmega )}\le C\lambda ^{-1/2}\Vert \widetilde{{\textbf{f}}}_{n}\Vert _{{\textbf{H}}^{m}(\varOmega )}. \end{aligned}$$

From the equation that defines ${\textbf{z}}_{H^{2},n}$ and since ${\text {div}}\,H_{\varOmega }^{0}\widetilde{{\textbf{f}}}_{n}=0$ we get

7.23 $$\begin{aligned} ( 2k^{2}) ^{-1}{\text {div}}\,{\textbf{f}}_{n+1}^{\prime }={\text {div}}\,{\textbf{z}}_{H^{2},n}=k^{-2}{\text {div}}\,H_{\varOmega }{\textbf{S}}\widetilde{{\textbf{f}}}_{n}. \end{aligned}$$

Since ${\textbf{S}}$ is a smoothing operator, this implies that ${\text {div}}\,{\textbf{f}}_{n+1}^{\prime }$ is smooth, and the first estimate in (7.20) actually holds for any $\ell \in {\mathbb {N}}_{0}$. The bounds (7.21), (7.22) show that the functions ${\textbf{f}}_{n}$ and $\widetilde{{\textbf{f}}}_{n}$ decay in geometric progression as *n* increases if $\lambda >1$ is chosen such that $C\lambda ^{-1/2}=:q<1$. Fixing such a $\lambda >1$, a geometric series argument implies for any $\mu \in \left\{ 0,1,\ldots ,m\right\}$

7.24 $$\begin{aligned} \sum _{n=0}^{\infty }\Vert \widetilde{{\textbf{f}}}_{n}\Vert _{{\textbf{H}}^{\mu }(\varOmega )}\le C\Vert {\textbf{f}}^{\prime }\Vert _{{\textbf{H}}^{\mu }(\varOmega )}. \end{aligned}$$

We also get from Theorem 4.3 and Lemma 7.1 and the smoothing property of ${{\textbf{S}}}$ (recall that ${\text {div}}{\widetilde{{{\textbf {f}}_{n}}}} = 0$)

7.25 $$\begin{aligned} \Vert {\textbf{z}}_{H^{2},n}\Vert _{{\textbf{H}}^{m+1}({\text {curl}} ,\varOmega ),k}&\!\! \overset{\text {Theorem }\text {4.3}}{\le }\!\!C\left| k\right| ^{-2}\left( \Vert H_{\varOmega }^{0}\widetilde{{\textbf{f}}}_{n}\Vert _{{\textbf{H}}^{m}({\text {div}},\varOmega ),k}+\Vert H_{\varOmega }{\textbf{S}}\widetilde{{\textbf{f}}}_{n}\Vert _{{\textbf{H}}^{m} ({\text {div}},\varOmega ),k}\right) \nonumber \\&\overset{(\text {7.1})}{\le }C|k|^{-(m+1)}\Vert \widetilde{{\textbf{f}}}_{n}\Vert _{{\textbf{H}}^{m}(\varOmega )}. \end{aligned}$$

Lemma 6.5 shows that $L_{\varOmega }^{0}\widetilde{{\textbf{f}}} _{n}$, $L_{\varOmega }{\textbf{S}}\widetilde{{\textbf{f}}}_{n}\in {\mathcal {A}} (C_{1}\Vert \widetilde{{\textbf{f}}}_{n}\Vert _{{\textbf{L}}^{2}(\varOmega )},C_{2}\lambda |k|,\varOmega )$ for some $C_{1}$, $C_{2}$ depending only on $\varOmega$. From Theorem 5.2, we infer

7.26 $$\begin{aligned} |{\textbf{z}}_{{\mathcal {A}},n}|_{{\textbf{H}}^{p}(\varOmega )}\le C_{{\textbf{z}}}{(1+C_\mathrm{{stab}})}|k|^{ \theta -1 } \Vert \widetilde{{\textbf{f}}}_{n}\Vert _{{\textbf{L}}^{2}(\varOmega )}\gamma ^{p} \max \,(p,|k|)^{p}\qquad \forall p\in {\mathbb {N}}_{0}\nonumber \\ \end{aligned}$$

for some $C_{{\textbf{z}}}$, $\gamma$ independent of *k* and *n*; $\gamma$ depends on $\lambda$, which has been fixed above. Upon setting

$$\begin{aligned} {\textbf{z}}_{H^{2},{{\textbf{f}}^{\prime }}}:=\sum _{n=0}^{\infty }{\textbf{z}} _{H^{2},n},\qquad \nabla \varphi :=\sum _{n=1}^{\infty }\nabla \varphi _{{\textbf{f}}_{n}^{\prime }},\qquad {\textbf{z}}_{{\mathcal {A}},{\textbf{f}}}:=\sum _{n=0}^{\infty }{\textbf{z}}_{{\mathcal {A}},n}, \end{aligned}$$

we have by (7.25) and (7.24)

$$\begin{aligned} \left\| {\textbf{z}}_{H^{2},{{\textbf{f}}^{\prime }}}\right\| _{{\textbf{H}} ^{m+1}({\text {curl}},\varOmega ),k}\le & {} C|k|^{-(m+1)}\Vert {\textbf{f}}^{\prime }\Vert _{{\textbf{H}}^{m}(\varOmega )}\le C|k|^{-(m+1)} \Vert {\textbf{f}}\Vert _{{\textbf{H}}^{m}(\varOmega )},\\ {\textbf{z}}_{{\mathcal {A}},{\textbf{f}}}{} & {} \in {\mathcal {A}}(C{(1+C_\mathrm{{stab}})}|k|^{ \theta -1 } \Vert {\textbf{f}}\Vert _{{\textbf{L}}^{2}(\varOmega )},\gamma ,\varOmega ). \end{aligned}$$

For the term $\nabla \varphi$, we get

$$\begin{aligned} \left\| k^{-2}\nabla \varphi \right\| _{{\textbf{H}}^{m+1} ({\text {curl}},\varOmega ),k}= & {} \left| k\right| ^{-1}\left\| \nabla \varphi \right\| _{{\textbf{H}}^{m+1}(\varOmega ),k}\le C\left| k\right| ^{-1}\sum _{n=1}^{\infty }\left\| {\text {div}}\,{\textbf{f}}_{n}^{\prime }\right\| _{{\textbf{H}}^{m}\left( \varOmega \right) ,k}\\{} & {} \overset{(\text {7.23})}{=}C\left| k\right| ^{-1}\sum _{n=0}^{\infty }\Vert 2{\text {div}}\,H_{\varOmega }{\textbf{S}} \widetilde{{\textbf{f}}}_{n}\Vert _{{\textbf{H}}^{m}\left( \varOmega \right) ,k}\\{} & {} \le C\sum _{n=0}^{\infty }\Vert H_{\varOmega }{\textbf{S}}\widetilde{{\textbf{f}} }_{n}\Vert _{{\textbf{H}}^{m+1}\left( \varOmega \right) ,k}\le C\left| k\right| ^{-\left( m+1\right) }\sum _{n=0}^{\infty }\Vert \widetilde{{\textbf{f}}}_{n}\Vert _{{\textbf{L}}^{2}\left( \varOmega \right) }, \end{aligned}$$

where we used (7.7) with $n\leftarrow m+1$ for the last estimate. The combination with (7.24) shows

$$\begin{aligned} \Vert k^{-2}\nabla \varphi \Vert _{{\textbf{H}}^{m+1} ({\text {curl}},\varOmega ),k}\le C\left| k\right| ^{-\left( m+1\right) }\Vert {\textbf{f}}^{\prime }\Vert _{{\textbf{L}}^{2}( \varOmega ) }. \end{aligned}$$

We set

$$\begin{aligned} {{\textbf{z}}}_{H^{2},{{\textbf{f}}}}:={{\textbf{z}}}_{H^{2},{{\textbf{f}}}^{\prime } }+ k^{-2}\nabla \varphi . \end{aligned}$$

That is, the terms ${\textbf{z}}_{H^{2},{\textbf{f}}}$ and ${\textbf{z}} _{{\mathcal {A}},{\textbf{f}}}$ satisfy the estimates (7.10), (7.11) given in the statement of the theorem for the present case ${{\textbf{g}}}_T = 0$. We compute

$$\begin{aligned} {\mathcal {L}}_{\varOmega ,k}({\textbf{z}}_{H^{2},{\textbf{f}}}+{\textbf{z}}_{{\mathcal {A}},{\textbf{f}}})=\sum _{n=0}^{\infty }{\textbf{f}}_{n}^{\prime }-{\textbf{f}} _{n+1}^{\prime }={\textbf{f}}_{0}^{\prime }={\textbf{f}}^{\prime },\qquad {\mathcal {B}}_{ \varGamma ,k}({\textbf{z}}_{H^{2},{{\textbf{f}}}}+{\textbf{z}}_{{\mathcal {A}},{\textbf{f}}})=0. \end{aligned}$$

By the uniqueness assertion of Proposition 3.1, we have identified ${\textbf{z}}_{H^{2},{\textbf{f}}}+{\textbf{z}}_{{\mathcal {A}},{\textbf{f}}}={\mathcal {S}}_{\varOmega ,k}^{{\text {MW}}}({\textbf{f}}^{\prime },0)$.

*Step 3* (Analysis of ${\mathcal {S}}_{\varOmega ,k}^{{\text {MW}} }(0,{\textbf{g}}_{T}^{\prime })$ with ${\text {div}}_{\varGamma }{\textbf{g}} _{T}^{\prime }=0$): We define

$$\begin{aligned} {\textbf{z}}_{H^{2},{\textbf{g}}}:={\mathcal {S}}_{\varOmega ,k}^{+}(0,{\textbf{H}} _{\varGamma }{\textbf{g}}_{T}^{\prime }),\qquad {\textbf{z}}_{{\mathcal {A}},{\textbf{g}} }:={\mathcal {S}}_{\varOmega ,k}^{{\text {MW}}}(0,{\textbf{L}}_{\varGamma } {\textbf{g}}_{T}^{\prime }). \end{aligned}$$

From Theorem 4.3 and the properties of ${\textbf{H}}_{\varGamma }$ given in Proposition 6.4 we get

7.27 $$\begin{aligned} \Vert {\textbf{z}}_{H^{2},{\textbf{g}}}\Vert _{{\textbf{H}}^{m+1}(\varOmega ),k}&\le C|k|^{-3}\Vert {\textbf{H}}_{\varGamma }{\textbf{g}}_{T}^{\prime }\Vert _{{\textbf{H}} ^{m-1/2}({\text {div}}_{\varGamma },\varGamma ),k} \nonumber \\&\overset{(\text {7.5}),{\text {div}}_{\varGamma }\,{\textbf{g}} _{T}^{\prime }=0}{\le }C|k|^{-1-m}\Vert {\textbf{g}}_{T}\Vert _{{\textbf{H}} ^{m-1/2}(\varGamma )}, \end{aligned}$$

7.28 $$\begin{aligned} \Vert {\textbf{z}}_{H^{2},{\textbf{g}}}\Vert _{{\textbf{H}}^{m+1}({\text {curl}} ,\varOmega ),k}&\overset{\text {Prop. }\text {6.4} }{\le }C|k|^{-(m+1)}\Vert {\textbf{g}}_{T}\Vert _{{\textbf{H}}^{m+1/2}(\varGamma )}. \end{aligned}$$

That is, ${\textbf{z}}_{H^{2},{\textbf{g}}}$ satisfies the estimates (7.10), (7.11) given in the statement of the theorem for the present case ${{\textbf{f}}} = 0$. For ${\textbf{z}}_{{\mathcal {A}},{\textbf{g}}}$ we observe that Lemma 6.5 ensures^7^${\textbf{L}}_{\varGamma }{\textbf{g}}_{T}^{\prime }\in {\mathcal {A}}(C\Vert {\textbf{g}} _{T}^{\prime }\Vert _{{\textbf{H}}^{-1/2}(\varGamma )},\gamma ,\varOmega )$ for some *C* depending only on $\varOmega$ and $\gamma >0$ depending on $\varOmega$ and $\lambda$. We note $\Vert {\textbf{g}}_{T}^{\prime }\Vert _{{\textbf{H}}^{-1/2}(\varGamma )}\le C\Vert {\textbf{g}}_{T}\Vert _{{\textbf{H}}^{-1/2}(\varGamma )}$ by (7.16). From Theorem 5.2 we obtain

$$\begin{aligned} {\textbf{z}}_{{\mathcal {A}},{\textbf{g}}}\in {\mathcal {A}}(C{(1+C_\mathrm{{stab}})}|k|^{ \theta -1/2} \Vert {\textbf{g}}_{T}\Vert _{{\textbf{H}}^{-1/2}(\varGamma )},\gamma ,\varOmega ). \end{aligned}$$

Since $\theta -1/2\le \theta$, the function ${\textbf{z}}_{{\textbf{A}},{\textbf{g}}}$ satisfies the estimate stated in the theorem. Finally, we observe that $\widehat{{\textbf{z}} }:={\mathcal {S}}_{\varOmega ,k}^{{\text {MW}}}(0,{\textbf{g}}_{T}^{\prime })-({\textbf{z}}_{H^{2},{\textbf{g}}}+{\textbf{z}}_{{\mathcal {A}},{\textbf{g}}})$ satisfies

$$\begin{aligned} {\mathcal {L}}_{\varOmega ,k}\widehat{{\textbf{z}}}=2k^{2}{\mathcal {S}}_{\varOmega ,k} ^{+}(0,{\textbf{H}}_{\varGamma }{\textbf{g}}_{T}^{\prime })=:\widehat{{\textbf{f}} },\qquad {\mathcal {B}}_{ \varGamma ,k}\widehat{{\textbf{z}}}=0. \end{aligned}$$

From Lemma 7.1 we get using ${\text {div}}_{\varGamma }{\textbf{g}}_{T}^{\prime }=0$

7.29a $$\begin{aligned} \Vert \widehat{{\textbf{f}}}\Vert _{{\textbf{H}}^{m}(\varOmega )}&\le C|k|\Vert {\textbf{g}}_{T}^{\prime }\Vert _{{\textbf{H}}^{m-1/2}(\varGamma )},&\Vert \widehat{{\textbf{f}}}\Vert _{{\textbf{H}}^{m}(\varOmega )}&\le C\Vert {\textbf{g}} _{T}^{\prime }\Vert _{{\textbf{H}}^{m+1/2}(\varGamma )}, \end{aligned}$$

7.29b $$\begin{aligned} \Vert \widehat{{\textbf{f}} }\Vert _{{\textbf{L}}^{2}(\varOmega )}&\le C|k|\Vert {\textbf{g}}_{T}^{\prime } \Vert _{{\textbf{H}}^{-1/2}(\varGamma )}. \end{aligned}$$

We note that ${\text {div}}\widehat{{\textbf{f}}}=0$ and that Step 2 provides a decomposition of $\widehat{{\textbf{z}}}$ in the form $\widehat{{\textbf{z}}}={\textbf{z}}_{H^{2},\widehat{{\textbf{f}}}}+{\textbf{z}}_{{\mathcal {A}},\widehat{{\textbf{f}}}}$. By Step 2, the term ${\textbf{z}}_{H^{2},\widehat{{\textbf{f}}}}$ can be controlled in terms of $\Vert \widehat{{\textbf{f}}}\Vert _{{\textbf{H}}^{m}(\varOmega )}$ and thus in the required form. For ${\textbf{z}}_{{\mathcal {A}},\widehat{{\textbf{f}}}}$, we note that Step 2 yields

$$\begin{aligned} {\textbf{z}}_{{\mathcal {A}},\mathbf {\widehat{{\textbf{f}}}}}\!\in \!{\mathcal {A}}(C{(1\!+\!C_\mathrm{{stab}})}|k|^{ \theta -1 } \!\Vert \widehat{{\textbf{f}}}\Vert _{{\textbf{L}}^{2}(\varOmega )},\!\gamma ,\!\varOmega \! )\!\subset \!{\mathcal {A}}(C{(1\!+\!C_\mathrm{{stab}})}|k|^{ \theta } \!\Vert {\textbf{g}}_{T}\Vert _{{\textbf{H}}^{-1/2}(\varGamma )},\gamma ,\varOmega ), \end{aligned}$$

which is an analytic function with the desired estimate. We summarize that ${\textbf{z}}_{H^{2}}$, ${\textbf{z}}_{{\mathcal {A}}}$ in the statement of the theorem are given by

$$\begin{aligned} {\textbf{z}}_{H^{2}}={\textbf{z}}_{H^{2},{\textbf{f}}} +{\textbf{z}}_{H^{2},{\textbf{g}}}+{\textbf{z}}_{H^{2},\widehat{{\textbf{f}}}} \quad \text {and}\quad {\textbf{z}}_{{\mathcal {A}}}={\textbf{z}}_{{\mathcal {A}},{\textbf{f}}}+{\textbf{z}}_{{\mathcal {A}},{\textbf{g}}}+{\textbf{z}}_{{\mathcal {A}},\widehat{{\textbf{f}}}} \end{aligned}$$

and the summands have been estimated in Step 1-3.

*Step 4*: The proof is now complete with the exception of the statement in (i) that $|k|\Vert {\textbf{g}}_{T}\Vert _{{\textbf{H}} ^{m-1/2}(\varGamma )}$ can be replaced with $\Vert {\textbf{g}}_{T}\Vert _{{\textbf{H}}^{m+1/2}(\varGamma )}$. However, this follows directly from (7.11) via

$$\begin{aligned} \left| k\right| \left\| {\textbf{z}}_{H^{2},{\textbf{g}}}\right\| _{{\textbf{H}}^{m+1}\left( \varOmega \right) ,k}\le \left\| {\textbf{z}} _{H^{2},{\textbf{g}}}\right\| _{{\textbf{H}}^{m+1}\left( {\text {curl}},\varOmega \right) ,k}\overset{(\text {7.28})}{\le }C\left| k\right| ^{-m-1}\left\| {\textbf{g}}_{T}\right\| _{{\textbf{H}} ^{m+1/2}\left( \varGamma \right) } \end{aligned}$$

and for the control of $\widehat{{\textbf{z}}}$ in Step 3 via the bound $\Vert \widehat{{\textbf{f}}}\Vert _{{{\textbf{H}}^{m}(\varOmega )}}\lesssim \Vert {{\textbf{g}}}_{T}^{\prime }\Vert _{{{\textbf{H}}^{m+1/2}(\varGamma )}}$ in (7.29). $\square$

## Discretization

In this section, we describe the *hp*-FEM based on Nédélec elements and discuss the approximation properties of various *hp*-approximation operators. These operators made their appearance already in [39]. Here, we strengthen the results of [39, Sec. 8] in that we additionally control the error on the boundary of the elements, which is required due to the impedance boundary conditions considered here.

### Meshes and Nédélec Elements

The classical example of curl-conforming FE spaces are the Nédélec elements, [42]. We restrict our attention here to so-called “type I” elements (sometimes also referred to as the Nédélec-Raviart-Thomas element) on tetrahedra. These spaces are based on a conforming (no hanging nodes), shape-regular triangulation ${{\mathcal {T}}}_{h}$ of $\varOmega \subset {\mathbb {R}}^{3}$. That is, ${{\mathcal {T}}}_{h}$ satisfies:

(i) The (open) elements $K\in {{\mathcal {T}}}_{h}$ cover $\varOmega$, i.e., $\overline{\varOmega }=\cup _{K\in {{\mathcal {T}}}_{h}}{\overline{K}}$.
(ii) Associated with each element *K* is the *element map*, a $$C^{1} $$-diffeomorphism $$F_{K}:\overline{{\widehat{K}}} \rightarrow {\overline{K}}$$. The set $${\widehat{K}}$$ is the *reference tetrahedron*.
(iii) Denoting $h_{K}={\text {diam}}K$, there holds, with some *shape-regularity constant* $\gamma _{{\mathcal {T}}}$,
  8.1 $$\begin{aligned} h_{K}^{-1}\Vert F_{K}^{\prime }\Vert _{L^{\infty }({\widehat{K}})}+h_{K}\Vert (F_{K}^{\prime })^{-1}\Vert _{L^{\infty }({\widehat{K}})}\le \gamma _{{\mathcal {T}}}. \end{aligned}$$
(iv) The intersection of two elements is only empty, a vertex, an edge, a face, or they coincide (here, vertices, edges, and faces are the images of the corresponding entities on the reference tetrahedron ${\widehat{K}}$). The parametrization of common edges or faces are compatible. That is, if two elements *K*, $K^{\prime }$ share an edge (i.e., $F_{K}(e)=F_{K^{\prime } }(e^{\prime })$ for edges *e*, $e^{\prime }$ of ${\widehat{K}}$) or a face (i.e., $F_{K}(f)=F_{K^{\prime }}(f^{\prime })$ for faces *f*, $f^{\prime }$ of ${\widehat{K}}$), then $F_{K}^{-1}\circ F_{K^{\prime }}:f^{\prime }\rightarrow f$ is an affine isomorphism.

The maximal mesh width is denoted by

8.2 $$\begin{aligned} h:=\max \left\{ h_{K}:K\in {\mathcal {T}}_{h}\right\} . \end{aligned}$$

The following assumption requires that the element map $F_{K}$ can be decomposed as a composition of an affine scaling with an *h*-independent mapping. We adopt the setting of [37, Sec. 5] and assume that the element maps $F_{K}$ of the conforming, $\gamma$-shape regular triangulation ${{\mathcal {T}}}_{h}$ satisfy the following additional requirements:

#### Assumption 8.1

(*normalizable regular triangulation*) Each element map $F_{K}$ can be written as $F_{K}=R_{K}\circ A_{K}$, where $A_{K}$ is an *affine* map and the maps $R_{K}$ and $A_{K}$ satisfy for constants $C_{{\text {affine}}}$, $C_{{\text {metric}}}$, $\gamma >0$ independent of *K*:

$$\begin{aligned}&\Vert A_{K}^{\prime }\Vert _{L^{\infty }({\widehat{K}})}\le C_{{\text {affine}}}h_{K},\qquad \Vert (A_{K}^{\prime })^{-1} \Vert _{L^{\infty }({\widehat{K}})}\le C_{{\text {affine}}}h_{K}^{-1},\\&\Vert (R_{K}^{\prime })^{-1}\Vert _{L^{\infty }({\widetilde{K}})}\le C_{{\text {metric}}},\qquad \Vert \nabla ^{n}R_{K}\Vert _{L^{\infty }({\widetilde{K}})}\le C_{{\text {metric}}}\gamma ^{n}n!\qquad \forall n\in {{\mathbb {N}}}_{0}. \end{aligned}$$

Here, ${\widetilde{K}}=A_{K}({\widehat{K}})$ and $h_{K}>0$ is the element diameter.

#### Remark 8.2

A prime example of meshes that satisfy Assumption 8.1 are those patchwise structured meshes as described, for example, in [37, Ex. 5.1] or [33, Sec. 3.3.2]. These meshes are obtained by first fixing a macro triangulation of $\varOmega$; the actual triangulation is then obtained as images of affine triangulations of the reference element. $\square$

On the reference tetrahedron ${\widehat{K}}$ we introduce the classical Nédélec type I and Raviart-Thomas elements of degree $p\ge 0$ (see, e.g., [41]):

8.3 $$\begin{aligned} {{\mathcal {P}}}_{p}({\widehat{K}})&:={\text {span}}\{{\textbf{x}}^{\alpha }\,|\,|\alpha |\le p\}, \end{aligned}$$

8.4 $$\begin{aligned} \textbf{RT}_{p}({\widehat{K}})&:=\{{\textbf{p}}({\textbf{x}})+{\textbf{x}} {q}({\textbf{x}})\,|\,{\textbf{p}}\in ({{\mathcal {P}}}_{p}({\widehat{K}}))^{3},{q} \in {{\mathcal {P}}}_{p}({\widehat{K}})\}, \end{aligned}$$

8.5 $$\begin{aligned} \varvec{{\mathcal {N}}}_{p}^{{\text {I}}}({\widehat{K}})&:=\{{\textbf{p}}({\textbf{x}})+{\textbf{x}}\times {\textbf{q}}({\textbf{x}} )\,|\,{\textbf{p}},{\textbf{q}}\in ({{\mathcal {P}}}_{p}({\widehat{K}}))^{3}\}. \end{aligned}$$

The spaces $S_{p+1}({{\mathcal {T}}}_{h})$, $\textbf{RT}_{p}\left( {\mathcal {T}}_{h}\right)$, $\varvec{{\mathcal {N}}}_{p}^{{\text {I}} }({{\mathcal {T}}}_{h})$ are then defined as in [41, (3.76)] by transforming covariantly $\varvec{{\mathcal {N}}}_{p}^{{\text {I}} }({\widehat{K}})$ and contravariantly $\textbf{RT}_p({{{\widehat{K}}}})$:

8.6a $$\begin{aligned} S_{p+1}({{\mathcal {T}}}_{h})&:=\{u\in H^{1}(\varOmega )\,|\,u|_{K}\circ F_{K} \in {{\mathcal {P}}}_{p+1}({\widehat{K}})\}, \end{aligned}$$

8.6b $$\begin{aligned} \textbf{RT}_{p}({{\mathcal {T}}}_{h})&:=\{{\textbf{u}}\in {\textbf{H}} ({\text {div}},\varOmega )\,|\,({{\text {det}}\,F_{K}^{\prime }} )(F_{K}^{\prime })^{-1}{\textbf{u}}|_{K}\circ F_{K}\in \textbf{RT}_{p}(\widehat{K})\}, \end{aligned}$$

8.6c $$\begin{aligned} \varvec{{\mathcal {N}}}_{p}^{{\text {I}}}({{\mathcal {T}}}_{h})&:=\{{\textbf{u}}\in {\textbf{H}}({\text {curl}},\varOmega )\,|\,(F_{K}^{\prime })^{T}{\textbf{u}}|_{K}\circ F_{K}\in \varvec{{\mathcal {N}}}_{p} ^{{\text {I}}}({\widehat{K}})\}. \end{aligned}$$

We set^8^

8.7 $$\begin{aligned} {\textbf{X}}_{h}&:=\varvec{{\mathcal {N}}}_{p}^{{\text {I}} }({{\mathcal {T}}}_{h})\cap {\textbf{X}}_{{\text {imp}}},&S_{h}&:=S_{p+1}({{\mathcal {T}}}_{h})\cap H_{{\text {imp}}}^{1}\left( \varOmega \right) \end{aligned}$$

and recall the well-known exact sequence property

8.8 $$\begin{aligned} S_{h}\overset{\nabla }{\longrightarrow }{\textbf{X}}_{h}\overset{{\text {curl}}}{\longrightarrow }{\text {curl}}\,{\textbf{X}}_{h}. \end{aligned}$$

The *hp*-FEM Galerkin discretization for the electric Maxwell problem (2.39) is given by:

8.9 $$\begin{aligned} \text {find }{\textbf{E}}_{h}\in {\textbf{X}}_{h}\quad \text { such that }A_{k}( {\textbf{E}}_{h},{\textbf{v}}) =\left( \textbf{j,v}\right) +\left( {\textbf{g}}_{T},{\textbf{v}}\right) _{{\textbf{L}}^{2}\left( \varGamma \right) }\quad \forall {\textbf{v}}\in {\textbf{X}}_{h}. \end{aligned}$$

### *hp*-Approximation Operators

We will use polynomial approximation operators that are constructed elementwise, i.e., for an operator ${\widehat{I}}_{p}$ on the reference element ${\widehat{K}}$, a global operator $I_{p}$ is defined by setting $(I_{p}u)|_{K}:={\widehat{I}}_{p}(u\circ F_{K}))\circ F_{K}^{-1}$. If ${\widehat{I}}_{p}$ maps into ${{\mathcal {P}}}_{p+1}({\widehat{K}})$, then we say ${\widehat{I}}_{p}$ *admits an element-by-element construction*, if the operator $I_{p}$ defined in this way maps into $S_{p+1}({{\mathcal {T}}}_{h})$. Analogously, if ${\widehat{I}}_{p}$ maps into $\varvec{{\mathcal {N}}} _{p}^{{\text {I}}}({\widehat{K}})$, then we say that ${\widehat{I}}_{p}$ *admits an element-by-element construction* if the resulting operator $I_{p}$ maps into $\varvec{{\mathcal {N}}}_{p}^{{\text {I}} }({\mathcal {T}}_{h})$.

For scalar functions (or gradient fields), we have elemental approximation operators with the optimal convergence in $L^{2}$ and $H^{1}$:

#### Lemma 8.3

Let ${\widehat{K}}\subset {\mathbb {R}}^{d}$, $d\in \{2,3\}$, be the reference triangle or reference tetrahedron and ${\mathbb {R}} \ni m \ge (d+1)/2$. Then, for every $p\in {\mathbb {N}}_{0}$, there exists a linear operator ${\widehat{\varPi }}_{p}:H^{ m }({\widehat{K}})\rightarrow {\mathcal {P}}_{p+1}$ that permits an element-by-element construction such that if $p\ge m-2$

8.10 $$\begin{aligned} \begin{aligned}&\Vert u-{\widehat{\varPi }}_{p}u\Vert _{L^{2}({\widehat{K}})} +\frac{1}{p+1}\Vert u-{\widehat{\varPi }}_{p}u\Vert _{H^{1}({\widehat{K}})} + (p+1)^{-1/2}\Vert u-{\widehat{\varPi }}_{p}u\Vert _{L^{2}(\partial {\widehat{K}})} \\&\quad +(p+1)^{-3/2}\Vert u-{\widehat{\varPi }}_{p}u\Vert _{H^{1} (\partial {\widehat{K}})}\le C{(p+1)^{-m}}|u|_{H^{m}({\widehat{K}})} \end{aligned}\nonumber \\ \end{aligned}$$

for a constant $C>0$ that depends only on *m*, *d*, and the choice of reference triangle/tetrahedron.

For the case $d=3$, the condition on *m* can be relaxed to $m >d/2$.

#### Proof

The operator ${\widehat{\varPi }}_{p}$ may be taken as the operators $\widehat{\varPi }^{{\text {grad}},3d}_{p+1}$ for $d = 3$ or $\widehat{\varPi }^{{\text {grad}},2d}_{p+1}$ for $d = 2$ of [36]. The volume estimates follow from [36, Cor. 2.12] for the case $d= 3$ and [36, Thm. 2.13] for the case $d = 2$. For the estimates on $\partial {\widehat{K}}$, one notices that the restriction of ${\widehat{\varPi }}^{{\text {grad}},3d}_{p+1}$ to a boundary face ${\widehat{f}}$ is the operator ${\widehat{\varPi }}^{{\text {grad}},2d}_{p+1}$ on that face and that the restriction of ${\widehat{\varPi }}^{{\text {grad}},2d}_{p+1}$ to an edge of the reference triangle is the operator $\widehat{\varPi }^{{\text {grad}},1d}_{p+1}$ discussed in [36, Lem. 4.1].

For $d=3$ an operator ${\widehat{\varPi }}_{p}$ with the stated approximation properties is constructed in [37, Thm. B.4] for the case $m >d/2=3/2$. The statement about the approximation on $\partial {\widehat{K}}$ follows by a more careful analysis of the proof of [37, Thm. B.4]. For the reader’s convenience, the proof is reproduced in [40, Thm. B.5]. $\square$

The fact that ${\widehat{\varPi }}_{p}$ in Lemma 8.3 has the element-by-element construction property means that an elementwise definition of the operator $\varPi _{p}^{\nabla ,s}:H^{m}(\varOmega )\rightarrow S_{p+1}({\mathcal {T}}_{h})$ by $(\varPi _{p}^{\nabla ,s}\varphi )|_{K}=({\widehat{\varPi }}_{p}(\varphi \circ F_{K}))\circ F_{K}^{-1}$ maps indeed into $S_{p+1}({\mathcal {T}}_{h})\subset H^{1}(\varOmega )$.

In the following we always assume for the spatial dimension $d=3$. By scaling arguments we get the following result:

#### Corollary 8.4

Let $d=3$. For $m\in {\mathbb {N}}_{>3/2}$ and $p\ge m-2$ the operator $\varPi _{p}^{\nabla ,s}:H^{m}(\varOmega )\rightarrow S_{p+1}({\mathcal {T}} _{h})$ has following the approximation properties for all $K\in {\mathcal {T}}_{h}$:

8.11 $$\begin{aligned}&\Vert \varphi -\varPi _{p}^{\nabla ,s}\varphi \Vert _{L^{2}(K)}+\frac{h_{K}}{p+1} \Vert \varphi -\varPi _{p}^{\nabla ,s}\varphi \Vert _{H^{1}(K)} \le C\left( \frac{h_{K}}{p+1}\right) ^{m}\Vert \varphi \Vert _{H^{m}(K)}, \end{aligned}$$

8.12 $$\begin{aligned}&\frac{h_{K}}{p+1}\Vert \varphi -\varPi _{p}^{\nabla ,s}\varphi \Vert _{H^{1}(\partial K)} \le C\left( \frac{h_{K}}{p+1}\right) ^{m-1/2}\Vert \varphi \Vert _{H^{m}(K)}. \end{aligned}$$

In [39, Lem. 8.2] approximation operators ${\widehat{\varPi }}_{p}^{{\text {curl}},s}:{{\textbf{H}}}^{1} ({\text {curl}},{\widehat{K}})\rightarrow \varvec{{\mathcal {N}}} _{p}^{{\text {I}}}({\widehat{K}})$ and ${\widehat{\varPi }}_{p} ^{{\text {div}},s}:{{\textbf{H}}}^{1}({\text {div}},\widehat{K})\rightarrow \textbf{RT}_{p}({\widehat{K}})$ on the reference tetrahedron ${\widehat{K}}$ are defined with certain elementwise approximation properties. Global versions of these operators, $\varPi _{p}^{{\text {curl}},s}:{\textbf{H}}({\text {curl}},\varOmega )\cap {\prod _{K\in {\mathcal {T}}_{h}}}{{\textbf{H}}}^{1} ({\text {curl}}, K )\rightarrow \varvec{{\mathcal {N}}}_{p}^{{\text {I}}}({\mathcal {T}}_{h})$ and $\varPi _{p}^{{\text {div}},s}:{\textbf{H}}\left( {\text {div}},\varOmega \right) \mathbf {\cap }{\prod _{K\in {\mathcal {T}}_{h}}}{{\textbf{H}}}^{1}( K,{\text {div}},{\widehat{K}})\rightarrow \textbf{RT}_{p}\left( {\mathcal {T}}_{h}\right) ,$ are characterized by lifting the operators on the reference element by (cf. [39, Def. 8.1])

8.13 $$\begin{aligned}&(\varPi _{p}^{{\text {curl}},s}{\textbf{u}})|_{K}\circ F_{K} :=(F_{K} ^{\prime })^{-T}{\widehat{\varPi }}_{p}^{{\text {curl}},s}((F_{K}^{\prime })^{\top }{\textbf{u}}\circ F_{K}), \end{aligned}$$

8.14 $$\begin{aligned}&(\varPi _{p}^{{\text {div}},s}{\textbf{u}})|_{K} :=({\text {det}} (F_{K}^{\prime }))^{-1}F_{K}^{\prime }({\widehat{\varPi }}_{p}^{{\text {div}} ,s}({\text {det}}\,F_{K}^{\prime })(F_{K}^{\prime })^{-1}{\textbf{u}}\circ F_{K}))\circ F_{K}^{-1}. \end{aligned}$$

The approximation properties of $\varPi _{p}^{{\text {curl}},s}$ are inferred from those of ${\widehat{\varPi }}_{p}^{{\text {curl}},s}$ given in [39, Lem. 8.2]. We obtain:

#### Lemma 8.5

Let $m\in {\mathbb {N}}_{>3/2}$ and $p\ge m-1$. Let ${\widetilde{C}}$, $B>0$. Then there are constants *C*, $\sigma >0$ depending only on ${\widetilde{C}}$, *B*, *m*, and the constants of Assumption 8.1 such that the following holds for the operator $\varPi _{p}^{{\text {curl}},s}:{\textbf{H}}^{m}(\varOmega )\rightarrow {\varvec{{\mathcal {N}}}}_{p}^{{\text {I}}} ({\mathcal {T}}_{h})$ and all $K\in {\mathcal {T}}_{h}$:

(i) If $${\textbf{u}}\in {\textbf{H}}^{m}(K)$$ then
  8.15 $$\begin{aligned}&\Vert {\textbf{u}}-\varPi _{p}^{{\text {curl}},s}{\textbf{u}}\Vert _{{\textbf{L}} ^{2}(K)}+\frac{h_{K}}{p+1}\Vert {\textbf{u}}-\varPi _{p}^{{\text {curl}} ,s}{\textbf{u}}\Vert _{{\textbf{H}}^{1}(K)} \le C\left( \frac{h_{K}}{p+1}\right) ^{m} \Vert {\textbf{u}}\Vert _{{\textbf{H}}^{m}(K)}, \end{aligned}$$
  8.16 $$\begin{aligned}&\Vert {\textbf{u}}-\varPi _{p}^{{\text {curl}},s}{\textbf{u}}\Vert _{{\textbf{L}} ^{2}(\partial K)} \le C\left( \frac{h_{K}}{p+1}\right) ^{m-1/2} \Vert {\textbf{u}}\Vert _{{\textbf{H}}^{m}(K)}. \end{aligned}$$
(ii) If ${\textbf{u}}\in {\mathcal {A}} (C_{{\textbf{u}}}(K),B,K)$ for some $C_{{\textbf{u}}}(K)>0$ and if
  8.17 $$\begin{aligned} h_{K}+|k|h_{K}/p\le {\widetilde{C}} \end{aligned}$$
  then
  8.18 $$\begin{aligned}&h_{K}^{1/2}\Vert {\textbf{u}}-\varPi _{p}^{{\text {curl}},s}{\textbf{u}} \Vert _{{\textbf{L}}^{2}(\partial {K})}+\Vert {\textbf{u}}-\varPi _{p} ^{{\text {curl}},s}{\textbf{u}}\Vert _{{\textbf{L}}^{2}({K})}+h_{K} \Vert {\textbf{u}}-\varPi _{p}^{{\text {curl}},s}{\textbf{u}}\Vert _{{\textbf{H}} ^{1}({K})}\nonumber \\&\quad \le CC_{{\textbf{u}}}(K)\left( \left( \frac{h_{K}}{h_{K}+\sigma }\right) ^{p+1}+\left( \frac{|k|h_{K}}{\sigma p}\right) ^{p+1}\right) . \end{aligned}$$
(iii) If ${\textbf{u}}\in {\mathcal {A}} (C_{{\textbf{u}}},B,\varOmega )$ for some $C_{{\textbf{u}}}>0$ and if (8.17) holds, then
  $$\begin{aligned} \Vert {\textbf{u}}-\varPi _{p}^{{\text {curl}},s}{\textbf{u}}\Vert _{{\text {imp}},k}\le C_{{\textbf{u}}}|k|\left( \left( \frac{h}{h+\sigma }\right) ^{p}+\left( \frac{|k|h}{\sigma p}\right) ^{p}\right) . \end{aligned}$$

#### Proof

The result follows from modifications of the procedure in [39, Sec. 8.3]. We recall the structure $F_{K}=R_{K}\circ A_{K}$ of the element maps by Assumption 8.1. For $K\in {\mathcal {T}}_{h}$ we define ${\widetilde{K}}:=A_{K}(K)$ and the transformed functions $\widehat{{\textbf{v}}}:=(F_{K}^{\prime })^{\top }{\textbf{v}}\circ F_{K}$ on ${\widehat{K}}$ and $\widetilde{{\textbf{v}}}:=(R_{K}^{\prime })^{\top }{\textbf{v}}\circ R_{K}$ on ${\widetilde{K}}$. We note that $\widehat{{\textbf{v}} }=(A_{K}^{\prime })^{\top }\widetilde{{\textbf{v}}}\circ A_{K}$. By Assumption 8.1 and the fact that $A_{K}$ is affine, we have

8.19 $$\begin{aligned} \Vert \widetilde{{\textbf{v}}}\Vert _{{\textbf{H}}^{j}({\widetilde{K}})}\sim \Vert {{\textbf{v}}}\Vert _{{\textbf{H}}^{j}({K})},\qquad&\Vert \widetilde{{\textbf{v}}}\Vert _{{\textbf{L}}^{2}(\partial {\widetilde{K}})}\sim \Vert {{\textbf{v}} }\Vert _{{\textbf{L}}^{2}(\partial {K})}, \end{aligned}$$

8.20 $$\begin{aligned} |\widehat{{\textbf{v}}}|_{{\textbf{H}}^{j}({\widehat{K}})}\sim h_{K}^{1+j-3/2} |\widetilde{{\textbf{v}}}|_{{\textbf{H}}^{j}({\widetilde{K}})},\quad&\Vert \widehat{{\textbf{v}}}\Vert _{{\textbf{L}}^{2}(\partial {\widehat{K}})}\sim h_{K}^{1-1}\Vert \widetilde{{\textbf{v}}}\Vert _{{\textbf{L}}^{2}(\partial {{\widetilde{K}}})}, \end{aligned}$$

where the implied constant depends only on *j* and the constants of Assumption 8.1.

*Proof of* (i): From [39, Lem. 8.2], we have for

8.21 $$\begin{aligned} p\Vert \widehat{{\textbf{u}}}-{\widehat{\varPi }}_{p}^{{\text {curl}},s} \widehat{{\textbf{u}}}\Vert _{{\textbf{L}}^{2}({\widehat{K}})}+\Vert \widehat{{\textbf{u}}}-{\widehat{\varPi }}_{p}^{{\text {curl}},s}\widehat{{\textbf{u}} }\Vert _{{\textbf{H}}^{1}({\widehat{K}})}\le Cp^{-(m-1)}|{\textbf{u}}|_{{\textbf{H}} ^{m}({\widehat{K}})}. \end{aligned}$$

This approximation result and the scaling argument expressed in (8.19), (8.20) produce (8.15). The multiplicative trace inequality $\Vert \widehat{{\textbf{v}} }\Vert _{{\textbf{L}}^{2}(\partial {\widehat{K}})}^{2}\le C\Vert \widehat{{\textbf{v}}}\Vert _{{\textbf{L}}^{2}({\widehat{K}})}\Vert \widehat{{\textbf{v}}} \Vert _{{\textbf{H}}^{1}({\widehat{K}})}$ applied to (8.21) and similar scaling arguments produce (8.16).

*Proof of* (ii): By [39, Lem. 8.4], the pull-back $\widehat{{\textbf{u}}} \in {\mathcal {A}}(CC_{{\textbf{u}}}(K)h_{K}^{1-3/2},h_{K}B^{\prime },{\widehat{K}})$ for some $B^{\prime }$ depending only on *B* and the constants of Assumption 8.1. By [39, Lem. 8.2] there are constants depending only on *B* and the constants of Assumption 8.1 such that

$$\begin{aligned} \Vert \widehat{{\textbf{u}}}-{\widehat{\varPi }}_{p}^{{\text {curl}},s} \widehat{{\textbf{u}}}\Vert _{W^{2,\infty }({\widehat{K}})}\le Ch_{K} ^{1-3/2}C_{{\textbf{u}}}(K)\left( \left( \frac{h_{K}}{h_{K}+\sigma }\right) ^{p+1}+\left( \frac{|k|h_{K}}{\sigma p}\right) ^{p+1}\right) . \end{aligned}$$

With similar scaling arguments as in the proof of (i), we obtain the stated estimate.

*Proof of* (iii): For each $K\in {\mathcal {T}}_{h}$, we define

$$\begin{aligned} {\widetilde{C}}_{{\textbf{u}}}^{2}(K):=\sum _{n=0}^{\infty }\frac{|{\textbf{u}} |_{{\textbf{H}}^{n}(K)}^{2}}{(2B)^{2n}\max (n+1,|k|)^{2n}} \end{aligned}$$

and note

$$\begin{aligned} {\textbf{u}}\in {\mathcal {A}}({\widetilde{C}}_{{\textbf{u}}}(K),2B,K) \qquad \text{ with } \qquad \sum _{K\in {\mathcal {T}}_{h}}{\widetilde{C}}_{{\textbf{u}}}^{2}(K)\le 2C_{{\textbf{u}}}^{2}. \end{aligned}$$

We then sum the elementwise error estimates provided by (ii). $\square$

### An Interpolating Projector onto the Finite Element Space

For the error analysis, the following subspace of ${\textbf{H}}^{1}( \varOmega )$ will play an important role:

8.22 $$\begin{aligned} {\textbf{V}}_{k,0}:=\left\{ {\textbf{u}}\in {\textbf{X}}_{{\text {imp}}} \mid { \left( \!\left( \hspace{-0.50003pt} {\textbf{u}},\nabla \varphi \hspace{-1.00006pt}\right) \!\right) }_k =0\quad \forall \varphi \in H_{{\text {imp}}}^{1}\left( \varOmega \right) \right\} . \end{aligned}$$

#### Proposition 8.6

Let $\varOmega$ be a bounded Lipschitz domain with simply connected, analytic boundary. The space ${\textbf{V}}_{k,0}$ can alternatively be characterized by

8.23 $$\begin{aligned} {\textbf{V}}_{k,0}=\left\{ {\textbf{u}}\in {\textbf{X}}_{{\text {imp}}} \mid {\text {div}}\,{\textbf{u}}=0\wedge {\text {i}}\,k\left\langle {\textbf{u}},{\textbf{n}}\right\rangle +\mathop {{\text {div}}}\nolimits _{\varGamma }\,{\textbf{u}}_{T}=0\quad \text {on }\varGamma \right\} . \end{aligned}$$

The proof of this proposition is standard and uses the same arguments as, e.g., [39, Lem. 4.10].

#### Proposition 8.7

Let $\varOmega$ be a bounded Lipschitz domain with simply connected analytic boundary. It holds $\displaystyle {\textbf{V}}_{k,0}\subset {\textbf{H}}^{1}(\varOmega )$, and there exists $c>0$ independent of *k* such that

$$\begin{aligned} c\left| k\right| \left\| {\textbf{v}}\right\| _{{\textbf{H}} ^{1}\left( \varOmega \right) ,k}\le \left\| {\textbf{v}}\right\| _{{\textbf{H}}\left( {\text {curl}},\varOmega \right) ,k}\le \left\| {\textbf{v}}\right\| _{{\text {imp}},k}\qquad \forall {\textbf{v}} \in {\textbf{V}}_{k,0}. \end{aligned}$$

#### Proof

The estimate $\Vert {\textbf{v}}\Vert _{{\textbf{H}}({\text {curl}},\varOmega ),k}\le \Vert {\textbf{v}}\Vert _{{\text {imp}},k}$ follows directly from the definition of the norms. For the lower bound, we employ the Helmholtz decomposition of ${\textbf{v}}\in {\textbf{V}}_{k,0}$ as in Lemma 2.7 (i) and take into account ${\text {div}}\,{\textbf{v}}=0$. That is, there exist ${\textbf{w}}\in {\textbf{H}}^{1}(\varOmega )$ and $\varphi \in H^{1}(\varOmega )$ with

8.24 $$\begin{aligned} {\textbf{v}}&=\nabla \varphi +{\textbf{w}} ,&\Vert {\textbf{w}}\Vert _{{\textbf{H}}^{1}(\varOmega )}&\le C\left\| {\text {curl}}\,{\textbf{v}}\right\| . \end{aligned}$$

Since ${\text {div}}\,{\textbf{v}}=0$ we conclude $\Vert {\textbf{v}} \Vert _{{\textbf{H}}({\text {div}},\varOmega )}=\Vert {\textbf{v}}\Vert$ so that a trace theorem gives us

$$\begin{aligned} \Vert \langle {\textbf{v}},{\textbf{n}}\rangle \Vert _{H^{-1/2}( \varGamma ) }\le C\Vert {\textbf{v}}\Vert _{{\textbf{H}}({\text {div}},\varOmega )}=C\Vert {\textbf{v}}\Vert . \end{aligned}$$

It holds

$$\begin{aligned} \varDelta _{\varGamma }( \varphi \vert _{\varGamma }) =\mathop {{\text {div}}}\nolimits _{\varGamma }\,( {\textbf{v}}_{T}-{\textbf{w}} _{T}) \overset{\text {Prop. }\text {8.6}}{=}-{\text {i}}\, k\langle {\textbf{v}},{\textbf{n}}\rangle -\mathop {{\text {div}}}\nolimits _{\varGamma }\,{\textbf{w}}_{T}=:{\tilde{v}}\text {.} \end{aligned}$$

By the smoothness of the closed manifold $\varGamma$ and the shift properties of the Laplace-Beltrami operator we get

$$\begin{aligned} \Vert \varphi \Vert _{H^{3/2}( \varGamma ) }&\le C\Vert {\tilde{v}}\Vert _{H^{-1/2}( \varGamma ) } \le C \left[ \Vert \mathop {{\text {div}}}\nolimits _{\varGamma }\,{\textbf{w}}_{T}\Vert _{H^{-1/2}\left( \varGamma \right) }+\left| k\right| \Vert \langle {\textbf{v}},{\textbf{n}}\rangle \Vert _{H^{-1/2}( \varGamma ) } \right] \\&\le C\left[ \Vert {\textbf{w}}_{T}\Vert _{{\textbf{H}}^{1/2}( \varGamma ) }+|k| \Vert {\textbf{v}}\Vert \right] \le C\left( \left\| {\textbf{w}}\right\| _{{\textbf{H}}^{1}\left( \varOmega \right) }+\left| k\right| \left\| {\textbf{v}}\right\| \right) \\&\overset{(\text {8.24})}{\le }C\left\| {\textbf{v}} \right\| _{{\textbf{H}}\left( {\text {curl}},\varOmega \right) ,k}. \end{aligned}$$

Since $$\varphi $$ solves

$$\begin{aligned} -\varDelta \varphi ={\text {div}}\,{\textbf{w}}\quad \text {in}\, \varOmega , \end{aligned}$$

the shift theorem for the Laplace operator on smooth domains leads to

8.25 $$\begin{aligned} \Vert \nabla \varphi \Vert _{{\textbf{H}}^{1}( \varOmega ) }\le C\!\left( \Vert {\text {div}}\,{\textbf{w}}\Vert +\Vert \varphi \Vert _{H^{3/2}( \varGamma ) }\right) \le C\!\left( \Vert {\text {curl}}\,{\textbf{v}}\Vert +\Vert {\textbf{v}}\Vert _{{\textbf{H}}( {\text {curl}},\varOmega ),k}\right) . \nonumber \\ \end{aligned}$$

The combination of (8.24) and (8.25) shows that ${\textbf{v}}\in {\textbf{H}}^{1}( \varOmega )$ and

$$\begin{aligned} \left\| {\textbf{v}}\right\| _{{\textbf{H}}^{1}\left( \varOmega \right) }\le C\left\| {\textbf{v}}\right\| _{{\textbf{H}}\left( {\text {curl}},\varOmega \right) ,k}\le C\left\| {\textbf{v}}\right\| _{{\text {imp}},k}. \end{aligned}$$

Since we have trivially $\left| k\right| \left\| {\textbf{v}} \right\| \le \left\| {\textbf{v}}\right\| _{{\textbf{H}}\left( {\text {curl}},\varOmega \right) ,k}$, the assertion follows. $\square$

We also need the following subspace of ${\textbf{V}}_{k,0}$ given by

8.26 $$\begin{aligned} {{\textbf{V}}}_{k,0,h}:=\left\{ {{\textbf{v}}}\in {{\textbf{V}}}_{k,0} \mid {\text {curl}}\,{{\textbf{v}}}\in {\text {curl}}\,{{\textbf{X}}} _{h}\right\} . \end{aligned}$$

The operator $\varPi _{p}^{{\text {curl}},s}$ in (8.13), (8.14) has (*p*-optimal) approximation properties in $\Vert \cdot \Vert _{{\text {curl}},\varOmega ,k}$ as it has simultaneously *p*-optimal approximation properties in $L^{2}$ and $H^{1}$. However, it is not a projection and does not have the commuting diagram property. Since this is needed for the estimate of the consistency term in Sect. 9.2 we employ operators, $\varPi _{p}^{{\text {curl}},c}$, $\varPi _{p} ^{{\text {div}},c}$, which enjoy these properties. They were constructed in [36] in an element-by-element fashion and used in [39, Thm. 8.2]. The choice $\varPi _{h}^{E}:{\textbf{V}}_{k,0,h}+{\textbf{X}}_{h}\rightarrow {\textbf{X}}_{h}$ as $\varPi _{p}^{{\text {curl}},c}$ and the companion operator $\varPi _{h} ^{F}:H({\text {div}},\varOmega )\cap \prod _{K\in {{\mathcal {T}}}_{h}}{{\textbf{H}} }^{1}({\text {div}},K)\rightarrow {\text {curl}}\,{\textbf{X}}_{h}$ as $\varPi _{p}^{{\text {div}},c}$ allows us to derive quantitative convergence estimates in Sect. 9.

#### Lemma 8.8

The operators $\varPi _{h}^{E}:=\varPi _{p}^{{\text {curl}},c}$ and $\varPi _{h} ^{F}:=\varPi _{p}^{{\text {div}},c}$ of [39] satisfy the following properties: $\varPi _{h}^{E}:{\textbf{V}}_{k,0,h}+{\textbf{X}}_{h}\rightarrow {{\textbf{X}}}_{h}$ and $\varPi _{h}^{F}:H({\text {div}},\varOmega )\cap \prod _{K\in {{\mathcal {T}}}_{h} }{{\textbf{H}}}^{1}({\text {div}},K)\rightarrow {\text {curl}}\,{\textbf{X}}_{h}$ are linear mappings with

(i) $\varPi _{h}^{E}$ is a projection, i.e., the restriction $\left. \varPi _{h}^{E}\right| _{{\textbf{X}}_{h}}$ is the identity on ${\textbf{X}}_{h}$.
(ii) The operators $\varPi _{h}^{E}$ and $\varPi _{h}^{F}$ have the commuting property: ${\text {curl}}\,\varPi _{h}^{E}=\varPi _{h}^{F}{\text {curl}}$.

#### Proof

Since $\varPi _{p}^{{\text {curl}},c}$ is based on an element-by-element construction it is well defined on ${\textbf{H}}({\text {curl}},\varOmega )\cap {\prod _{K\in {\mathcal {T}}_{h}}}{\textbf{H}}^{1}({\text {curl}},K)$. Since ${\textbf{V}}_{k,0,h}+{\textbf{X}}_{h}$ is a subspace of this space, the mapping properties follow. The projection property of $\varPi _{h}^{E}$ and the commuting property of $\varPi _{h}^{E}$ and $\varPi _{h}^{F}$ are proved in [36, Thm. 2.10, Rem. 2.11]. $\square$

## Stability and Convergence of the Galerkin Discretization

The wavenumber-explicit stability and convergence analysis for Maxwell’s equations with transparent boundary conditions has been developed recently in [39] and generalizes the theory in [41, Sec. 7.2]. A “roadmap” for the convergence proof of [39] is given in [39, Sec. 1.1–1.3]. In the present analysis, we follow this “roadmap” taking into account the change in boundary conditions from transparent boundary conditions to impedance boundary conditions. A key role is played by the term ${ \left( \!\left( \hspace{-0.50003pt} {\textbf{u}},{\textbf{v}} \hspace{-1.00006pt}\right) \!\right) }_k = A_{k}( {\textbf{u}},{\textbf{v}}) - \left( {\text {curl}} \textbf{u,}\,{\text {curl}}\,{\textbf{v}}\right)$ from (2.42), which includes the boundary conditions. This sesquilinear form determines the space ${\textbf{V}}_{k,0}$ (see (8.22)) and the regular decomposition in Def. 9.2 ahead and its properties differentiate the present case of impedance boundary conditions from the transparent boundary condition case. Compared to the case of transparent boundary conditions, the present impedance boundary conditions case is simpler in that fewer approximation quantities $\eta ^{{\text {alg}}}_j$, ${{\tilde{\eta }}}_j^{{\text {alg}}}$ are required in the analysis.

In this section, we develop a stability and convergence theory for Maxwell’s equations with impedance boundary conditions, see Sect. 2.5. Recall the definition of the sesquilinear form ${ \left( \!\left( \hspace{-0.50003pt} \cdot ,\cdot \hspace{-1.00006pt}\right) \!\right) }_k$ of (2.42) and of the norm $\left\| \cdot \right\| _{k,+}$ in Definition 2.5.

We introduce the quantity $\delta _{k}:{\textbf{X}}_{{\text {imp}} }\rightarrow {\mathbb {R}}$ by $$\delta _{k}\left( {\textbf{0}}\right) :=0\ $$and for ${\textbf{w}}\in {\textbf{X}}_{{\text {imp}}}\backslash \left\{ {\textbf{0}} \right\}$ by

9.1 $$\begin{aligned} \delta _{k}({\textbf{w}}):=\sup _{{\textbf{v}}_{h}\in {\textbf{X}}_{h}\backslash \left\{ {\textbf{0}} \right\} }\left( 2\frac{\vert { \left( \!\left( \hspace{-0.50003pt} {\textbf{w}},{\textbf{v}}_{h} \hspace{-1.00006pt}\right) \!\right) }_k\vert }{\Vert {\textbf{w}}\Vert _{{\text {imp}},k} \Vert {\textbf{v}}_{h}\Vert _{{\text {imp}},k}}\right) , \end{aligned}$$

which will play the important role of a consistency term.

### Proposition 9.1

(quasi-optimality) Let ${\textbf{E}}\in {\textbf{X}} _{{\text {imp}}}$ and ${\textbf{E}}_{h}\in {\textbf{X}}_{h}$ satisfy

$$\begin{aligned} A_{k}( {\textbf{E}}-{\textbf{E}}_{h},{\textbf{v}}_{h}) =0\qquad \forall \,{\textbf{v}}_{h}\in {\textbf{X}}_{h}. \end{aligned}$$

Assume that $\delta _{k}( {\textbf{e}}_{h}) <1$ for ${\textbf{e}}_{h}:={\textbf{E}}-{\textbf{E}}_{h}$. Then, ${\textbf{e}}_{h}$ satisfies, for all ${\textbf{w}}_{h}\in {\textbf{X}}_{h}$, the quasi-optimal error estimate

$$\begin{aligned} \left\| {\textbf{e}}_{h}\right\| _{{\text {imp}},k}\le \frac{1+\delta _{k}( {\textbf{e}}_{h}) }{1-\delta _{k}( {\textbf{e}}_{h}) }\left\| {\textbf{E}}-{\textbf{w}}_{h}\right\| _{{\text {imp}},k}. \end{aligned}$$

### Proof

The definitions of the sesquilinear forms $A_{k}$ and ${ \left( \!\left( \hspace{-0.50003pt} \cdot ,\cdot \hspace{-1.00006pt}\right) \!\right) }_k$ imply

9.2 $$\begin{aligned} \left\| {\textbf{e}}_{h}\right\| _{_{{\text {imp}},k}} ^{2}=\left| A_{k}( {\textbf{e}}_{h},{\textbf{e}}_{h}) +2 { \left( \!\left( \hspace{-0.50003pt} {\textbf{e}}_{h},{\textbf{e}}_{h} \hspace{-1.00006pt}\right) \!\right) }_k\right| . \end{aligned}$$

We employ Galerkin orthogonality for the first term in (9.2) to obtain for any ${\textbf{w}}_{h}\in {\textbf{X}}_{h}$

$$\begin{aligned} \left\| {\textbf{e}}_{h}\right\| _{{\text {imp}},k}^{2}\le & {} \bigl \vert A_{k}( {\textbf{e}}_{h},{\textbf{E}}-{\textbf{w}}_{h}) +2 { \left( \!\left( \hspace{-0.50003pt} {\textbf{e}}_{h},{\textbf{E}}-{\textbf{w}}_{h} \hspace{-1.00006pt}\right) \!\right) }_k\bigr \vert \\{} & {} +\delta _{k}\left( {{\textbf{e}}}_{h}\right) \bigl \Vert {\textbf{e}}_{h}\bigr \Vert _{{\text {imp}},k}\!\!\!\! \underset{\le \left\| {\textbf{e}}_{h}\right\| _{{\text {imp}},k}+\left\| {\textbf{E}}-{\textbf{w}}_{h}\right\| _{{\text {imp}},k}}{\underbrace{\left\| {\textbf{E}}_{h}-{\textbf{w}}_{h}\right\| _{{\text {imp}},k} }}. \end{aligned}$$

We write $A_{k}$ in the form (2.43) so that

9.3 $$\begin{aligned} \left( 1-\delta _{k}( {\textbf{e}}_{h}) \right) \left\| {\textbf{e}} _{h}\right\| _{{\text {imp}},k}^{2}&\le \left| \left( {\text {curl}}\,{\textbf{e}}_{h},{\text {curl}}\left( {\textbf{E}} -{\textbf{w}}_{h}\right) \right) + { \left( \!\left( \hspace{-0.50003pt} {\textbf{e}}_{h},{\textbf{E}}-{\textbf{w}}_{h} \hspace{-1.00006pt}\right) \!\right) }_k \right| \nonumber \\&\quad +\delta _{k}( {{\textbf{e}}}_{h}) \left\| {\textbf{e}}_{h}\right\| _{{\text {imp}},k}\left\| {\textbf{E}}-{\textbf{w}}_{h}\right\| _{{\text {imp}},k}. \end{aligned}$$

The sesquilinear form ${ \left( \!\left( \hspace{-0.50003pt} \cdot ,\cdot \hspace{-1.00006pt}\right) \!\right) }_k$ is continuous, and we have

9.4 $$\begin{aligned} \bigl | { \left( \!\left( \hspace{-0.50003pt} {\textbf{u}},{\textbf{v}} \hspace{-1.00006pt}\right) \!\right) }_k \bigr | \le \left\| {\textbf{u}}\right\| _{k,+}\left\| {\textbf{v}}\right\| _{k,+}\qquad \forall {\textbf{u}},{\textbf{v}}\in {\textbf{X}}_{{\text {imp}}}. \end{aligned}$$

Hence,

$$\begin{aligned} \left( 1-\delta _{k}( {\textbf{e}}_{h}) \right) \left\| {\textbf{e}} _{h}\right\| _{{\text {imp}},k}^{2}&\le \left\| {\textbf{e}} _{h}\right\| _{{\text {imp}},k}\left\| {\textbf{E}}-{\textbf{w}} _{h}\right\| _{{\text {imp}},k} \\&\quad +\delta _{k}( {{\textbf{e}}}_{h}) \left\| {\textbf{e}}_{h}\right\| _{{\text {imp}},k}\left\| {\textbf{E}}-{\textbf{w}}_{h}\right\| _{{\text {imp}},k}, \end{aligned}$$

and the assertion follows. $\square$

### Splitting of the Consistency Term

We introduce continuous and discrete Helmholtz decompositions that are adapted to the problem under consideration.

#### Definition 9.2

On ${\textbf{v}}\in {\textbf{X}}_{{\text {imp}}}$ the *Helmholtz splittings*

9.5a $$\begin{aligned} {\textbf{v}}&=\varPi _{k}^{{\text {curl}}}{\textbf{v}}+\varPi _{k}^{\nabla }{\textbf{v}}, \end{aligned}$$

9.5b $$\begin{aligned} {\textbf{v}}&=\varPi _{k,h}^{{\text {curl}}}{\textbf{v}}+\varPi _{k,h}^{\nabla }{\textbf{v}} \end{aligned}$$

are given via operators $\varPi _{k}^{\nabla }$, $\varPi _{k}^{{\text {curl}}}$ and their discrete counterparts $\varPi _{k,h}^{\nabla }$, $\varPi _{k,h} ^{{\text {curl}}}$ by seeking $\varPi _{k}^{\nabla }{\textbf{v}}\in \nabla H_{{\text {imp}}}^{1}( \varOmega )$ and $\varPi _{k,h}^{\nabla }{\textbf{v}} \in \nabla S_{h}$ such that

9.6a $$\begin{aligned} { \left( \hspace{-2.5pt}\left( \hspace{-0.50003pt} \varPi _{k}^{\nabla }{\textbf{v}},\nabla \psi \hspace{-1.00006pt}\right) \hspace{-2.5pt}\right) _k }&= { \left( \!\left( \hspace{-0.50003pt} {\textbf{v}},\nabla \psi \hspace{-1.00006pt}\right) \!\right) }_k\qquad \forall \psi \in H_{{\text {imp}}}^{1}( \varOmega ) , \end{aligned}$$

9.6b $$\begin{aligned} { \left( \hspace{-2.5pt}\left( \hspace{-0.50003pt} \varPi _{k,h}^{\nabla }{\textbf{v}},\nabla \psi \hspace{-1.00006pt}\right) \hspace{-2.5pt}\right) _k }&={ \left( \!\left( \hspace{-0.50003pt} {\textbf{v}},\nabla \psi \hspace{-1.00006pt}\right) \!\right) }_k\qquad \forall \psi \in S_{h}. \end{aligned}$$

The operators $\varPi _{k}^{{\text {curl}}}{\textbf{v}}$, $\varPi _{k,h} ^{{\text {curl}}}{\textbf{v}}$ are then given via the relations (9.5).

It is easy to see (cf. (2.45)) that

9.7a $$\begin{aligned} { \left( \hspace{-2.5pt}\left( \hspace{-0.50003pt} \nabla \psi ,\varPi _{-k}^{\nabla }{\textbf{v}} \hspace{-1.00006pt}\right) \hspace{-2.5pt}\right) _k }&={ \left( \!\left( \hspace{-0.50003pt} \nabla \psi ,{\textbf{v}} \hspace{-1.00006pt}\right) \!\right) }_k\qquad \forall \psi \in H_{{\text {imp}}}^{1}( \varOmega ) , \end{aligned}$$

9.7b $$\begin{aligned} { \left( \hspace{-2.5pt}\left( \hspace{-0.50003pt} \nabla \psi ,\varPi _{-k,h}^{\nabla }{\textbf{v}} \hspace{-1.00006pt}\right) \hspace{-2.5pt}\right) _k }&={ \left( \!\left( \hspace{-0.50003pt} \nabla \psi ,{\textbf{v}} \hspace{-1.00006pt}\right) \!\right) }_k\qquad \forall \psi \in S_{h}. \end{aligned}$$

Solvability of these equations follows trivially from the Lax-Milgram lemma as can be seen from the following lemma.

#### Lemma 9.3

Problems (9.6) have unique solutions, which satisfy

$$\begin{aligned} \bigl \Vert \varPi _{k}^{\nabla }{\textbf{v}}\bigr \Vert _{{\text {imp}} ,k}+\bigr \Vert \varPi _{k}^{{\text {curl}}}{\textbf{v}}\bigr \Vert _{{\text {imp}},k}&\le C\bigl \Vert {\textbf{v}}\bigr \Vert _{{\text {imp}},k},\\ \bigl \Vert \varPi _{k,h}^{\nabla }{\textbf{v}}\bigr \Vert _{{\text {imp}} ,k}+\bigl \Vert \varPi _{k,h}^{{\text {curl}}}{\textbf{v}}\bigr \Vert _{{\text {imp}},k}&\le C\bigl \Vert {\textbf{v}}\bigr \Vert _{{\text {imp}},k}. \end{aligned}$$

#### Proof

We first consider the continuous problem (9.6a). Taking $\sigma =\exp \left( \left( {\text {sign}}k\right) {\text {i}}\, \frac{\pi }{4}\right)$ we obtain coercivity as in the proof of Theorem 4.3 (i) via

$$\begin{aligned} {\text {Re}}{ \left( \!\left( \hspace{-0.50003pt} \nabla \psi ,\sigma \nabla \psi \hspace{-1.00006pt}\right) \!\right) }_k= & {} 2^{-1/2}\! \left[ k^{2}\left( \nabla \psi ,\nabla \psi \right) +\left| k\right| \left( \nabla _{\varGamma }\psi ,\nabla _{\varGamma } \psi \right) _{{\textbf{L}}^{2}( \varGamma ) }\right] \! \\= & {} 2^{-1/2} \Vert \nabla \psi \Vert _{{\text {imp}},k}^{2}. \end{aligned}$$

The continuity follows from (9.4):

9.8 $$\begin{aligned} \left| { \left( \!\left( \hspace{-0.50003pt} \nabla \varphi ,\nabla \psi \hspace{-1.00006pt}\right) \!\right) }_k\right| \le \left\| \nabla \varphi \right\| _{k,+}\left\| \nabla \psi \right\| _{k,+}=\left\| \nabla \varphi \right\| _{{\text {imp}},k}\left\| \nabla \psi \right\| _{{\text {imp}},k}. \end{aligned}$$

This implies existence, uniqueness, and the *a priori* estimate

$$\begin{aligned} \bigl \Vert \varPi _{k}^{\nabla }{\textbf{v}}\bigr \Vert _{{\text {imp}},k} \le \sqrt{2}\left\| {\textbf{v}}\right\| _{{\text {imp}},k}. \end{aligned}$$

The estimate of $\varPi _{k}^{{\text {curl}}}{\textbf{v}}$ follows by a triangle inequality. Since the coercivity and continuity estimates are inherited by the finite dimensional subspace $\nabla S_{h}$, well-posedness also follows on the discrete level. The estimates for the other operators follow verbatim. $\square$

The principal splitting of the consistency term $\delta _{k}$ in (9.1) is introduced next. We write

9.9 $$\begin{aligned}&{ \left( \!\left( \hspace{-0.50003pt} {\textbf{e}}_{h},{\textbf{v}}_{h} \hspace{-1.00006pt}\right) \!\right) }_k \nonumber \\&\quad = \underset{=:T_{1}}{\underbrace{{ \left( \hspace{-2.5pt}\left( \hspace{-0.50003pt} {\textbf{e}}_{h},\left( \varPi _{-k,h}^{{\text {curl}}}-\varPi _{-k} ^{{\text {curl}}}\right) {\textbf{v}}_{h} \hspace{-1.00006pt}\right) \hspace{-2.5pt}\right) _k }}}+\underset{=:T_{2}}{\underbrace{{ \left( \hspace{-2.5pt}\left( \hspace{-0.50003pt} {\textbf{e}}_{h},\varPi _{-k}^{{\text {curl}}}{\textbf{v}}_{h} \hspace{-1.00006pt}\right) \hspace{-2.5pt}\right) _k }}}+\underset{=:T_{3}}{\underbrace{{ \left( \hspace{-2.5pt}\left( \hspace{-0.50003pt} {\textbf{e}}_{h},\varPi _{-k,h}^{\nabla }{\textbf{v}}_{h} \hspace{-1.00006pt}\right) \hspace{-2.5pt}\right) _k }}}. \end{aligned}$$

Galerkin orthogonality implies ${ \left( \!\left( \hspace{-0.50003pt} {\textbf{e}}_{h},\varPi _{-k,h}^{\nabla }{\textbf{v}}_{h} \hspace{-1.00006pt}\right) \!\right) }_k=0$, i.e., $T_{3}=0$.

### Consistency Analysis: The Term ${\textbf{T}}_{1}$ in (9.9)

The continuity of the sesquilinear form ${ \left( \!\left( \hspace{-0.50003pt} \cdot ,\cdot \hspace{-1.00006pt}\right) \!\right) }_k$ (cf. (9.4)) implies

9.10 $$\begin{aligned} \left| T_{1}\right| \le \left\| {\textbf{e}}_{h}\right\| _{k,+}\bigl \Vert \bigl ( \varPi _{-k,h}^{{\text {curl}}}-\varPi _{-k} ^{{\text {curl}}}\bigr ) {\textbf{v}}_{h}\bigr \Vert _{k,+}. \end{aligned}$$

The definition of the discrete and continuous Helmholtz decomposition applied to a discrete function ${\textbf{v}}_{h}$ leads to (cf. Def. 9.2, (9.7))

9.11 $$\begin{aligned} { \left( \hspace{-2.5pt}\left( \hspace{-0.50003pt} \nabla \psi _{h},\left( \varPi _{-k,h}^{{\text {curl}}}-\varPi _{-k} ^{{\text {curl}}}\right) {\textbf{v}}_{h} \hspace{-1.00006pt}\right) \hspace{-2.5pt}\right) _k }=0\quad \forall \psi _{h}\in S_{h}. \end{aligned}$$

We use (9.5) to get ${\text {curl}}\varPi _{-k} ^{{\text {curl}}}={\text {curl}}\varPi _{-k,h}^{{\text {curl}} }={\text {curl}}$ on ${\textbf{X}}_{h}$ and thus

9.12 $$\begin{aligned}&{\text {curl}}\bigl ( \varPi _{-k,h}^{{\text {curl}}}{{\textbf{v}}} _{h}-\varPi _{h}^{E}\varPi _{-k}^{{\text {curl}}}{{\textbf{v}}}_{h}\bigr )\nonumber \\&\quad \overset{\text {Lem. 8.8(ii) }}{=} {\text {curl}}\bigl ( \varPi _{-k,h}^{{\text {curl}}}{{\textbf{v}}} _{h}\bigr ) -\varPi _{h}^{F}{\text {curl}}\bigl ( \varPi _{-k} ^{{\text {curl}}}{{\textbf{v}}}_{h}\bigr ) = {\text {curl}}\,{{\textbf{v}}}_{h}-\varPi _{h}^{F}{\text {curl}}\,{{\textbf{v}}}_{h}\nonumber \\&\quad \overset{\text {Lem. 8.8(ii)}}{=} {\text {curl}}\,{{\textbf{v}}}_{h}-{\text {curl}}\,\varPi _{h} ^{E}{{\textbf{v}}}_{h}\overset{\text {Lem. 8.8(i) } }{=}{\text {curl}}\left( {{\textbf{v}}}_{h}-{{\textbf{v}}}_{h}\right) =0. \end{aligned}$$

By the exact sequence property (8.8), the observation (9.12) implies that $\varPi _{-k,h}^{{\text {curl}} }{\textbf{v}}_{h}-\varPi _{h}^{E}\varPi _{-k}^{{\text {curl}}}{\textbf{v}} _{h}=\nabla \psi _{h}$ for some $\psi _{h}\in S_{h}$ and therefore

9.13 $$\begin{aligned} \bigl ( \varPi _{-k,h}^{{\text {curl}}}-\varPi _{-k}^{{\text {curl}} }\bigr ) {\textbf{v}}_{h}=\nabla \psi _{h}+\bigl ( ( \varPi _{h}^{E}-I) \varPi _{-k}^{{\text {curl}}}\bigr ) {\textbf{v}}_{h}. \end{aligned}$$

For the second factor in (9.10) we get by the Galerkin orthogonality (9.11) and (9.13)

$$\begin{aligned}&\left\| \left( \varPi _{-k,h}^{{\text {curl}}}-\varPi _{-k} ^{{\text {curl}}}\right) {\textbf{v}}_{h}\right\| _{k,+}^{2} \\&\quad = {\text {Re}}\,{ \left( \hspace{-2.5pt}\left( \hspace{-0.50003pt} \bigl ( \varPi _{h}^{E}-I) \varPi _{-k}^{{\text {curl}}} {\textbf{v}}_{h},( \varPi _{-k,h}^{{\text {curl}}}-\varPi _{-k} ^{{\text {curl}}}\bigr ) {\textbf{v}}_{h} \hspace{-1.00006pt}\right) \hspace{-2.5pt}\right) _k }\\&\qquad +\left( {\text {sign}}\,k\right) {\text {Im}}\,{ \left( \hspace{-2.5pt}\left( \hspace{-0.50003pt} \ \bigl ( \varPi _{h}^{E}-I\bigr ) \varPi _{-k}^{{\text {curl}}} {\textbf{v}}_{h},\bigl ( \varPi _{-k,h}^{{\text {curl}}}-\varPi _{-k} ^{{\text {curl}}}\bigr ) {\textbf{v}}_{h} \hspace{-1.00006pt}\right) \hspace{-2.5pt}\right) _k }\\&\quad \le 2\bigl \Vert \bigl ( \varPi _{h}^{E}-I\bigr ) \varPi _{-k} ^{{\text {curl}}}{\textbf{v}}_{h}\bigr \Vert _{k,+}\bigl \Vert \bigl ( \varPi _{-k,h}^{{\text {curl}}}-\varPi _{-k}^{{\text {curl}} }\bigr ) {\textbf{v}}_{h}\bigr \Vert _{k,+} \end{aligned}$$

so that

$$\begin{aligned} \bigl \Vert \bigl ( \varPi _{-k,h}^{{\text {curl}}}-\varPi _{-k} ^{{\text {curl}}}\bigr ) {\textbf{v}}_{h}\bigr \Vert _{k,+} \le 2\bigr \Vert \bigl ( \varPi _{h}^{E}-I\bigr ) \varPi _{-k} ^{{\text {curl}}} {\textbf{v}}_{h}\bigr \Vert _{k,+}. \end{aligned}$$

This leads to the estimate of $T_{1}$

$$\begin{aligned} \left| T_{1}\right| \le 2\left\| {\textbf{e}}_{h}\right\| _{k,+}\bigl \Vert \bigl ( I-\varPi _{h}^{E}\bigr ) \varPi _{-k}^{{\text {curl}} }{\textbf{v}}_{h}\bigr \Vert _{k,+}. \end{aligned}$$

We set^9^

9.14 $$\begin{aligned} \eta _{6}^{{\text {alg}}}:=\eta _{6}^{{\text {alg}}}\bigl ( {\textbf{X}}_{h},\varPi _{h}^{E}\bigr ):=\sup _{\begin{array}{c} {\textbf{w}}\in {\textbf{V}}_{-k,0}\backslash \left\{ {\textbf{0}} \right\} :\\ {\text {curl}}\,{{\textbf{w}}}\in {\text {curl}}\,{{\textbf{X}}}_{h} \end{array}}\frac{\bigl \Vert {\textbf{w}}-\varPi _{h}^{E}{\textbf{w}}\bigr \Vert _{k,+}}{\Vert {\textbf{w}}\Vert _{{\textbf{H}}^{1}( \varOmega ) }} \end{aligned}$$

and obtain

9.15 $$\begin{aligned} \left| T_{1}\right|&\le 2\Vert {\textbf{e}}_{h}\Vert _{k,+}\eta _{6}^{{\text {alg}}}\bigl \Vert \varPi _{-k}^{{\text {curl}} }{\textbf{v}}_{h}\bigr \Vert _{{\textbf{H}}^{1}( \varOmega ) } \le 2C\left\| {\textbf{e}}_{h}\right\| _{k,+}\eta _{6}^{{\text {alg}} }\bigl \Vert \varPi _{-k}^{{\text {curl}}}{\textbf{v}}_{h}\bigr \Vert _{{\text {imp}},k} \nonumber \\&\le {\tilde{C}}\left\| {\textbf{e}}_{h}\right\| _{k,+}\eta _{6}^{{\text {alg}}}\left\| {\textbf{v}}_{h}\right\| _{{\text {imp}},k}. \end{aligned}$$

#### *hp*-Analysis of $T_{1}$

In [39, (4.72)] it was proved that for our choice $\varPi _{h}^{E}:=\varPi _{p}^{{\text {curl}},c}$ with $\varPi _{p} ^{{\text {curl}},c}$ as in [36, 39, §8] (see Lem. 8.8), one has

9.16 $$\begin{aligned} \sup _{\begin{array}{c} {\textbf{w}}\in {\textbf{V}}_{-k,0}\backslash \left\{ {\textbf{0}} \right\} :\\ {\text {curl}}\,{{\textbf{w}}}\in {\text {curl}}\,{{\textbf{X}}}_{h} \end{array}}\frac{\vert k\vert \bigl \Vert {\textbf{w}}-\varPi _{h}^{E}{\textbf{w}}\bigr \Vert }{\left\| {\textbf{w}}\right\| _{{\textbf{H}}^{1}( \varOmega ) }}\le C\frac{\left| k\right| h}{p}. \end{aligned}$$

For the boundary term in the norm $\Vert \cdot \Vert _{k,+}$ we study the approximation properties of the operator $\varPi _{p}^{{\text {curl}},c}$ of [36] on the boundary of the reference tetrahedron ${\widehat{K}}$ more carefully.

##### Lemma 9.4

Let ${\widehat{\varPi }}_{p}^{{\text {curl}},3d}$ be the operator introduced in [36]. For all ${\textbf{u}}\in {\textbf{H}}^{1}( {\widehat{K}})$ with ${\text {curl}}\,{\textbf{u}} \in ( {\mathcal {P}}_{p}( {\widehat{K}}) ) ^{3}$, there holds with the tangential component operator $\varPi _{T,\partial {\widehat{K}}}$

$$\begin{aligned} \bigl \Vert \varPi _{T,\partial {\widehat{K}}} \bigl ( {\textbf{u}}-{\widehat{\varPi }} _{p}^{{\text {curl}},3d}{\textbf{u}}\bigr ) \bigr \Vert _{{\textbf{L}} ^{2}(\partial {\widehat{K}}) }\le Cp^{-1/2}\left\| {\textbf{u}}\right\| _{H^{1}( {\widehat{K}}) }. \end{aligned}$$

##### Proof

We follow the proof of [36, Lem. 6.15] and employ the notation used there. From [36, proof of Lem. 6.15] and [36, (6.42)], we can decompose ${{\textbf{u}}}=\nabla \varphi +{{\textbf{v}}}$ with

9.17 $$\begin{aligned} \Vert \varphi \Vert _{H^{2}({\widehat{K}})}+\Vert {{\textbf{v}}}\Vert _{{{\textbf{H}} }^{1}({\widehat{K}})}\le C\Vert {{\textbf{u}}}\Vert _{{{\textbf{H}}}^{1}(\widehat{K})}. \end{aligned}$$

Since ${\text {curl}}\,{\textbf{u}}\in ({\mathcal {P}}_{p}(\widehat{K}))^{3}$ the decomposition is such that (cf. [36, Proof of Lemma 6.15]) we have ${\textbf{v}}-{\widehat{\varPi }}_{p}^{{\text {curl}},3d}{\textbf{v}}=0$. We conclude

$$\begin{aligned} {\textbf{u}}-{\widehat{\varPi }}_{p}^{{\text {curl}},3d}{\textbf{u}} ={\textbf{v}}+\nabla \varphi -{\widehat{\varPi }}_{p}^{{\text {curl}},3d}({\textbf{v}}+\nabla \varphi )=\nabla (\varphi -{\widehat{\varPi }}_{p+1} ^{{\text {grad}},3d}\varphi ) \end{aligned}$$

with ${\widehat{\varPi }}_{p+1}^{{\text {grad}},3d}$ as in [36]. The construction of the projection-based interpolation operators ${\widehat{\varPi }}_{p}^{{\text {curl}},3d}$, ${\widehat{\varPi }}_{p}^{{\text {grad}},3d}$ is such that facewise, they reduce to corresponding 2D operators. That is, for each face $f\subset \partial {\widehat{K}}$ we have

$$\begin{aligned} \varPi _{T,\partial {\widehat{K}}}\bigl ( {\textbf{u}}-{\widehat{\varPi }} _{p}^{{\text {curl}},3d}{\textbf{u}}\bigr ) \vert _{f}= \varPi _{T,\partial {\widehat{K}}}\bigl ( \nabla \bigl ( \varphi -{\widehat{\varPi }} _{p+1}^{{\text {grad}},3d}\varphi \bigr ) \bigr ) \vert _{f}=\nabla _{{f}}\bigl ( {\textbf{I}}-{\widehat{\varPi }}_{p+1}^{{\text {grad}},2d}\bigr ) \bigl ( \varphi \vert _{f}\bigr ). \end{aligned}$$

We apply [36, Thm. 2.13] to obtain

$$\begin{aligned} \bigl \Vert \varPi _{T,\partial {\widehat{K}}}\bigl ( {\textbf{u}}-{\widehat{\varPi }} _{p}^{{\text {curl}},3d}{\textbf{u}}\bigr ) \bigr \Vert _{{\textbf{L}} ^{2}(f)}&=\bigl \Vert \nabla _{{f}}\bigl ( {\textbf{I}}-{\widehat{\varPi }} _{p+1}^{{\text {grad}},2d}\bigr ) \varphi \vert _{f}\bigr ) \bigr \Vert _{{\textbf{L}}^{2}(f)}\le Cp^{-1/2}\Vert \varphi \Vert _{H^{3/2}(f)}\\&\le Cp^{-1/2}\Vert \varphi \Vert _{H^{2}({\widehat{K}})} \overset{(\text {9.17})}{\le }Cp^{-1/2}\Vert {\textbf{u}}\Vert _{H^{1}({\widehat{K}})}. \end{aligned}$$

$$\square $$

For the boundary part of $\left\| {\textbf{w}}\right\| _{{\text {imp}},k}$ of a ${\textbf{w}}\in {\textbf{V}}_{-k,0}$ with ${\text {curl}}\,{{\textbf{w}}}\in {\text {curl}}\,{{\textbf{X}}}_{h}$, we get, by applying a scaling argument to Lemma 9.4:

9.18 $$\begin{aligned} | k| \bigl \Vert \varPi _{T}\bigl ( {\textbf{w}}-\varPi _{h} ^{E}{\textbf{w}}\bigr ) \bigr \Vert _{{\textbf{L}}^{2}(\varGamma )}^{2}&=| k| \sum _{\begin{array}{c} K\in {\mathcal {T}}_{h}\\ \left| \overline{K}\cap \varGamma \right|>0 \end{array}}\bigl \Vert \varPi _{T}\bigl ( {\textbf{w}}-\varPi _{h} ^{E}{\textbf{w}}\bigr ) \bigr \Vert _{{\textbf{L}}^{2}(K\cap \varGamma )} ^{2} \nonumber \\&\le C\frac{|k| h}{p}\sum _{\begin{array}{c} K\in {\mathcal {T}}_{h}\\ \left| \overline{K}\cap \varGamma \right| >0 \end{array}}\Vert {\textbf{w}}\Vert _{{\textbf{H}}^{1}(K)}^{2}\le C\frac{ |k| h}{p}\Vert {\textbf{w}}\Vert _{{\textbf{H}}^{1}(\varOmega )}^{2}. \nonumber \\ \end{aligned}$$

The combination of (9.16) with (9.18) leads to

9.19 $$\begin{aligned} \eta _{6}^{{\text {alg}}}\le C\left( \frac{|k | h}{p}\right) ^{1/2} \left( 1+\left( \frac{\left| k\right| h}{p}\right) ^{1/2}\right) . \end{aligned}$$

### Consistency Analysis: The Term ${\textbf{T}}_{2}$ in (9.9)

Recall the definition of $T_{2}={ \left( \!\left( \hspace{-0.50003pt} {\textbf{e}}_{h},{\textbf{v}}_{0} \hspace{-1.00006pt}\right) \!\right) }_k$ with ${\textbf{v}}_{0}:=\varPi _{-k}^{{\text {curl}}} {\textbf{v}}_{h} = ({\text {I}} - \varPi _{-k}^{\nabla }) {\textbf{v}}_h$. The function ${\textbf{v}}_{0}$ belongs to ${\textbf{X}} _{{\text {imp}}}$ and by combining (9.5a) and (9.6a) we find that ${\textbf{v}}_{0}$ belongs to ${\textbf{V}} _{-k,0}$. Proposition 8.7 implies ${\textbf{v}}_{0}\in {\textbf{H}}^{1}(\varOmega )$ and

9.20 $$\begin{aligned} \Vert {\textbf{v}}_{0}\Vert _{{\textbf{H}}^{1}(\varOmega )}\le |k| \Vert {\textbf{v}}_{0}\Vert _{{\textbf{H}}^{1}(\varOmega ),k}\le C\Vert {\textbf{v}}_{0}\Vert _{{\textbf{H}} ({\text {curl}},\varOmega ),k}\le C\Vert {\textbf{v}}_{0}\Vert _{{\text {imp}},k}. \end{aligned}$$

The characterization (8.23) of ${\textbf{V}}_{-k,0}$ implies

9.21 $$\begin{aligned} \mathop {{\text {div}}}\nolimits _{\varGamma }\,( {\textbf{v}}_{0}) _{T}={\text {i}}\,k\langle {\textbf{v}}_{0},{\textbf{n}}\rangle . \end{aligned}$$

To estimate the term $T_{2}$, we consider the dual problem: Given ${\textbf{v}}_{0}\in {\textbf{V}}_{-k,0}$, find ${\textbf{z}}\in {\textbf{X}} _{{\text {imp}}}$ such that

$$\begin{aligned} A_{k}({\textbf{w}},{\textbf{z}})={ \left( \!\left( \hspace{-0.50003pt} {\textbf{w}},{\textbf{v}}_{0} \hspace{-1.00006pt}\right) \!\right) }_k\quad \forall {\textbf{w}}\in {\textbf{X}}_{{\text {imp}}}. \end{aligned}$$

The operator ${\mathcal {N}}_{-k}:{\textbf{V}}_{-k,0}\rightarrow {\textbf{X}} _{{\text {imp}}}$ is defined by ${\mathcal {N}}_{-k}{\textbf{v}} _{0}:={\textbf{z}}$. The strong formulation is given by

9.22 $$\begin{aligned} {\mathcal {L}}_{\varOmega ,-k}{\textbf{z}}&=k^{2}{\textbf{v}}_{0} \quad \text {in }\varOmega ,&{\mathcal {B}}_{ \varGamma ,-k}{\textbf{z}}&=-{\text {i}}\,k\left( {\textbf{v}}_{0}\right) _{T} \quad \text {on }\varGamma . \end{aligned}$$

Hence, ${\mathcal {N}}_{-k}{\textbf{v}}_{0}={\mathcal {S}}_{\varOmega ,-k} ^{{\text {MW}}}\left( k^{2}{\textbf{v}}_{0},-{\text {i}}\,k\left( {\textbf{v}}_{0}\right) _{T}\right)$. By Galerkin orthogonality satisfied by ${\textbf{e}}_{h}$, we have for any ${\textbf{w}}_{h}\in {\textbf{X}}_{h}$

9.23 $$\begin{aligned} \left| { \left( \!\left( \hspace{-0.50003pt} {\textbf{e}}_{h},{\textbf{v}}_{0} \hspace{-1.00006pt}\right) \!\right) }_k\right| =\left| A_{k}({\textbf{e}}_{h},{\mathcal {N}} _{-k}{\textbf{v}}_{0}-{\textbf{w}}_{h})\right| \le \left\| {\textbf{e}} _{h}\right\| _{{\text {imp}},k}\left\| {\mathcal {N}}_{-k} {\textbf{v}}_{0}-{\textbf{w}}_{h}\right\| _{{\text {imp}},k}. \nonumber \\ \end{aligned}$$

We set

9.24 $$\begin{aligned} {\tilde{\eta }}_{2}^{{\text {alg}}}\left( {\textbf{X}}_{h}\right) :=\sup _{{\textbf{v}}_{0}\in {\textbf{V}}_{-k,0}\backslash \left\{ {\textbf{0}} \right\} }\inf _{{\textbf{w}}_{h}\in {\textbf{X}}_{h}}\frac{\left\| {\mathcal {N}}_{-k}{\textbf{v}}_{0}-{\textbf{w}}_{h}\right\| _{{\text {imp}},k}}{\left\| {\textbf{v}}_{0}\right\| _{{\text {imp}},k}} \end{aligned}$$

so that

9.25 $$\begin{aligned} \left| T_{2}\right| =\left| { \left( \!\left( \hspace{-0.50003pt} {\textbf{e}}_{h},{\textbf{v}}_{0} \hspace{-1.00006pt}\right) \!\right) }_k\right| \le {\tilde{\eta }}_{2}^{{\text {alg}}}\left( {\textbf{X}}_{h}\right) \Vert {\textbf{e}}_{h}\Vert _{{\text {imp}},k}\Vert {\textbf{v}}_{0}\Vert _{{\text {imp}},k}. \end{aligned}$$

#### *hp*-Analysis of $T_{2}$

Next, we gauge the approximation property ${\tilde{\eta }}_{2} ^{{\text {alg}}}\left( {\textbf{X}}_{h}\right)$. We employ the splitting given by Theorem 7.3, viz.,

9.26 $$\begin{aligned} {\mathcal {N}}_{-k}{\textbf{v}}_{0}={\textbf{z}}={\textbf{z}}_{H^{2}}+{\textbf{z}} _{{\mathcal {A}}}+k^{-2}\nabla \varphi _{{\textbf{f}}} - {\text {i}}\,k^{-1} \nabla \varphi _{{\textbf{g}}}. \end{aligned}$$

Note that these five functions ${\textbf{z}}$, ${\textbf{z}}_{H^{2}}$, ${\textbf{z}}_{{\mathcal {A}}}$, $\varphi _{{\textbf{f}}}$, $\varphi _{{\textbf{g}}}$ depend on ${\textbf{v}}_{0}$ but we suppress this in the notation. From Theorem 7.3 with $m=m^{\prime }=1$ we have

9.27 $$\begin{aligned} \Vert \varphi _{{\textbf{f}}}\Vert _{H^{2}(\varOmega )}&\le C\left| k\right| ^{2}\Vert {\text {div}}\,{\textbf{v}}_{0}\Vert _{L^{2}(\varOmega )}\overset{{\text {div}}\,{\textbf{v}}_{0}=0}{=}0,\nonumber \\ \Vert \varphi _{{\textbf{g}}}\Vert _{H^{2}(\varOmega )}&\le C|k|\Vert {\text {div}}_{\varGamma }({\textbf{v}}_{0})_{T}\Vert _{H^{-1/2}(\varGamma )}\le C|k|\Vert ({\textbf{v}}_{0})_{T}\Vert _{H^{1/2}(\varGamma )}\overset{(\text {9.20})}{\le }C|k|\Vert {\textbf{v}}_{0}\Vert _{{\text {imp}},k},\nonumber \\ \Vert {\textbf{z}}_{H^{2}}\Vert _{{\textbf{H}}^{2}(\varOmega )}&\le \left| k\right| ^{2}\Vert {\textbf{z}}_{H^{2}}\Vert _{{\textbf{H}}^{2}(\varOmega ),k}\overset{(\text {7.10})}{\le }C|k|^{-1}\left( \Vert k^{2}{\textbf{v}}_{0}\Vert _{{\textbf{H}}^{1}(\varOmega )}+|k|\Vert k({\textbf{v}} _{0})_{T}\Vert _{{\textbf{H}}^{1/2}(\varGamma )}\right) \nonumber \\&\overset{(\text {9.20})}{\le }C|k|\Vert {\textbf{v}}_{0}\Vert _{{\text {imp}},k},\nonumber \\ {\textbf{z}}_{{\mathcal {A}}}&\in {\mathcal {A}}(CC_{{\textbf{z}}},B,\varOmega ),\nonumber \\ C_{{\textbf{z}}}&={(1+C_\mathrm{{stab}})}|k|^{ \theta -1 } \bigl ( \Vert k^{2}{\textbf{v}}_{0}\Vert +|k|\Vert k({\textbf{v}}_{0})_{T} \Vert _{{\textbf{H}}^{-1/2}(\varGamma )}\bigr ) \nonumber \\&\overset{{\text {i}}\,k({\textbf{v}}_{0})_{T}={\text {div}}_{\varGamma }\,{\textbf{v}}_{0},(\text {9.20})}{\le }C|k|^{ \theta } \Vert {\textbf{v}}_{0}\Vert _{{\text {imp}},k}. \end{aligned}$$

We note that $\varphi _{{\textbf{f}}}=0$. For the approximation of $\nabla \varphi _{{\textbf{g}}}$, we use the elementwise defined operator $\varPi _{p}^{\nabla ,s}$ of Corollary 8.4 with $m=2$ there to get

9.28 $$\begin{aligned} \bigl \Vert k^{-1}\bigl ( \nabla \varphi _{{\textbf{g}}}-\nabla \varPi _{p}^{\nabla ,s} \varphi _{{\textbf{g}}}\bigr ) \bigr \Vert _{{\text {imp}},k}&\le C|k|^{-1}\left( |k|\frac{h}{p}+|k|^{1/2}\left( \frac{h}{p}\right) ^{1/2}\right) \Vert \varphi _{{\textbf{g}}}\Vert _{H^{2}(\varOmega )}\nonumber \\&\le C\left( \frac{|k|h}{p}\right) ^{1/2}\Vert {\textbf{v}}_{0} \Vert _{{\text {imp}},k}. \end{aligned}$$

For the approximation of ${\textbf{z}}_{H^{2}}$, we employ the elementwise defined operator $\varPi _{p}^{{\text {curl}},s}:{\textbf{H}}^{2} (\varOmega )\rightarrow {\textbf{X}}_{h}$ as in Lemma 8.5. By summing over all elements the estimates of Lemma 8.5 (i) we get

9.29 $$\begin{aligned}&\Vert {\textbf{z}}_{H^{2}}-\varPi _{p}^{{\text {curl}},s} {\textbf{z}}_{H^{2}}\Vert _{{\textbf{H}}\left( {\text {curl}} ,\varOmega \right) ,k}^{2}=\sum _{K\in {\mathcal {T}}_{h}}\Vert {\textbf{z}} _{H^{2}}-\varPi _{p}^{{\text {curl}},s}{\textbf{z}}_{H^{2}}\Vert _{{\textbf{H}}( {\text {curl}},K) ,k}^{2} \nonumber \\&\quad \le C\sum _{K\in {\mathcal {T}}_{h}}\frac{h_{K}^{2}}{p^{2}}\left( 1+\frac{\left| k\right| ^{2}h_{K}^{2}}{p^{2}}\right) \Vert {\textbf{z}}_{H^{2}}\Vert _{{\textbf{H}}^{2}( K) }^{2}\le C\frac{h^{2}}{p^{2}}\left( 1+\frac{\left| k\right| ^{2}h^{2}}{p^{2} }\right) \Vert {\textbf{z}}_{H^{2}}\Vert _{{\textbf{H}}^{2}( \varOmega ) }^{2}\nonumber \\&\quad \le C\frac{\left| k\right| ^{2}h^{2}}{p^{2}}\left( 1+\frac{\left| k\right| ^{2}h^{2}}{p^{2}}\right) \left\| {\textbf{v}}_{0}\right\| _{{\text {imp}},k}^{2}. \end{aligned}$$

For the boundary part of the $\left\| \cdot \right\| _{{\text {imp}},k}$ norm we proceed similarly using Lemma 8.5(i) to arrive at

$$\begin{aligned} |k|^{1/2}\Vert {\textbf{z}}_{H^{2}}-\varPi _{p}^{{\text {curl}},s} {\textbf{z}}_{H^{2}}\Vert _{{\textbf{L}}^{2}(\varGamma )}\le \! C|k|^{1/2}\left( \frac{h}{p}\right) ^{3/2}\!\!\! \Vert {\textbf{z}}_{H^{2}}\Vert _{{\textbf{H}}^{2} (\varOmega )}\le \! C\! \left( \frac{|k|h}{p}\right) ^{3/2}\!\!\! \Vert {\textbf{v}}_{0} \Vert _{{\text {imp}},k}. \end{aligned}$$

In summary, we have proved

9.30 $$\begin{aligned} \Vert {\textbf{z}}_{H^{2}}-\varPi _{p}^{{\text {curl}},s}{\textbf{z}} _{H^{2}}\Vert _{{\text {imp}},k}\le C\frac{ | k| h }{p}\left( 1+\left( \frac{h\left| k\right| }{p}\right) ^{1/2}+\frac{h\left| k\right| }{p}\right) \left\| {\textbf{v}} _{0}\right\| _{{\text {imp}},k}.\nonumber \\ \end{aligned}$$

Next, for the analytic part ${\textbf{z}}_{{\mathcal {A}}}$ we get from Lemma 8.5(iii) in view of (9.27) under the (mild) resolution condition

9.31 $$\begin{aligned} h+\frac{\left| k\right| h}{p}\le {\widetilde{C}} \end{aligned}$$

that

9.32 $$\begin{aligned} \Vert {\textbf{z}}_{{\mathcal {A}}}-\varPi _{p}^{{\text {curl}},s}{\textbf{z}} _{{\mathcal {A}}}\Vert _{{\text {imp}},k}&\le CC_{{\textbf{z}}}\left| k\right| \left( \left( \frac{h}{h+\sigma }\right) ^{p}+\left( \frac{|k|h}{\sigma p}\right) ^{p}\right) \nonumber \\&\le C\left\| {\textbf{v}}_{0}\right\| _{{\text {imp}},k}|k|^{ \theta +1 } \left( \left( \frac{h}{h+\sigma }\right) ^{p}+\left( \frac{|k|h}{\sigma p}\right) ^{p}\right) . \end{aligned}$$

This derivation is summarized in the following lemma.

##### Lemma 9.5

Assume hypothesis (3.2) and let $\varOmega$ be a bounded Lipschitz domain with simply connected, analytic boundary. Let the mesh satisfy Assumption 8.1. Let $c_{2}$, $\varepsilon >0$ be given. Then there exists $c_{1}>0$ (depending only on the constants of (3.2), $\varOmega$, the parameters of Assumption 8.1, and $c_{2}$, $\varepsilon$) such that for *h*, *k*, *p* satisfying the resolution condition

9.33 $$\begin{aligned} \frac{\left| k\right| h}{p}\le c_{1}\quad \text {and}\quad p\ge \max \left\{ 1,c_{2}\ln \left| k\right| \right\} \end{aligned}$$

there holds

9.34 $$\begin{aligned} {\tilde{\eta }}_{2}^{{\text {alg}}}\left( {\textbf{X}}_{h}\right) \le \varepsilon . \end{aligned}$$

##### Proof

We combine (9.28), (9.30), and (9.32) with the resolution condition to arrive at

9.35 $$\begin{aligned} {\tilde{\eta }}_{2}^{{\text {alg}}}\left( {\textbf{X}}_{h}\right) \le C\left( \left( \frac{h\left| k\right| }{p}\right) ^{1/2} +{\left| k\right| ^{ \theta +1} } \left[ \left( \frac{h}{\sigma +h}\right) ^{p}+\left( \frac{\left| k\right| h}{\sigma p}\right) ^{p}\right] \right) . \end{aligned}$$

Clearly, by selecting $c_1$ sufficiently small, we may ensure that the first term in (9.35), $(|k| h/p)^{1/2}$, is smaller than $\varepsilon /3$. The second term in (9.35), $|k|^{\theta +1} (h/(\sigma + h))^p$, can be made smaller than $\varepsilon /3$ for sufficiently small $c_1$ by appealing to [39, Lem. 8.7]. For the last term in (9.35), we may assume that $c_1 < \sigma$ and then estimate

$$\begin{aligned} |k|^{\theta +1} \left( \frac{|k| h}{\sigma p}\right) ^{p}&\le |k|^{\theta +1} (c_1/\sigma )^p \le |k|^{\theta +1} (c_1/\sigma )^{\max \{1,c_2 \ln |k|\}} \\&= \min \{|k|^{\theta +1} (c_1/\sigma ), |k|^{\theta +1 + c_2 \ln (c_1/\sigma )}\}. \end{aligned}$$

This expression can be made smaller than $\varepsilon /3$ uniformly in $|k| \in [1,\infty )$ by selecting $c_1$ sufficiently small: the first term in the minimum tends to 0 as $c_1\rightarrow 0$ uniformly in $|k| \in [1,2]$ and the second term in the minimum tends to zero as $c_1 \rightarrow 0$ uniformly in $|k| \ge 2$. $\square$

### *h*-*p*-*k*-Explicit Stability and Convergence Estimates for Maxwell’s Equations

We begin with the estimate of the consistency term $\delta _{k}$.

#### Lemma 9.6

Let the assumptions in Lemma 9.5 hold. Let $\varepsilon$, $c_{2}>0$ be given. Then, one can choose a constant $c_{1}>0$ sufficiently small such that the resolution condition (9.33) implies

$$\begin{aligned} \delta _{k}({\textbf{e}}_{h})\le \varepsilon . \end{aligned}$$

#### Proof

We combine estimates (9.8), (9.9), (9.15), (9.19), (9.25) for ${\textbf{v}}_{0}:=\varPi _{-k}^{{\text {curl}}}{\textbf{v}}_{h}$, Lemma 9.3, and (9.34) in a straightforward way to obtain

$$\begin{aligned} \left| { \left( \!\left( \hspace{-0.50003pt} {\textbf{e}}_{h},{\textbf{v}}_{h} \hspace{-1.00006pt}\right) \!\right) }_k\right| \le C\bigl ( c_{1}^{1/2}+c_{1}+\varepsilon \bigr ) \left\| {\textbf{e}}_{h}\right\| _{{\text {imp}},k}\left\| {\textbf{v}}_{h}\right\| _{{\text {imp}},k} \end{aligned}$$

and thus $\displaystyle \delta _{k}({\textbf{e}}_{h})\le 2C(c_{1}^{1/2} +c_{1}+\varepsilon ).$ We may assume that $\sqrt{c_{1}}\le \varepsilon \le 1$ so that $\displaystyle \delta _{k}({\textbf{e}}_{h})\le 6C\varepsilon$. Since the constant $C>0$ does not depend on $\varepsilon$, the result follows by adjusting constants. $\square$

This estimate allows us to formulate the quasi-optimality of the *hp*-FEM Galerkin discretization and to show *h*-*p*-*k*-explicit convergence rates under suitable regularity assumptions.

#### Theorem 9.7

Let $\varOmega \subset {\mathbb {R}}^{3}$ be a bounded Lipschitz domain with a simply connected, analytic boundary. Let the stability Assumption (3.2) be satisfied. Let the finite element mesh with mesh size *h* satisfy Assumption 8.1, and let ${\textbf{X}}_{h}$ be defined by as the space of Nédélec-type-I elements of degree *p* (cf. (8.7)).

Then, for any ${\textbf{j}}$, ${\textbf{g}}_{T}$ satisfying (2.38), the variational form of Maxwell’s equations (2.39) has a unique solution ${\textbf{E}}$.

For any fixed $c_{2}>0$ and $\eta \in (0,1)$ one can select $c_{1}>0$ (depending only on $\varOmega$ and the constants of (3.2) and Assumption 8.1) such that the resolution condition (9.33) implies that the discrete problem (8.9) has a unique solution ${\textbf{E}}_{h}$, which satisfies the quasi-optimal error estimate

9.36 $$\begin{aligned} \left\| {\textbf{E}}-{\textbf{E}}_{h}\right\| _{{\text {imp}},k}\le \frac{1+\eta }{1-\eta }\inf _{{\textbf{w}}_{h} \in {\textbf{X}}_{h}}\left\| {\textbf{E}}-{\textbf{w}}_{h}\right\| _{{\text {imp}},k}. \end{aligned}$$

#### Proof

Existence and uniqueness of the continuous variational Maxwell problem follow from Proposition 3.1. From Lemma 9.6 we know that $c_{1}$ can be chosen sufficiently small such that $\delta ({\textbf{e}}_{h})<\eta$. As in the proof of Theorem [39, Thm. 4.15] (which goes back to [27, Thm. 3.9]) existence, uniqueness, and quasi-optimality follows. $\square$

The quasi-optimality result (9.36) leads to quantitative, *k*-explicit error estimates if a *k*-explicit regularity of the solution ${\textbf{E}}$ is available. In the following corollary, we draw on the regularity assertions of Theorem 7.3. We point out, however, that due to our relying on the operator $\varPi _{p}^{{\text {curl}},s}$ and the regularity assertion Theorem 7.3, the regularity requirements on the data ${\textbf{j}}$, ${\textbf{g}}_{T}$ are not the weakest possible ones.

#### Corollary 9.8

Let the hypotheses of Theorem 9.7 be valid. Given $\eta \in (0,1)$ and $c_{2}>0$ let $c_{1}$ be as in Theorem 9.7. Then, under the scale resolution condition (9.33) the following holds: Let *m*, $m^{\prime }\in {\mathbb {N}}_{0}$ and $({\textbf{j}},{\textbf{g}}_{T})\in {\textbf{H}}^{m}(\varOmega )\times {\textbf{H}}^{m-1/2}(\varGamma )$ together with $({\text {div}}\,{\textbf{j}},{\text {div}}_{\varGamma }\,{\textbf{g}}_{T} )\in {\textbf{H}}^{m^{\prime }}(\varOmega )\times {\textbf{H}}^{m^{\prime }-1/2}(\varGamma )$. If $p\ge \max (m,m^{\prime })$, then

9.37 $$\begin{aligned} \Vert {\textbf{E}}-{\textbf{E}}_{h}\Vert _{{\text {imp}},k}&\le C\frac{1+\eta }{1-\eta }\Biggl \{C_{{\textbf{j}},{\textbf{g}},m}\left( \frac{h}{p}\right) ^{m}+C_{{\textbf{j}},{\textbf{g}},m^{\prime }}|k|^{-3/2}\left( \frac{h}{p}\right) ^{m^{\prime }+1/2}\nonumber \\&\quad +|k|C_{{\textbf{j}},{\textbf{g}},{\mathcal {A}}}\left( \left( \frac{h}{h+\sigma }\right) ^{p}+\left( \frac{|k|h}{\sigma p}\right) ^{p}\right) \Biggr \}, \end{aligned}$$

where

9.38 $$\begin{aligned} C_{{\textbf{j}},{\textbf{g}},m}&:=|k|^{-1}\Vert {\textbf{j}}\Vert _{{\textbf{H}} ^{m}(\varOmega )}+\Vert {\textbf{g}}_{T}\Vert _{{\textbf{H}}^{m-1/2}(\varGamma )}, \end{aligned}$$

9.39 $$\begin{aligned} C_{{\textbf{j}},{\textbf{g}},m^{\prime }}&:=\Vert {\text {div}}\,{\textbf{j}}\Vert _{{\textbf{H}}^{m^{\prime }}(\varOmega )}+|k|\Vert {\text {div}} _{\varGamma }\,{\textbf{g}}_{T}\Vert _{{\textbf{H}}^{m^{\prime }-1/2}(\varGamma )}, \end{aligned}$$

9.40 $$\begin{aligned} C_{{\textbf{j}},{\textbf{g}},{\mathcal {A}}}&:=\left| k\right| ^{ \theta -1} \left( \Vert {\textbf{j}}\Vert _{{\textbf{L}}^{2}(\varOmega )}+|k|\Vert {\textbf{g}} _{T}\Vert _{{\textbf{H}}^{-1/2}(\varGamma )}\right) . \end{aligned}$$

#### Proof

For the error estimate (9.37), we employ the solution decomposition provided by Theorem 7.3:

$$\begin{aligned} {\textbf{E}}={\textbf{E}}_{H^{2}}+{\textbf{E}}_{{\mathcal {A}}}+k^{-2}\nabla \varphi _{{\textbf{j}}}+{\text {i}}\,k^{-1}\nabla \varphi _{{\textbf{g}}} \end{aligned}$$

with

$$\begin{aligned} \Vert {\textbf{E}}_{H^{2}}\Vert _{{\textbf{H}}^{m+1}(\varOmega )}&\le CC_{{\textbf{j}} ,{\textbf{g}},m},\\ \Vert \varphi _{{\textbf{j}}}\Vert _{H^{m^{\prime }+2}(\varOmega )}+|k|\Vert \varphi _{{\textbf{g}}}\Vert _{H^{m^{\prime }+2}(\varOmega )}&\le CC_{{\textbf{j}} ,{\textbf{g}},m^{\prime }},\\ {\textbf{E}}_{{\mathcal {A}}}&\in {\mathcal {A}}(CC_{{\textbf{j}},{\textbf{g}} ,{\mathcal {A}}},B,\varOmega ) \end{aligned}$$

for *k*-independent constants *C*, *B*. With the operators $\varPi _{p}^{\nabla ,p}$ and $\varPi _{p}^{{\text {curl}},s}$ of Corollary 8.4 and Lemma 8.5 we get

$$\begin{aligned} \Vert {\textbf{E}}_{H^{2}}-\varPi _{p}^{{\text {curl}},s}{\textbf{E}}_{H^{2}} \Vert _{{\text {imp}},k}&\! \le \! CC_{{\textbf{j}},{\textbf{g}},m}\!\left[ \! \left( \frac{h}{p}\right) ^{m}\!\!+|k|\left( \frac{h}{p}\right) ^{m+1} \!\!+|k|^{1/2}\left( \frac{h}{p}\right) ^{m+1/2}\right] \\&\le CC_{{\textbf{j}},{\textbf{g}},m}\left( \frac{h}{p}\right) ^{m},\\ \left| k\right| ^{-2}\Vert \nabla \varphi _{{\textbf{j}}}-\nabla \varPi _{p}^{\nabla ,s}\varphi _{{\textbf{j}}}\Vert _{{\text {imp}},k}&\le \! CC_{{\textbf{j}},{\textbf{g}},m^{\prime }}|k| ^{-2}\!\left[ \! |k|\left( \frac{h}{p}\right) ^{m^{\prime }+1}+|k|^{1/2}\left( \frac{h}{p}\right) ^{m^{\prime }+1/2}\right] \\&\le CC_{{\textbf{j}},{\textbf{g}},m^{\prime }}\left| k\right| ^{-2}|k|^{1/2}\left( \frac{h}{p}\right) ^{m^{\prime }+1/2},\\ |k|^{-1}\Vert \nabla \varphi _{{\textbf{g}}}-\nabla \varPi _{p}^{\nabla ,s}\varphi _{{\textbf{g}}}\Vert _{{\text {imp}},k}&\le CC_{{\textbf{j}},{\textbf{g}},m^{\prime }}\left| k\right| ^{-2} |k|^{1/2}\left( \frac{h}{p}\right) ^{m^{\prime }+1/2},\\ \Vert {\textbf{E}}_{{\mathcal {A}}}-\varPi _{p}^{{\text {curl}},s}{\textbf{E}} _{{\mathcal {A}}}\Vert _{{\text {imp}},k}&\le C|k|C_{{\textbf{j}} ,{\textbf{g}},{\mathcal {A}}}\left( \left( \frac{h}{h+\sigma }\right) ^{p}+\left( \frac{|k|h}{\sigma p}\right) ^{p}\right) . \end{aligned}$$

$$\square $$

**Fig. 1:**
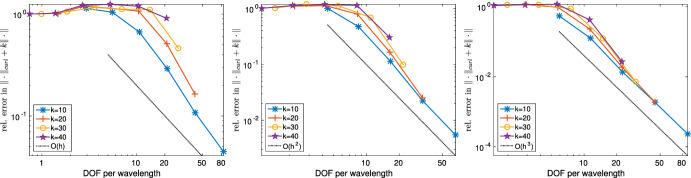
$\varOmega =(-1,1)^{3}$, smooth solution; left to right: $p\in \{1,2,3\}$

**Fig. 2:**
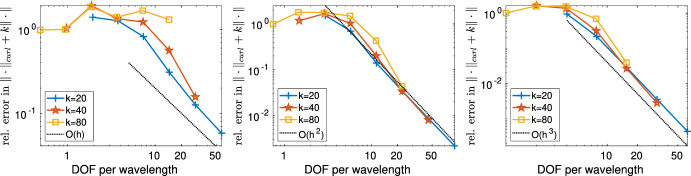
$\varOmega = (-1,1)^{3}\setminus [-1/2,1/2]^{3}$, smooth solution; left to right: $p \in \{1,2,3\}$

## Numerical Results

We illustrate the theoretical findings of Theorem 9.7 and Corollary 9.8 by two numerical experiments. All computations are performed with NGSolve, [47, 48] using Nédélec type II elements, i.e., full polynomial spaces.

### Remark 10.1

While the analysis of the present paper is peformed in detail for Nédélec type I elements, it can be extended to Nédélec type II elements. Key is the observation that commuting diagram operators ${\widehat{\varPi }}^{grad,c}_{p+1}$ and ${\widehat{\varPi }}^{curl,c}_p$ analogous to the ones used in Sects. 8 and 9 for type I elements also exist for type II elements. This is discussed in [46, Sec. 4.8].$\square$

We show in Figs. 1 and 2 the relative error in the norm $\Vert {\text {curl}}\cdot \Vert _{{\textbf{L}}^{2}(\varOmega )}+\left| k\right| \Vert \cdot \Vert _{{\textbf{L}}^{2}(\varOmega )}\sim \Vert \cdot \Vert _{{\textbf{H}}({\text {curl}},\varOmega ),\left| k\right| }$ versus the number of degrees of freedom per wavelength

$$\begin{aligned} N_{\lambda }=\frac{2\pi {\text {DOF}}^{1/3}}{|k||\varOmega |^{1/3}}, \end{aligned}$$

where ${\text {DOF}}$ stands for the dimension of the ansatz space.

### Example 10.2

We consider $\varOmega =(-1,1)^{3}$ and impose the right-hand side and the impedance boundary conditions in such a way that the exact solution is ${\textbf{E}}\left( {\textbf{x}}\right) ={\text {curl}}\sin (kx_{1} )(1,1,1)^{\top }$. Figure 1 shows the performance for the choices $k\in \{10,20,30,40\}$ and $p\in \{1,2,3\}$ as the mesh is refined quasi-uniformly. The final problem sizes were ${\text {DOF}}=18,609,324$ for $p=1$, ${\text {DOF}}=9,017,452$ for $p=2$, and ${\text {DOF}}=23,052,940$ for $p=3$.

We observe the expected asymptotic $O(h^{p})$ convergence. We also observe that the onset of asymptotic quasi-optimal convergence is reached for smaller values of $N_{\lambda }$ for higher order methods. This is expected in view of Theorem 9.7, although the present setting of a piecewise analytic geometry is not covered by Theorem 9.7. $\square$

### Example 10.3

We consider $\varOmega =(-1,1)^{3}{\setminus } [-1/2,1/2]^{3}$ and Maxwell’s equations with impedance boundary conditions on $\partial (-1,1)^{3}$ and perfectly conducting boundary conditions on the inner boundary $\partial (-1/2,1/2)^{3}$. We prescribe an exact solution ${\textbf{E}}( {\textbf{x}}) =k\cos (kx_{1})(x_{1}^{2}-1/4)(x_{2} ^{2}-1/4)(x_{3}^{2}-1/4)(0,-1,1)^{\top }$. Figure 2 shows the performance for the choices $k\in \{20,40,80\}$ and $p\in \{1,2,3\}$ as the mesh is refined quasi-uniformly. The final problem sizes were ${\text {DOF}}=43,598,374$ for $p=1$, ${\text {DOF}}=168,035,046$ for $p=2$, and ${\text {DOF}} =54,063,558$ for $p=3$.

We observe the expected asymptotic $O(h^{p})$ convergence. We also observe that the onset of asymptotic quasi-optimal convergence is reached for smaller values of $N_{\lambda }$ for higher order methods. $\square$

## Footnotes

1 We exclude here a neighborhood of 0 since we are interested in the high-frequency behavior – to simplify notation we have fixed $k_{0}=1$ while any other positive choice $k_{0}\in \left( 0,1\right)$ leads to qualitatively the same results while constants then depend continuously on $k_{0}\in \left( 0,1\right)$ and, possibly, deteriorate as $k_{0}\rightarrow 0$.

2 Throughout the paper we use the convention that if ${\textbf{v}}_{T}$, ${\textbf{v}}_{T}^{\nabla }$, ${\textbf{v}}_{T}^{{\text {curl}}}$, $V^{\nabla }$, $V^{{\text {curl}}}$ appear in the same context they are related by (2.11).

3 We always write $\left| k\right|$ in the estimates also if the exponent is even for the sake of clarity.

4 In [13, Thm. 2], it is shown that these conditions are equivalent for ${{\textbf{u}}}$ with ${{\textbf{u}}}\in {{\textbf{H}} }(\varOmega ,{\text {curl}})\cap H(\varOmega ,{\text {div}})$.

5 We thank an anonymous referee for suggesting this example.

6 With a slight abuse of notation we write ${\mathcal {N}} _{{\text {MW}},k}^{{\text {curl}}}\left( v\right) :=\int _{{\mathbb {R}}^{3}}g_{k}\left( \left\| \mathbf {\cdot }-{\textbf{y}}\right\| \right) v\left( {\textbf{y}}\right) d{\textbf{y}}$ also for scalar functions *v*. This is the classical acoustic Newton potential.

7 We write ${\textbf{L}}_{\varGamma }{\textbf{g}}_{T}^{\prime }$ instead of introducing a new symbol ${\textbf{Z}}$ with ${\textbf{Z}}|_{\varGamma }={\textbf{L}}_{\varGamma }{\textbf{g}}_{T}^{\prime }$

8 Note that ${\textbf{X}}_{h}=\varvec{{\mathcal {N}}}_{p}^{{\text {I}} }({{\mathcal {T}}}_{h})$ and $S_{h}=S_{p+1}({{\mathcal {T}}}_{h})$ since $\varvec{{\mathcal {N}}}_{p}^{{\text {I}}}({{\mathcal {T}}}_{h} )\subset {\textbf{X}}_{{\text {imp}}}$ and $S_{p+1}({{\mathcal {T}}} _{h})\subset H_{{\text {imp}}}^{1}( \varOmega )$.

9 Our choice of notation is motivated by the appearance of similar approximation quantities in the companion paper [39] (for transparent boundary conditions), where various measures of approximability $\eta _{j}^{\exp }$, $j\in \left\{ 1,3,4,5,7\right\}$, and $\eta _{j}^{{\text {alg}}}$, $j\in \left\{ 2,6\right\}$, were introduced and used in the convergence analysis. Here, only the quantities $\eta _{6}^{{\text {alg}} }$ in (9.14) and ${\tilde{\eta }}_{2}^{{\text {alg}}}$ in (9.24) are needed.
